# Hepatocyte-derived LRG1 primes the liver for metastasis and impairs immunotherapy

**DOI:** 10.1038/s41423-026-01408-9

**Published:** 2026-04-10

**Authors:** Guojie Long, Bing Cheng, Yue Jiang, Qiufeng Liu, Xiaoming Huang, Zhitong Niu, Qian Xiao, Xiangjun Qian, Chaoyin Wei, Jinxin Chen, Yingzhen Weng, Zheyu Zheng, Dandong Luo, Tao Ma, Ting Su, Qiongwei Tang, Panpan Wang, Yufeng Chen, Jing Tan, Ronghua Zhang, Qiang Yu, Weidong Pan, Wenyu Wang

**Affiliations:** 1 Guangdong Provincial Key Laboratory of Colorectal and Pelvic Floor Diseases, Department of General Surgery (Department of Pancreatic Hepatobiliary Surgery), Guangdong Institute of Gastroenterology, Biomedical Innovation Center, The Sixth Affiliated Hospital, Sun Yat-sen University, Guangzhou, 510655 China; 2 State Key Laboratory of Oncology in South China, Guangdong Provincial Clinical Research Center for Cancer, Sun Yat-sen University Cancer Center, Guangzhou, 510060 China; 3 Department of Hepatobiliary Pancreatic Surgery, The Affiliated Cancer Hospital of Zhengzhou University & Henan Cancer Hospital, Zhengzhou, 450008 China; 4 Department of Traditional Chinese Medicine, The Sixth Affiliated Hospital, Sun Yat-sen University, Guangzhou, 510655 China; 5 The First Affiliated Hospital of Jinan University, Guangzhou, 510630 China; 6 Laboratory of Cancer Epigenome, Division of Medical Sciences, National Cancer Centre, Singapore, 168583 Singapore; 7 Hainan Academy of Medical Science, Hainan Medical University, Haikou, 571199 China; 8 Pharmacy college, Jinan University, Guangzhou, 510632 China; 9 Cancer research institute, Jinan University, Guangzhou, 510632 China; 10 Genome Institute of Singapore, Agency for Science, Technology, and Research (A*STAR), Biopolis, 138672 Singapore, Singapore; 11 Department of Physiology, Yong Loo Lin School of Medicine, National University of Singapore, Singapore, 117597 Singapore; 12 Cancer and Stem Cell Biology, Duke-NUS Medical School, Singapore, 169857 Singapore; 13 Tianfu Jincheng Lab, Chengdu, 610041 China

**Keywords:** Leucine-rich alpha-2 glycoprotein 1(LRG1), Liver metastasis, Pre-metastatic niche, Neutrophil extracellular traps (NETs), Immunotherapy sensitivity, Cancer microenvironment, Colon cancer

## Abstract

The liver undergoes active remodeling by the primary tumor prior to metastatic spread. However, the mechanisms by which hepatocytes dictate the liver-specific tropism of tumors remain elusive. Here, we identify hepatocyte-derived leucine-rich alpha-2-glycoprotein 1 (LRG1) as a key mediator of liver premetastatic niche (PMN) formation. Clinically, elevated serum LRG1 levels are correlated with an increased risk of liver metastasis in patients and multiple mouse models. Mechanistically, LRG1 remodels the hepatic microenvironment by driving immunosuppressive neutrophil accumulation, impairing the function of effector T cells and dendritic cells, and enhancing angiogenesis in the liver, thereby fostering a prometastatic landscape. Hepatocyte-specific ablation of LRG1 dampens premetastatic niche formation and significantly reduces the metastatic burden in vivo. Hepatic LRG1 induced by tumor-associated inflammation via IL-6/STAT3 signaling promotes liver metastasis through the formation of TGFBR/PI3K/AKT axis-driven neutrophil extracellular traps (NETs). Importantly, therapeutic blockade of LRG1 not only suppressed liver metastasis but also reprogrammed the hepatic niche toward an immune-activated state, sensitizing tumors to anti-PD-1 therapy. Collectively, our findings reveal a hepatocyte–LRG1 axis that drives liver premetastatic niche remodeling and highlight LRG1 as a promising target for the prevention and treatment of liver metastasis.

## Introduction

The liver is among the most common sites for metastatic dissemination across multiple cancer types, including colorectal cancer, and accounts for a significant proportion of cancer-related mortality worldwide [1]. Emerging evidence highlights the critical role of premetastatic niche (PMN) formation, a process in which primary tumors systemically prime distant organs to favor metastatic seeding and outgrowth [2–4]. Rather than being a passive recipient, the liver—with its unique vasculature and immune environment—undergoes active remodeling during PMN development [5]. However, the mechanisms by which hepatic cells, particularly hepatocytes, orchestrate this tumor-supportive microenvironment remain poorly defined, hindering targeted therapeutic strategies.

Leucine-rich α-2-glycoprotein 1 (LRG1), a secreted glycoprotein, contributes to a wide range of human diseases [6, 7], including cancers. Elevated LRG1 expression in tumors is correlated with cancer progression, tumor burden, and poor prognosis [8–13]. LRG1 is produced by diverse cellular sources. Tumor cell-derived LRG1 has been shown to increase tumor proliferation [14] and modulate angiogenesis [15] and, as demonstrated in our previous work, promote epithelial‒mesenchymal transition (EMT) in an autocrine manner [16]. Additionally, endothelial cell-derived LRG1 facilitates lung metastasis by increasing the abundance of neural/glial antigen 2 (NG2)^+^ pericytes and modulating the vascular niche [8]. Among all sources, hepatocytes represent the predominant origin of systemic LRG1. This is particularly significant as the liver—a frequent metastatic target—undergoes extensive remodeling in response to primary tumor signals before metastatic colonization. However, the dynamic expression and role of liver-derived LRG1 in this context remain largely unexplored.

LRG1 exerts most of its pathogenic effects on the vasculature; however, whether LRG1 also contributes to liver metastasis via nonvascular mechanisms remains unclear. In particular, whether hepatocyte-derived LRG1 regulates the formation or function of the premetastatic niche (PMN), a critical determinant of metastatic organotropism [3], is completely unknown. Understanding this axis may reveal novel insights into systemic crosstalk between primary tumors and metastatic organs and reveal potential therapeutic targets for metastatic prevention.

Here, we investigate the hypothesis that hepatocyte-derived LRG1 serves as a central mediator of PMN formation in the liver, bridging systemic tumor-derived signals to local immunosuppressive and prometastatic alterations. Combining clinical observations with mechanistic studies in murine models, we demonstrate that tumor-induced inflammatory cues drive hepatocytes to secrete LRG1, which in turn recruits myeloid cells and triggers NET formation. We further investigated the translational potential of targeting this axis and revealed that LRG1 blockade not only inhibits liver metastasis but also synergizes with immune checkpoint inhibitors by reshaping the hepatic immune microenvironment. These findings position the hepatocyte-LRG1 pathway as a critical determinant of liver tropism in metastasis and reveal new opportunities for therapeutic intervention.

## Results

### Serum LRG1 predicts liver metastasis and is associated with premetastatic niche formation in the liver

**Fig. 1:**
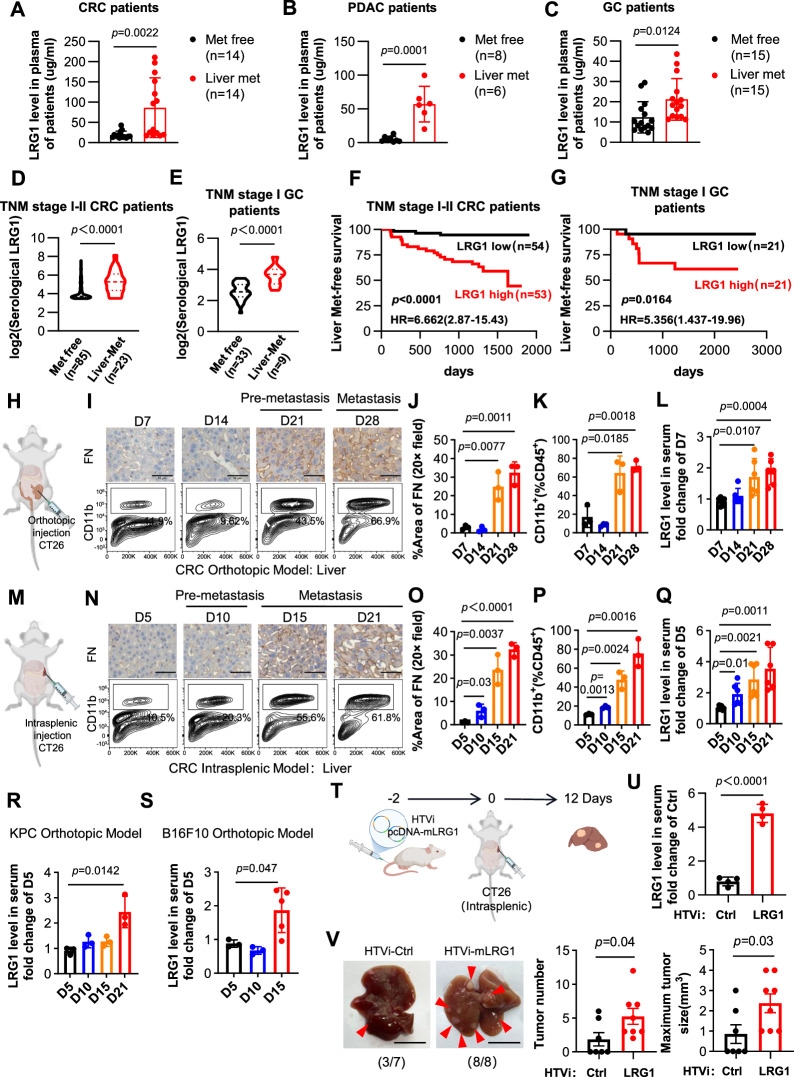
Serological LRG1 predicts liver metastasis and is associated with pre-metastatic niche formation in liver. **A–C** Serological LRG1 levels in patients without metastasis or with liver metastasis. **A** Colorectal cancer patients. **B** PDAC patients. **C** Gastric cancer patients. Data are means ± SD. Serological LRG1 levels in TNM stage I-II CRC patients **D** and TNM stage I. GC patients **E** with or without subsequent liver metastasis during follow-up. Liver metastasis-free survival analysis of TNM stage I-II CRC cohort **F** and. TNM stage I GC cohort **G**. Schematic of CRC orthotopic model in BALB/c **H**. Representative images of IHC staining of FN and flow cytometry analyses of the percentages of CD11b+ cells in CD45+ immunocytes in the liver on different time points. Scale bars, 50 μm **I**. Quantification of FN **J** and quantification of CD11b+ cells **K**, *n* = 3 mice per time point. Data are means ± SD. **L** ELISA analysis of serum LRG1 levels from CRC orthotopic mouse model on days 7, 14, 21, and 28. *n* = 6 mice per time point from two independent experiments and data are means ± SD. Schematic of CRC intrasplenic model in BALB/c **M**. Representative images of IHC staining of FN and flow cytometry analyses of the percentages of CD11b+ cells in CD45+ immunocytes in the liver. Scale bars, 50 μm **N**. Quantification of FN **O** and quantification of CD11b+ cells **P**, *n* = 3 mice per time point. Data are means ± SD. **Q** ELISA analysis of serum LRG1 levels from CRC intrasplenic model on day 5, 10, 15, and 21. *n* = 6 mice per time point from two independent experiments and data are means ± SD. **R** ELISA analysis of serum LRG1 levels from PDAC orthotopic model on day 5, 10, 15, and 21. *n* = 3 mice per time point and data are means ± SD. **S** ELISA analysis of serum LRG1 levels from melanoma orthotopic model on day 5 (*n* = 3), 10 (*n* = 3), and 15 (*n* = 5). Data are means ± SD. Schematic of experimental design **T**. ELISA analysis of serum LRG1 levels 3 days after HTVi plasmid delivery. *n* = 4 and data are means ± SD **U**. Representative images and quantification of liver metastases (number and maximum tumor size) in HTVi-LRG1 (*n* = 7) and HTVi-Ctrl (*n* = 8) groups. **V**. Scale bars, 1 cm. Data are means ± SEM. Statistical significance was determined using two-tailed unpaired Student’s *t* test **A–E, J–L, O–Q, R, S, U and V** or log-rank test **F–G**

To evaluate the association of serological LRG1 with metastasis, we analyzed serum samples from patients with localized or liver metastatic colorectal cancer (CRC), pancreatic ductal adenocarcinoma (PDAC) or gastric cancer (GC). The results demonstrated that patients with liver metastasis had significantly higher serum LRG1 levels (Fig. 1A–C), suggesting that it is closely associated with liver metastasis. To assess its possible predictive capacity for liver metastasis, we retrospectively analyzed serum LRG1 levels in patients with early-stage cancers (TNM stage I/II for CRC and TNM stage I for GC). Individuals who developed liver metastasis within 5 years displayed significantly elevated baseline LRG1 levels (Fig. 1D, E). Survival analysis further revealed that high LRG1 expression correlated with reduced liver metastasis-free survival (Figs. 1F, G). These findings suggest that elevated LRG1 precedes detectable metastasis, indicating its role in premetastatic niche formation. To validate this hypothesis, we generated a CT26 murine cecal orthotopic inoculation model (Fig. 1H) by harvesting the liver weekly. By day 28, small hepatic metastatic foci were observed in a subset of mice, whereas no liver metastasis was detected at earlier timepoints (day 21) (Fig. S1A), which was defined as the premetastatic phase, which is consistent with our previous reports [17]. This finding was further corroborated by CD11b and fibronectin staining (Fig. 1I–K) and *S100a8*, *S100a9*, and *Mmp9* expression (Fig. S1C–E), which were reported to be significantly enriched in premetastatic livers [18]. Synchronous with the formation of the liver premetastatic niche, serum LRG1 levels markedly increased during the premetastatic phase and further increased upon establishment of metastasis (Fig. 1L). No such phenomenon was observed in the sham-operated controls (Fig. S1F–I). Similarly, in the splenic tumor inoculation model, serum levels of LRG1 were also elevated during the premetastatic phase but progressively increased with increasing hepatic metastasis progression (Fig. 1M–Q, Fig. S1I–K). This trend was further validated in orthotopic pancreatic cancer (KPC) (Fig. 1R and Fig. S2A–C) and melanoma (B16F10) (Fig. 1S and Fig. S2D–F) models, where the liver represents a common metastatic site.

To further investigate whether LRG1 directly induces the premetastatic niche and subsequent liver metastasis, we exogenously expressed LRG1 in the liver via hydrodynamic tail vein injection (HTVi) [19] prior to the splenic inoculation of tumor cells (Fig. 1T). Indeed, LRG1 overexpression strongly induced fibronectin deposition in the liver (Fig. S2G, H). On day 12 postinoculation, compared with their control counterparts, mice with LRG1 overexpression displayed significantly more hepatic metastatic foci (Fig. 1U, V). Collectively, these findings suggest that LRG1 facilitates hepatic metastasis by shaping the premetastatic liver microenvironment prior to tumor cell arrival.

### Hepatocyte-derived LRG1 drives liver metastasis

**Fig. 2:**
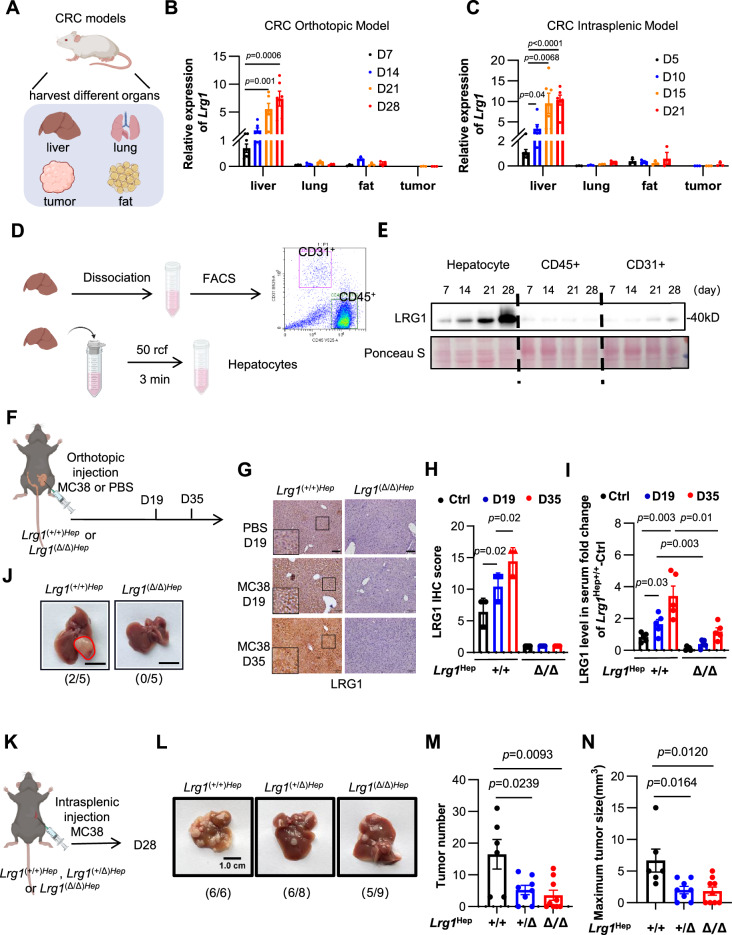
LRG1 is derived from hepatocytes and promotes CRLM. **A–C** Quantification of *Lrg1* expression in different organs from CRC mouse models. *Lrg1* expression in organs from the CRC orthotopic model on day 7, 14, 21, and 28 **B** or from the CRC intrasplenic model on days 5, 10, 15, and 21, measured by qRT-PCR and normalized to *Actb* **C**. Dots represent individual samples. *n* = 6 for liver from two independent experiments. *n* = 3 mice for lung, fat, and tumor per time point. Data are means ± SEM. **D, E** Western blot analysis of LRG1 expression in hepatocytes, CD45+ cells, and CD31+ cells isolated from liver at indicated time points. **F** Schematic of CRC orthotopic model using *Lrg1*(+/+)Hep and *Lrg1*(Δ/Δ)Hep mice. **G, H** Representative images and quantification of IHC staining for LRG1 in liver from different groups as indicated in Fig. 2F. *n* = 5. Scale bars, 50 μm. **I** ELISA analysis of serum LRG1 levels from different groups as indicated in Fig. 2F. *n* = 5. Data are means ± SD. **J** Representative images and percentage of liver metastases at day 35. *n* = 5. Scale bars, 1 cm. **K** Schematic of CRC intrasplenic model using *Lrg1*(+/+)Hep (*n* = 6), *Lrg1*(+/Δ)Hep (*n* = 8) and *Lrg1*(Δ/Δ)Hep (*n* = 9) mice. **L** Representative images of liver metastases from different groups as indicated in Fig. 2K. Scale bars, 1 cm. Quantification of liver metastasis number **M** and maximum tumor size **N** from different groups as indicated in Fig. 2K. Data are means ± SEM. *p* values were obtained by two-tailed unpaired Student’s *t* test

LRG1 is expressed and secreted by diverse types of cells, including hepatocytes [20], adipocytes [21], tumor cells [16], and vascular endothelial cells [8, 22]. To identify the source of circulating LRG1 during liver premetastatic niche formation, we analyzed LRG1 expression across tissues in both orthotopic and intrasplenic murine models established from colorectal cancer, melanoma and KPC cell lines. (Fig. 2A). LRG1 expression was not only significantly upregulated in the premetastatic liver but also progressively increased with tumor dissemination (Fig. 2B, C and Fig. S3A–C), in contrast to the stable levels observed in the sham-operated controls (Fig. S3D, E). This phenomenon was also consistent in the PDAC PMN (Fig. S3F). Although LRG1 expression increased modestly in other tissues, the levels remained significantly lower than those in the liver (Fig. S3G). To further elucidate the primary cellular source of LRG1 in the premetastatic liver, we dissociated hepatic tissues and isolated hepatocytes, immune cells (CD45^+^), and endothelial cells (CD31^+^) (Fig. 2D). The highest level of LRG1 expression was detected in hepatocytes, whose expression increased with metastasis (Fig. 2E). These findings were further confirmed by our previous single-cell RNA sequencing (scRNA-seq) data of premetastatic liver tissues [17] (Fig. S3H) and by IHC staining of LRG1 in premetastatic model mice (Fig. S3I–P). Collectively, these results demonstrate that the expression of hepatocyte-derived LRG1 is strongly induced during metastasis and is strongly associated with the establishment of the premetastatic niche.

To investigate the role of hepatocyte-derived LRG1 in metastasis, we generated *Lrg1*^flox/flox^; Alb-Cre (hereafter referred to as *Lrg1*^(Δ/Δ)Hep^) mice to conditionally knock out *Lrg1* in the liver (Fig. S4A–C) or *Lrg1*^wt/wt^; Alb-Cre (hereafter referred to as *Lrg1*^(+/+)Hep^) mice and then orthotopically implanted them with MC38 colon cancer cells (Fig. 2F). Over time (D19, D35), *Lrg1*^(+/+)Hep^ mice showed progressive increases in hepatic LRG1 expression and serum LRG1 levels, whereas hepatocyte-specific *Lrg1* knockout dramatically suppressed circulating LRG1 elevation (Fig. 2G, H, I), confirming that hepatocytes are the primary source of serum LRG1 during metastatic progression. Although orthotopic MC38 tumors exhibited low hepatic metastasis rates because of excessive primary tumor burden (2/5 by day 35), *Lrg1* deletion completely abolished metastasis (Fig. 2J). In the splenic metastasis model (which induces increased liver metastasis frequency), *Lrg1* knockout also significantly reduced the metastatic burden and tumor size (Fig. 2K–N). Interestingly, the knockout of hepatocyte-derived LRG1 also significantly inhibited the growth of orthotopic tumors (Fig. S4D), which may have been due to the interaction between serum LRG1 levels and orthotopic tumors. This warrants further investigation. These findings demonstrate that hepatocytes serve as the primary source of serum LRG1 during hepatic metastasis progression and that elevated levels of hepatocyte-derived LRG1 critically drive liver metastasis.

### Hepatic LRG1 promotes premetastatic niche formation in the liver

**Fig. 3:**
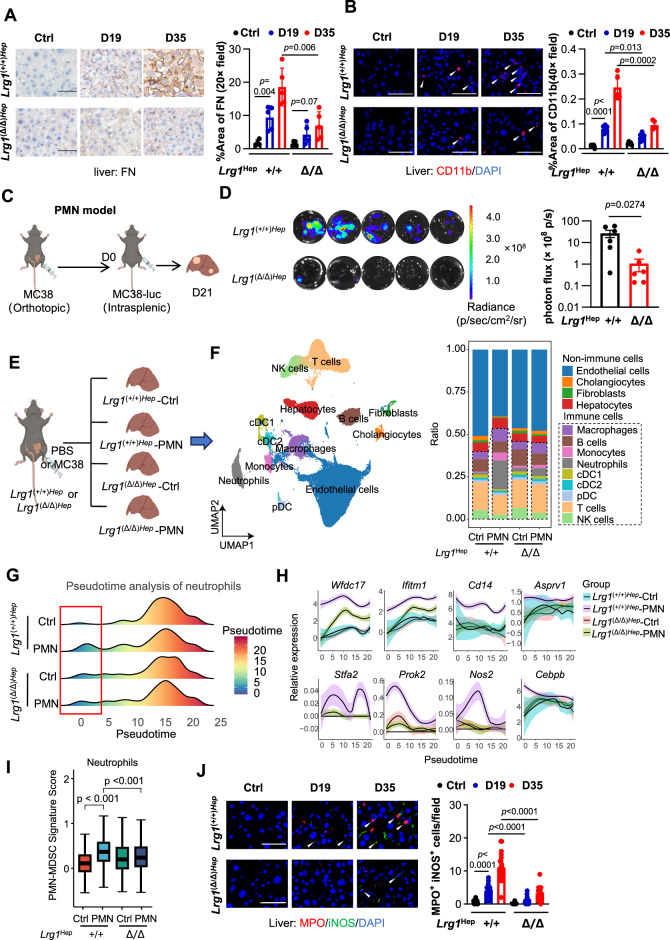
LRG1 promotes the formation of a pre-metastatic niche in the liver. **A** Representative images and quantification of IHC staining for FN in the liver from different groups as indicated in Fig. 2F. *n* = 5 mice per group. Scale bars, 50 μm. Data are means ± SD. **B** Representative images and quantification of immunofluorescence staining for CD11b (arrows indicate CD11b+ cells) in the liver from different groups as indicated in Fig. 2F. *n* = 5 mice per group. Scale bars, 50 μm. Data are means ± SD. **C** Schematic of bioluminescent imaging (BLI) assay in *Lrg1*(+/+)Hep and *Lrg1*(Δ/Δ)Hep mice. **D** Representative bioluminescence images and quantification of liver metastases from different groups. *n* = 6 mice per group. Data are means ± SEM. **E** Schematic of single-cell RNA-seq analysis of liver tissues from *Lrg1*(+/+)Hep and *Lrg1*(Δ/Δ)Hep mice with orthotopic injection of MC38 or PBS. **F** UMAP plot of liver cells from all groups (left), and cell type proportions in different groups (right). Neutrophil pseudotime trajectory: **G** cell density estimates; **H** gene expression dynamics. **I** PMN-MDSC signature score in neutrophils of each group. **J** Representative images and quantification of immunofluorescence staining for MPO (red) and iNOS (green) in the liver from different groups as indicated in Fig. 2F. White arrows indicate MPO+ iNOS+ cells. Dots represent each field of view from all samples. *n* = 5 mice for each group. Scale bars, 50 μm. Data are means ± SEM. *p* values were obtained by two-tailed unpaired Student’s *t* test

Hepatocyte-specific *Lrg1* deletion abolished the establishment of a tumor-induced premetastatic niche, as indicated by the absence of fibronectin deposition and reduced myeloid cell infiltration (Fig. 3A, B). To validate the impact of LRG1-mediated PMN formation on liver metastasis, we induced liver PMNs via orthotopic cecal implantation of MC38 in *Lrg1*^(+/+)Hep^ and *Lrg1*^(Δ/Δ)Hep^ mice. Fourteen days later, MC38-luciferase (MC38-luc) cells were intrasplenically injected to assess liver metastasis (Fig. 3C). Primary tumor-induced PMNs significantly promoted MC38-luc liver metastasis, whereas hepatocyte *Lrg1* KO dramatically reversed this phenomenon (Fig. 3C, D), indicating that LRG1-induced PMNs are essential for liver metastasis.

To determine the effect of hepatocyte-specific *Lrg1* KO on liver PMN formation, we performed single-cell RNA sequencing (scRNA-seq) on liver tissues from healthy and orthotopic MC38-bearing mice (day 19, PMN model) with both *the Lrg1*^(Δ/Δ)Hep^ and *Lrg1*^(+/+)Hep^ genotypes (Fig. 3E). After quality control, 75,053 cells were included for further analysis. Thirteen major cell populations were identified, including immune cells (B cells, DC1, DC2, macrophages, monocytes, neutrophils, NK cells, pDCs, and T cells), hepatocytes, cholangiocytes, endothelial cells, and fibroblasts (Fig. 3F, Fig. S5A) [23].

Immunosuppression is a key feature of PMN [2]. In the premetastatic liver, the proportion of myeloid cells increased, whereas that of lymphoid cells (T/NK and B cells) decreased (Fig. 3F, S5B). The change in the abundance of neutrophils was most dramatic, increasing from 2.89% in healthy *Lrg1*^(+/+)Hep^ mice to 31.68% in *Lrg1*^(+/+)Hep^-PMN mice. This increase was significantly reversed to 10.49% in *Lrg1*^(Δ/Δ)Hep^ mice (Fig. S5B), which was further validated by flow cytometry (Fig. S5C, D). Pseudotime analysis revealed that compared with control mice, *Lrg1*^(+/+)Hep^-PMN livers had a greater proportion of neutrophils at an early developmental stage, which was also diminished upon *Lrg1* deletion (Fig. 3G). These early neutrophils highly expressed genes associated with differentiating bone marrow–derived neutrophils, such as *Camp*, *Ltf*, *Ngp*, and *Chil3* (Fig. S5E) [24, 25]. Since immature neutrophils are often more immunosuppressive, we next analyzed the expression of immunosuppressive genes enriched in myeloid-derived suppressor cells (MDSCs), including *Wfdc17*, *Ifitm1*, *Cd14*, *Prok2*, *Nos2*, *Cebpb*, *Stfa2*, and *Asprv1* [26–33]. These genes were significantly more abundant along the neutrophil pseudotime trajectory in tumor-bearing *Lrg1*^(+/+)Hep^ mice than in their *Lrg1*^(Δ/Δ)Hep^ counterparts (Fig. 3H). MDSCs are pathologically activated neutrophils and monocytes with strong immunosuppressive capacity [34]. An established MDSC signature score [35] was significantly elevated in neutrophils from *Lrg1*^(+/+)Hep^-PMN mice but not in those from *Lrg1*^(Δ/Δ)Hep^-PMN mice (Fig. 3I). Notably, inducible nitric oxide synthase (iNOS, encoded by *Nos2*), which is known to suppress T-cell function [36–39], is highly expressed by immunosuppressive neutrophils to promote metastasis [32, 40]. iNOS^+^ MPO^+^ neutrophil infiltration gradually increased in the liver following metastatic progression in *Lrg1*^(+/+)Hep^ mice, but this trend also decreased in *Lrg1*-KO mice (Fig. 3J). Similarly, the proportion of CD8 + T cells decreased, but that of PD1^+^ CD8 + T cells increased (Fig. S5F, G). Similarly, the MDSC-related immunosuppressive signature in monocytes was elevated in tumor-bearing livers and attenuated by *Lrg1* KO (Fig. S5H). Moreover, the levels of all the dendritic cell (DC1, DC2 and pDC) subsets responsible for antigen presentation were reduced in tumor-bearing *Lrg1*^(+/+)Hep^ mice (Fig. S5B). Tolerogenic DCs promote antigen-specific tolerance by dampening T-cell responses and inducing pathogenic T-cell exhaustion and regulatory T cells [41, 42]. Functional analysis revealed an increase in the expression of tolerogenic DC–related genes in *Lrg1*^(+/+)Hep^-PMN livers, which was reversed by *Lrg1* deletion (Fig. S5I).

Angiogenesis, which is primarily mediated by endothelial cells, is another key feature of PMN formation. Gene Ontology analysis revealed enrichment of angiogenesis- and inflammation-related pathways in endothelial cells from tumor-bearing *Lrg1*^(+/+)Hep^ mice (Fig. S5J). Interestingly, the upregulation of genes involved in angiogenesis (*Hgf*, *Rhob*, *Lrg1*, *Ets1*, and *Il1a*) [22, 43–46] and inflammation (*Il1r1* and *Socs3*) [47, 48] in tumor-bearing mice was attenuated when hepatic *Lrg1* was knocked out (Fig. S5J, K).

Collectively, these findings demonstrate that hepatocyte-derived LRG1 responds to the presence of primary colorectal tumors by reshaping the liver immune microenvironment to support tumor metastasis.

### LRG1 directs NET formation by neutrophils through the TGFBR/PI3K/AKT axis

**Fig. 4:**
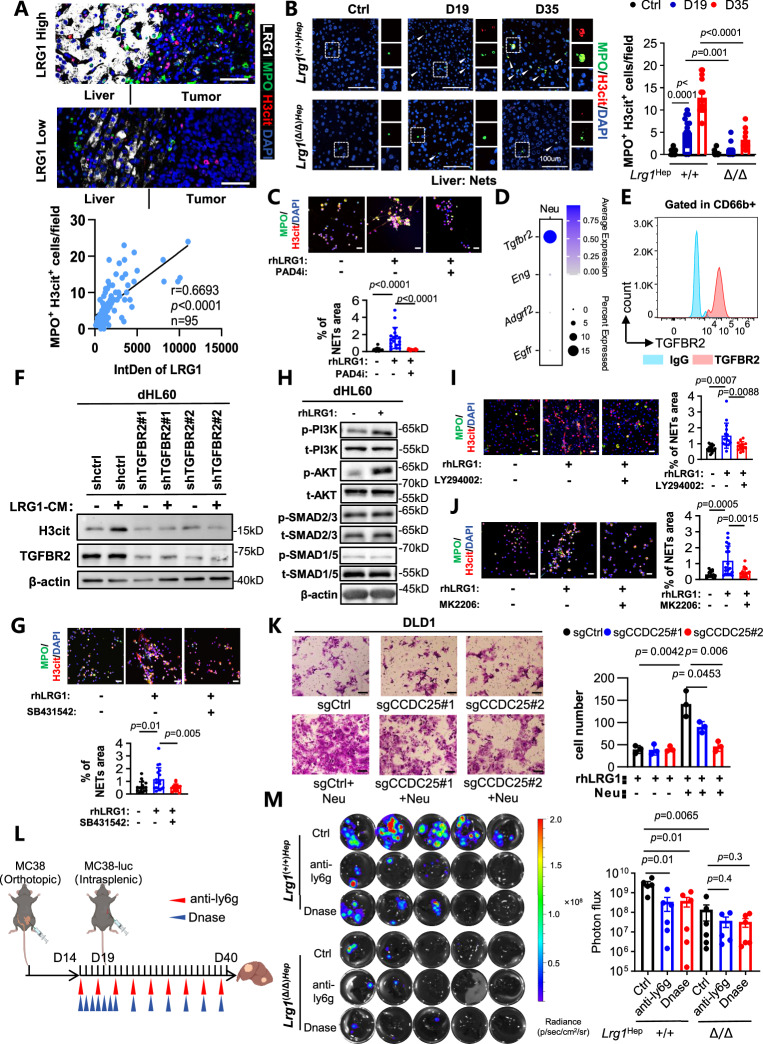
LRG1 promotes liver metastasis by directing NETs formation of neutrophils through TGFBR/PI3K/AKT axis. **A** Representative images of Immunofluorescence staining of LRG1(white), MPO (green) and H3cit (red) in human CRLM samples (*n* = 18). Scale bars, 50 μm. Correlation between the expression of LRG1 and the number of MPO+ H3cit+ cells is shown using Pearson’s correlation analysis. Dots represent field of view (FOV) from all samples. **B** Representative images and quantification of Immunofluorescence staining of MPO (green) and H3cit (red) in liver from *Lrg1*(+/+)Hep and *Lrg1*(Δ/Δ)Hep mice CRC Orthotopic Model as indicated in Fig. 2F. *n* = 5 per group. DAPI is shown in blue. Scale bars, 100 μm. Dots represent field of view (FOV) from all samples. Data are means ± SEM. **C** Immunofluorescence analyses of NETs formed by human neutrophils treated with rhLRG1 and PAD4i as indicated. Scale bars, 100 μm. Dots represent field of view (FOV) from 3 independent experiments. Data are means ± SD. **D** scRNA-seq analysis expression of *Egfr*, *Adgrl2*, *Eng* and*Tgfbr2* in neutrophils. **E** Flow cytometry analysis of TGFBR2 expression in human neutrophils. **F** Western blot detected expression of H3cit, TGFBR2 and β-actin in dHL60-shTGFBR2 cells treated with or without LRG1- conditioned medium(CM). **G** Immunofluorescence analyses of NETs formed by human neutrophils treated with rhLRG1 and SB431542. Scale bars, 100 μm. Dots represent field of view (FOV) from 3 independent experiments. Data are means ± SD. **H** Western blot analysis showing the expression of H3cit, p-AKT, t-AKT, p-SMAD2/3, t-SMAD2/3, p-SMAD1/5, t-SMAD1/5, p-PI3K, t-PI3K and β-actin in dHL60 treated with or without rhLRG1. **I** Immunofluorescence analyses of NETs formed by human neutrophils treated with rhLRG1 and LY294002. Scale bars, 100 μm. Dots represent field of view (FOV) from 3 independent experiments. Data are means ± SD. **J** Immunofluorescence analyses of NETs formed by human neutrophils treated with rhLRG1 and MK2206. Scale bars, 100 μm. Dots represent field of view (FOV) from 3 independent experiments. Data are means ± SD. **K** Transwell migration assays for DLD-1-sgctrl or sgCCDC25 cells treated with rhLRG1 and/or neutrophils. *n* = 3 independent experiments. Scale bars, 50 μm. Data are means ± SD. As depicted in the schematic **L**, liver metastases were determined in *Lrg1*(+/+)Hep and *Lrg1*(Δ/Δ)Hep mice treated with or without anti-Ly6G and Dnase. Shown are representative images and BLI analyses of each group **M**. *n* = 5-6 and data are means ± SEM. Statistical significance was determined using two-tailed unpaired Student’s *t* test **B, C, G, K, and M**

Single-cell RNA-seq analysis comparing neutrophils from the premetastatic liver microenvironment with control neutrophils revealed that, during niche formation, pathways related to the pathogen response and chemotaxis were highly upregulated in neutrophils, indicating an inflammatory-activated state (Fig. S6A). Consistent with these findings, RNA sequencing of bulk liver tissue demonstrated enrichment of neutrophil extracellular trap formation prior to metastasis (Fig. S6B, S6C). NET formation is closely linked to tumor colonization and metastatic seeding [49–51]. We then examined human liver metastasis specimens and found that LRG1 expression in the liver correlated positively with NETosis markers (Fig. 4A). Furthermore, single-cell data comparing *Lrg1*^(+/+)Hep^ and *Lrg1*^(Δ/Δ)Hep^ mice revealed that the deletion of *Lrg1* substantially attenuated the upregulation of inflammatory genes in neutrophils during the establishment of the premetastatic niche (Fig. S6D), demonstrating that liver-derived LRG1 is essential for the activation of neutrophil inflammation. In both orthotopic and splenic injection mouse models of liver metastasis, *Lrg1*^(+/+)Hep^ mice accumulated NETs progressively, whereas *Lrg1* knockout almost completely abolished NET deposition in the liver (Fig. 4B; Fig. S6E). Similarly, *Lrg1* deletion prevented the increase in circulating NETs during metastatic progression (Fig. S6F).

To assess whether LRG1 directly acts on neutrophils, we treated primary human neutrophils with recombinant LRG1. LRG1 expression significantly increased neutrophil chemotaxis (Fig. S6G) and induced NETosis—a process reversed by an inhibitor of PAD4 (a key enzyme in NET formation) (Fig. 4C). Similarly, LRG1 induced NETosis in differentiated HL60 cells (neutrophil-like) (Fig. S6H), and mouse hepatocytes overexpressing LRG1 triggered NET formation in cocultured mouse neutrophils (Fig. S6I, S6J). Reported LRG1 receptors include TGFBRⅡ [22], endoglin [22], ADGRL2 [52], and EGFR [14, 53]. Single-cell expression profiling of neutrophils revealed that only *Tgfbr2* was appreciably expressed (Fig. 4D), and its expression was validated by FACS (Fig. 4E), suggesting that LRG1-induced NETosis might be mediated by TGFBRⅡ. Indeed, genetic ablation of *TGFBR2 or TGFBR1* suppressed LRG1-driven NETosis (Fig. 4F,S6K). Moreover, the TGFBRⅡ inhibitor SB431542 also significantly blocked LRG1-induced NET formation in both donor neutrophils and dHL60 cells (Fig. 4G and Fig. S6L). To further identify signaling downstream of LRG1-TGFBR, we examined both canonical SMAD-dependent signaling and noncanonical PI3K–AKT signaling downstream of TGFβ receptors. Our results revealed that LRG1 selectively activated the noncanonical PI3K–AKT pathway in neutrophils (Fig. 4H), whereas SMAD and other reported signaling pathways involved in NETosis were not detected (Fig. 4H, Fig. S6M). These findings suggest that LRG1 regulates NET formation specifically through a TGFBR–PI3K–AKT signaling axis rather than through the canonical SMAD pathway. Consistently, pharmacological inhibition of PI3K with LY294002 or inhibition of AKT with MK2206 effectively suppressed LRG1-induced NET formation in neutrophils (Fig. 4I, J, and Fig. S6N, S6O). Together, these data demonstrate that LRG1 directs NET formation through TGFβ receptor–mediated activation of the PI3K–AKT pathway.

### LRG1-induced NETosis is responsible for liver metastasis

NETosis has been shown to facilitate tumor cell adhesion and migration. To test whether LRG1-induced NETs enhance tumor cell motility, we performed Transwell assays with colorectal cancer cell lines. Coculture with neutrophils supplemented with LRG1 markedly increased the migration of DLD1 and HCT116 cells, an effect that was reversed by treatment with DNase to degrade NET DNA (Fig. S7A–S7C). Moreover, the expression of CCDC25, a receptor on tumor cells that binds extracellular DNA [51], was required for this enhanced migration, as knocking out CCDC25 in DLD1 and HCT116 cells dramatically reduced their movement under coculture conditions (Fig. S7D–S7G; Fig. 4K).

In vivo, *Lrg1*^(+/+)Hep^ and *Lrg1*^(Δ/Δ)Hep^ mice were first implanted with orthotopic MC38 cells to establish a premetastatic niche and then injected intrasplenically with luciferase-tagged MC38-Luc cells. Prior to injection, the mice were assigned to the control, anti-Ly6G antibody (neutrophil depletion), or DNase (NET depletion) groups. Longitudinal bioluminescence imaging revealed that *Lrg1*^(+/+)Hep^ controls developed the greatest liver metastatic burden, while both neutrophil depletion and NET degradation reduced metastasis; moreover, compared with control mice, LRG1-deficient mice exhibited minimal liver metastases across all conditions (Figs. 4L, M; Fig. S7H). These results confirm that LRG1-driven liver metastasis depends on its ability to induce NETosis.

### Tumor-associated inflammation promotes the expression of LRG1 in hepatocytes via the IL6/STAT3 signaling cascade

**Fig. 5:**
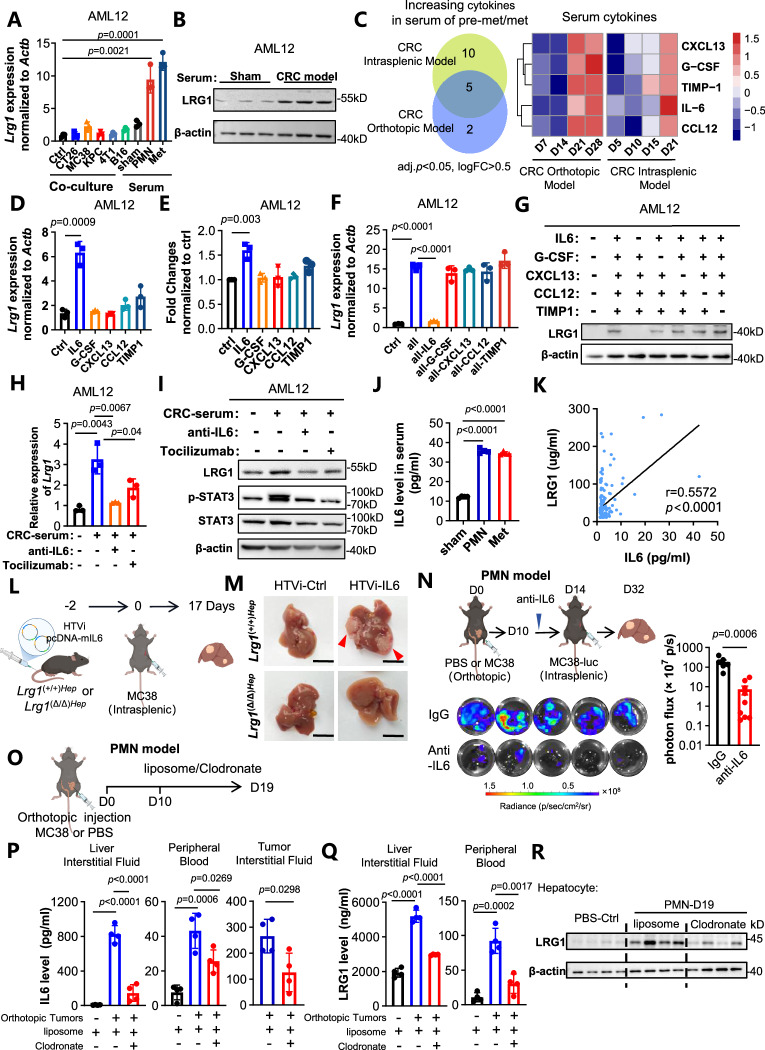
Tumor-associated inflammation promotes the expression of LRG1 in hepatocytes by IL6/STAT3. **A** The relative expression of *Lrg1* in AML12 cells co-cultured with various mouse tumor cell lines or treated with serum (5%) from CRC model mice. *n* = 3 independent experiments. Data are means ± SD. **B** Western blot analysis of the expression LRG1 and β-actin in AML12 cells after the indicated treatments. **C** Cytokine array analyses of serum from CRC orthotopic mice model (day 7, day 14, day 21 and day 28) and from CRC intrasplenic mice model (day 5, day 10, day 15 and day 21). Quantitative real-time PCR analyses of the expression *Lrg1* **D** and ELISA analyses of media of AML12 **E** treated with vehicle or recombinant IL6/G-CSF/CXCL13/CCL12/TIMP1. *n* = 3 independent experiments. Data are means ± SD. **F, G** qRT-PCR and Western blot analyses of LRG1 in AML12 cells treated with cytokine combinations. *n* = 3 independent experiments. Data are means ± SD. **H, I** qRT-PCR and western blot analysis of the expression LRG1 and β-actin in AML12 cells treated with indicated serum and anti-IL6 or tocilizumab. *n* = 3 independent experiments. Data are means ± SD. **J** ELISA analyses of serum samples for IL6 from sham-group, CRC orthotopic model at day 21(PMN) and CRC intrasplenic model at day 21(Met) in BALB/c. *n* = 4. Data are means ± SD. **K** Correlation between serological IL6 and LRG1 levels of CRC patients is shown using Pearson’s correlation analysis. Dots represent individual samples. *n* = 161. **L, M** Schematic of the experimental design **L**. Representative images of liver metastases **M** in each group (*n* = 6 in *Lrg1*(+/+)Hep-HTVi-Ctrl group and *Lrg1*(+/+)Hep-HTVi-IL6 group, *n* = 5 in *Lrg1*(Δ/Δ)Hep-HTVi-Ctrl group and *Lrg1*(Δ/Δ)Hep-HTVi-IL6 group). Scale bars, 1 cm. **N** Schematic of the experimental design. Representative bioluminescence images and analyses of liver metastases in CRC mouse model treated with anti-IL6 or IgG. **O** Schematic of the experimental design. **P** ELISA analyses of samples for IL6 levels from liver interstitial fluid, peripheral blood and tumor interstitial fluid of mice from different groups as indicated. *n* = 4. Data are means ± SD. **Q** ELISA analyses of samples for LRG1 levels from liver interstitial fluid and peripheral blood of mice from different groups as indicated. *n* = 4. Data are means ± SD. **R** Western blot analyses of LRG1 and β-actin in hepatocyte of mice from different groups as indicated. Statistical significance was determined using two-tailed unpaired Student’s *t* test **A, D–F, H, and N–P**

Tumor-derived secreted factors play pivotal roles in shaping the premetastatic niche [3, 4]. To determine how LRG1 was upregulated in the context of tumor burden, we cocultured mouse AML12 hepatocytes with various tumor cell lines (CT26, MC38, KPC, 4T1, and B16F10) or treated them with serum from premetastatic or metastatic mice. Only serum from premetastatic and metastatic mice significantly increased *Lrg1* mRNA and protein levels in AML12 cells (Fig. 5A, B), indicating that tumor-associated systemic changes, rather than tumor cell-derived factors, drive LRG1 induction.

Cytokine array analysis of mouse serum from orthotopic and splenic models revealed 5 commonly elevated cytokines during metastasis, namely, IL6, G-CSF, CXCL13, CCL12, and TIMP1 (Fig. 5C; Fig. S8A). Among these genes, only IL6 robustly upregulated hepatocyte *Lrg1* transcription and secretion when it was added individually to AML12 (Fig. 5D, E) or mouse hepatocyte cultures (Fig. S8B, S8C). Combinatorial cytokine experiments confirmed that IL6 was indispensable for LRG1 induction, whereas the removal of other factors had a minimal effect (Fig. 5F, G). Furthermore, the neutralization of IL6 with an antibody or the blockade of IL6R with tocilizumab abrogated the upregulation of LRG1 expression (Fig. 5H, I). In vivo, serum IL6 levels (Fig. 5J) and hepatic phospho-STAT3 levels (Tyr705) (Fig. S8D, S8E) increased during metastatic progression. Analysis of serum from patients with colorectal cancer revealed a positive correlation between IL6 and LRG1 levels (Fig. 5K). Finally, hydrodynamic tail vein overexpression of *Il6* followed by splenic MC38 injection resulted in high levels of LRG1 and metastatic niche formation in the liver (Fig. S8F–H) and strongly promoted liver metastasis (Fig. 5L, M; Fig. S8I–S8J), an effect that was significantly blunted in the absence of hepatocyte *Lrg1* in *Lrg1*^(Δ/Δ)Hep^ mice (Fig. 5L, M; Fig. S8I–S8J). Consistently, in vivo administration of an anti-IL-6 antibody during premetastatic niche formation greatly suppressed hepatic LRG1 induction (Fig. S8K, S8L) and reduced liver metastasis (Fig. 5N, and Fig. S8M).

To identify the cellular source of IL6, we reanalyzed single-cell datasets from normal liver, premetastatic liver, and primary tumors [54]. Although several cell types expressed *Il6*, including tumor cells [55], cancer-associated fibroblasts (CAFs) [56, 57], macrophages [58], and endothelial cells [59–62], the results revealed that only macrophage Il6 expression was consistently and markedly upregulated concomitant with premetastatic niche formation in the liver (Fig. S9A–S9C), which was validated by immunofluorescence staining (Fig. S9D–S9E). To determine whether macrophages are the main contributors to IL6, we depleted macrophages using clodronate liposomes in our premetastatic niche model (Fig. S9F–S9G) [63]. Macrophage depletion in tumor-bearing mice led to a dramatic reduction in IL-6 levels in the liver interstitial fluid (Fig. 5O), where its induction was most pronounced, as well as in the primary tumor and serum (Fig. 5O). Concurrently, the LRG1 levels decreased (Fig. 5P, Q). Collectively, these data establish that tumor-educated macrophages in the premetastatic liver might serve as a major source of IL-6 in this context.

### Targeting LRG1 diminishes liver metastasis and promotes ICB efficacy in metastatic tumors

**Fig. 6:**
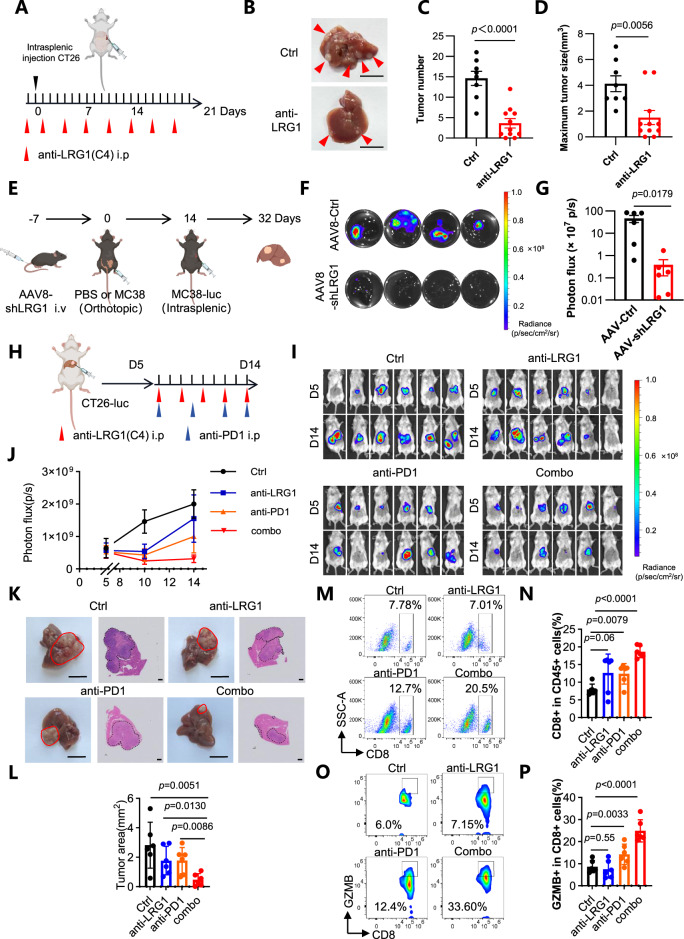
Targeting LRG1 reduces colorectal cancer liver metastasis and enhances the efficacy of immunotherapy for metastatic liver tumors. **A** Schematic of CRC intrasplenic model treated with anti-LRG1 antibody. Representative images of liver metastases **B** and quantification of the number. **C** and maximum tumor size **D** of liver metastases in control (*n* = 8) and anti-LRG1-treated (*n* = 11) mice. Red arrows indicate liver metastases. Scale bars, 1 cm. Data are means ± SEM. As depicted in the schematic **E**, liver metastases in mice were measured by ex vivo bioluminescent imaging (BLI). Shown are representative images of each group (right) **F**. Shown are BLI analyses **G**. *n* = 6. Data are means ± SEM. Schematic of CRCLM hepatic model treated with or without anti-LRG1 and anti-PD1 **H**. Tumors in liver were measured by ex vivo bioluminescent imaging (BLI). Shown are images of each group **I** and BLI analyses **J**. *n* = 6. Representative images of tumors in the liver and H&E staining of tumor burden in liver **K**. Scale bars, 1 cm. Scale bars, 10 μm. Quantification of tumor area in H&E staining of tumor burden in liver **L**. *n* = 6. Data are means ± SD. Flow cytometry analyses of the percentages of CD8+ cells in CD45+ immunocytes **M, N** and the percentages of GZMB+ cells in CD8+ cells **O, P** in the tumors. *n* = 6. Data are means ± SD. *p* values were obtained by two-tailed unpaired Student’s *t* test

To evaluate LRG1 as a therapeutic target, we administered an anti-LRG1 antibody to mice that received a splenic CT26 injection (Fig. 6A). Compared with control treatment, antibody treatment effectively abolished the increase in LRG1 expression (Fig. S10A), prevented NET formation in the liver (Fig. S10B, S10C) and significantly reduced both the number and size of liver metastases (Fig. 6B–D). Similarly, AAV8-mediated, hepatocyte-specific *Lrg1* knockout prior to metastasis decreased the metastatic burden (Fig. 6E–G) and orthotopic tumor size (Fig. S10D).

Since liver metastases often confer resistance to immune checkpoint blockade [64, 65], we tested whether LRG1 inhibition could sensitize tumors to anti-PD-1 therapy (Fig. 6H). In a model of direct intrahepatic implantation of CT26 cells, combined treatment with anti-LRG1 and anti-PD-1 antibodies synergistically suppressed tumor growth (Fig. 6I, J) and liver tumor burden (Fig. 6K–L). Flow cytometry revealed that dual blockade markedly increased CD8⁺ T-cell infiltration (Fig. 6M, N) and the proportion of cytotoxic T cells within metastatic lesions (Fig. 6O).

## Discussion

**Fig. 7:**
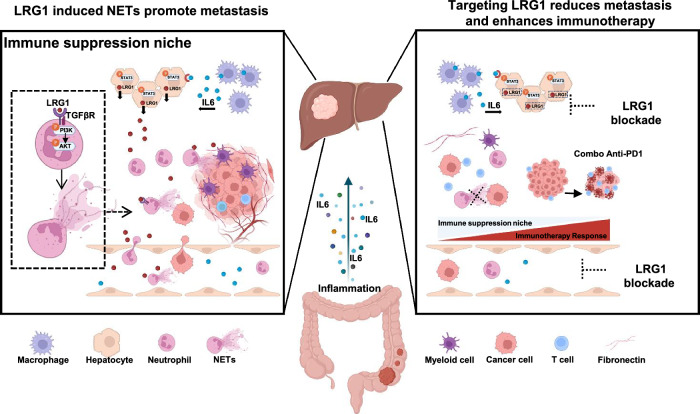
The schematic model illustrating the mechanisms of Hepatocyte-derived LRG1 promote liver PMN formation and targeting approach. The tumor-associated inflammatory response promotes hepatic macrophage infiltration and subsequent IL-6 secretion, which induces hepatocyte-derived LRG1 production and facilitates pre-metastatic niche formation in the liver. This process includes the recruitment of immunosuppressive myeloid cells—predominantly neutrophils—and the induction of NETs formation via TGFβ receptor signaling. These mechanisms facilitate tumor colonization and create an immunosuppressive hepatic environment. Therapeutic targeting of hepatocyte-derived LRG1 or the use of neutralizing antibodies may attenuate this process and offer a promising clinical strategy

Our study revealed that hepatocyte-derived LRG1 is a central systemic mediator that links primary tumors to the liver premetastatic niche. Clinically, elevated serum LRG1 levels strongly correlate with both existing and future liver metastases across several gastrointestinal malignancies, and high baseline LRG1 levels predict poor liver metastasis-free survival. Importantly, hepatocyte-derived LRG1 was both necessary and sufficient for promoting liver PMN formation. These findings extend the recognized role of LRG1 beyond local effects on tumors or the endothelium, demonstrating that liver parenchymal cells can be coopted by distant tumors to create a metastasis-permissive microenvironment (Fig. 7).

Our data position LRG1 squarely in the emerging paradigm of tumor–host crosstalk. LRG1 was first identified as a promoter of pathological angiogenesis via the modulation of endothelial TGFβ signaling and has since been implicated in tumor EMT, tumor growth, and tumor metastasis. Notably, a recent report demonstrated that liver-secreted LRG1 activates HER3 to sustain metastatic colorectal tumors in the liver, highlighting LRG1 as a liver-to-tumor growth signal. While previous studies have reported that IL-6/STAT3 signaling regulates LRG1 in tumor cells, our findings demonstrate that the IL-6/LRG1 cascade is linked to systemic inflammation—a common feature of advancing tumor-to-hepatic PMN establishment. Our work also provides complementary insight: in addition to tumor-intrinsic LRG1 expression, hepatocyte-released LRG1 profoundly remodels the liver immune landscape to favor metastasis. In this context, our data suggest that liver-infiltrating macrophages represent an important intermediary cellular component, as they appear to be a major source of IL-6 in the premetastatic liver. Given the well-established role of macrophages in shaping the tumor microenvironment, further investigation into macrophage recruitment and functional heterogeneity within the premetastatic niche will be important to elucidate this process more fully.

We show that LRG1 recruits and reprograms myeloid cells in the liver. Strikingly, we found that LRG1 directly induces neutrophil extracellular trap (NET) formation. NETosis has emerged as a key enabler of metastasis through the trapping of tumor cells and fostering tumor growth. This is the first demonstration that a hepatocyte-derived factor can orchestrate neutrophil NETs during PMN formation. Interestingly, previous studies have shown that LRG1 is itself secreted by activated neutrophils, suggesting that a feed-forward loop in which LRG1 is released from neutrophils may further amplify local niche effects.

The immunological consequences of LRG1 in the PMN were profound. LRG1-rich premetastatic livers harbor large infiltrates of immature neutrophils and inflammatory monocytes with a suppressive MDSC-like gene signature, while T-cell populations contract and become more exhausted. Whether LRG1 directly affects other immune cells (such as T cells and dendritic cells) remains to be determined, and our data clearly reveal that LRG1 drives an immunosuppressive microenvironment.

From a translational perspective, our data suggest significant implications. First, elevated LRG1 expression predicted future metastasis in multiple GI cancers in our cohorts, which is akin to reports that high LRG1 expression portends poor outcomes in PDAC and other cancers, underscoring its potential as an early, noninvasive biomarker. Integrating LRG1 into diagnostic panels may improve the early detection of occult metastasis. Second, LRG1 itself is a promising therapeutic target. Here, anti-LRG1 antibody treatment prevented NET formation and dramatically reduced liver metastasis. Importantly, combining LRG1 blockade with PD-1 checkpoint inhibitors had a synergistic effect, unleashing cytotoxic T cells in liver tumors. This is particularly relevant since liver metastases are notoriously resistant to immunotherapy [64, 65]. Recent evidence has shown that LRG1 inhibition can enhance the efficacy of chemotherapy and checkpoint blockade by normalizing the tumor vasculature [9]. Our data suggest that targeting LRG1 can reprogram the metastatic niche from “cold” to “hot,” increasing its susceptibility to ICB.

## Materials and methods

### Patients and specimens

Serum samples from patients were obtained from BioBank, The Six Affiliated Hospital, Sun Yat-sen University. Paraffin-embedded human liver metastasis sections were obtained from the Sixth Affiliated Hospital of Sun Yat-sen University. The samples were used with informed consent under a protocol approved by the Medical Ethics Committee of the Sixth Affiliated Hospital of Sun Yat-sen University.

### Animal experiments

*Lrg1*-flox mice (strain no. T009577) and *Alb-Cre* mice (strain no. T017784) on a C57BL6/J background were purchased from GemPharmatech (Nanjing, China). *Lrg1*^wt/wt^; *Alb-cre* (termed *Lrg1*^(+/+)Hep^), *Lrg1*^wt/fl^; *Alb-cre* (termed *Lrg1*^(+/∆)Hep^), and *Lrg1*^fl/fl^; *Alb-cre* (termed *Lrg1*^(∆/∆)Hep^) were generated by crossing *Lrg1*-*flox* mice and *Alb-Cre* mice. Genotyping of mice was performed by polymerase chain reaction (PCR) analysis of genomic DNA extracted from mouse tails using primers (Table S1).

For all tumor models, BALB/c or C57BL/6 J mice between 6 and 10 weeks of age were purchased from Guangdong GemPharmatech unless otherwise indicated. Mice of similar sex, age and weight were randomized before tumor inoculation. To determine the primary source of LRG1 in tumor models, mice were orthotopically implanted with 1×10^6^ CT26 cells or MC38 cells, 5×10^4^ KPC cells, or 1×10^6^ B16F10 cells and intrasplenically implanted with 5×10^4^ CT26 cells. The mice were euthanized at the indicated time points, and serum, specific organs and cells were collected to detect the expression of LRG1.

To detect liver metastases in *Lrg1*^(+/+)Hep^, *Lrg1*^(+/∆)Hep^ and *Lrg1*^(∆/∆)Hep^ mice, mice were implanted with 1×10^5^ MC38 cells and euthanized. To determine the effect of PMN on liver metastases in *Lrg1*^(+/+)Hep^ and *Lrg1*^(∆/∆)Hep^ mice, mice were first orthotopically implanted with 1×10^6^ MC38 cells. Afterward, the luciferase-labeled MC38-luc cells were intrasplenically implanted (1×10^5^ cells for each mouse). The mice were euthanized, and the liver was rapidly harvested for ex vivo BLI.

To determine the effects of neutrophil depletion and DNase I on liver metastases in *Lrg1*^(∆/∆)Hep^ mice, the mice were orthotopically implanted with 1×10^6^ MC38 cells. On day 14, the mice received an initial i.p. injection of anti-Ly6G antibody (200 μg/mouse; Bio Xcell, #BE0075) every 3 days and an i.p. injection of DNase I (5 mg/kg, daily shots for 7 days from day 14, and then maintenance shots every 3 days). On day 40, the mice were euthanized, and the livers were rapidly harvested for ex vivo BLI. To test whether macrophages are the main contributors to IL6, mice were orthotopically implanted with 1×10^6^ MC38 cells. On day 10, the mice received initial i.p. injections of clodronate liposomes (200 μl/mouse; Yeasen Biotechnology, 40337ES08) or liposomes (200 μl/mouse; Yeasen Biotechnology, 40338ES08) every 3 days and were euthanized on day 19 for further experiments.

To determine the therapeutic efficacy of anti-IL6 antibody in the CRCLM model, mice were i.p. injected with anti-IL6 antibody (200 μg/mouse; Selleck, A2118) or IgG antibody on day 10 after orthotopic injection, followed by every 3 days of injection. On day 14, MC38-luc cells were intrasplenically implanted; on day 32, the mice were euthanized, and the livers were rapidly harvested for ex vivo BLI. To determine the therapeutic efficacy of anti-LRG1 (C4, sc-390920) in a CRC intrasplenic model, mice were i.p. injected with anti-LRG1 (2 µg/mouse) 1 day before intrasplenic injection of cancer cells, followed by every 3 days of injection of the reagents for 21 days. On day 21, the mice were euthanized to detect liver metastases. To determine the therapeutic efficacy of AAV8-TGB-shLRG1 (5’-TGTCCATCTGTCGGTGGAATT-3’) in the CRCLM model, mice were i.v. injected with AAV-TGB-shLRG1 1 day before the orthotopic injection of MC38. On day 14, MC38-luc cells were intrasplenically implanted; on day 32, the mice were euthanized, and the livers were rapidly harvested for ex vivo BLI.

To determine the combined effect of anti-LRG1 and anti-PD-1 immunotherapy, BALB/c mice were used, and 2.5×10^5^ CT26-luc cells in 25 μl of PBS were injected into the left main lobe of the mouse liver. On day 5, the mice were grouped according to ex vivo BLI and received an initial i.p. injection of anti-LRG1 every 2 days and anti-PD-1 (100 μg/mouse) every 3 days until completion of the experiment.

The mice were given a standard diet, allowed free access to water, and housed under a 12-h light/dark cycle. All the animal experiments were approved by the Institutional Animal Care and Use Committee of Sun Yat-sen University and conformed to the Guide for the Care and Use of Laboratory Animals of the National Institutes of Health (National Academies Press, 2011) in China.

### Ex vivo bioluminescence imaging (BLI)

For the liver metastasis models used in the present study, luciferase activity in the liver was used to monitor liver metastasis progression. The mice were i.p. injected with 100 μl of D-luciferin (150 mg/kg) and anesthetized for ex vivo BLI. The mice were euthanized, and the livers were rapidly dissected and placed in a plate filled with 2 ml of D-luciferin (150 mg/ml; diluted in PBS) for ex vivo BLI. BLI results were obtained using the Xenogen IVIS system. Light emission from the region of interest was quantified as photons/second/cm2/steradian (p/sec/cm2/sr) through Living Images software.

### HTVi animal experiment

All these experimental procedures were approved by the Institutional Animal Care and Use Committee of Sun Yat-sen University. To overexpress LRG1 or IL6 in the liver, we hydrodynamically injected plasmid DNAs into the tail vein of mice following a previously published protocol. Each mouse received 10% of its body weight of saline containing 25–50 μg of plasmid DNA (pcDNA3.4-mLRG1 or pcDNA3.4-mIL6). The mice were maintained on a standard diet and sacrificed at the indicated time points.

### Cell culture

CT26 colon adenocarcinoma cells and B16F10 melanoma cells were obtained from the American Type Culture Collection. MC38 colon adenocarcinoma cells were purchased from Kerafast. The murine pancreatic tumor KPC cell line was derived from the pancreatic tumors of KrasG12D/+; Trp53R172H/+; Pdx1-Cre C57BL/6 mice. The human CRC cell lines DLD1 and HCT116 were obtained from the American Type Culture Collection. The murine hepatocyte AML12 cell line and HL60 cell line were obtained from the American Type Culture Collection. CT26 cells were cultured in RPMI-1640 media supplemented with 10% fetal bovine serum and penicillin/streptomycin. MC38, KPC, DLD1 and HCT116 cells were cultured in DMEM supplemented with 10% fetal bovine serum and penicillin/streptomycin. AML12 cells were cultured in DMEM/F12 supplemented with 10% fetal bovine serum, 1× ITS (Sigma‒Aldrich), 40 ng/ml dexamethasone (Sigma‒Aldrich) and penicillin/streptomycin. HL60 cells were cultured in IMDM supplemented with 10% fetal bovine serum and penicillin/streptomycin. To differentiate HL60 cells into neutrophil-like cells, the cells were incubated in culture medium supplemented with 1% DMSO for 7 days. All the cells were cultured in a humidified incubator at 37 °C with 5% CO2.

### RNA extraction and quantitative real-time PCR (qRT‒PCR)

Total RNA was extracted from cells and tissues using TRIzol reagent (Invitrogen) and ethanol precipitation. RNA reverse transcription was performed using a KAPA SYBR® FAST Universal kit. qPCR was subsequently conducted using a Roche Light-Cycler 480. The primers used are listed in Table S1.

### Plasmid construction and lentiviral vector transduction

To generate the LRG1 overexpression plasmids, the full-length cDNA of mouse LRG1 was cloned and inserted into the pLenti-CMV vector. sgRNAs targeting CCDC25 were synthesized and cloned and inserted into Lenti-CRISPR-V2-Puro plasmids. shRNAs targeting TGFBR2 and TGFBR1 were synthesized and cloned and inserted into PLKO.1 plasmids. For stable transfection, the above constructs were cotransfected with pMD2. G, pRSV-REV and pMDLg/pRRE packaging plasmids were added to HEK293T cells in accordance with the manufacturer’s protocols. Lentiviral vector-containing supernatants were collected and used to knock out CCDC25 in DLD1 and HCT116 cells. The primers used are listed in Table S2.

### Western blot

Cells and tissues were lysed in radioimmunoprecipitation assay (RIPA) buffer (Sigma‒Aldrich) supplemented with protease inhibitor (Roche) and quantified using a BCA kit (Thermo Scientific). The proteins were separated by SDS‒PAGE and transferred to NC membranes. The membranes were subsequently blocked with milk, followed by incubation with specific primary antibodies against LRG1, H3cit, p-AKT, t-AKT, p-ERK, t-ERK, p-p38, p38, p-SMAD2/3, t-SMAD2/3, p-SMAD1/5, t-SMAD1/5, p-PI3K, PI3K, TGFBR2, TGFBR1, CCDC25 and β-actin overnight at 4 °C. The details of the antibodies used are listed in Table S3. Afterward, the membranes were washed with TBST and incubated with HRP-conjugated secondary antibodies (1:5000; Sigma). The bands were visualized by chemiluminescence using the StarSignal Western ECL Substrate (GeneStar).

### Immunofluorescence

The tissue was first fixed in 4% paraformaldehyde, embedded in paraffin and sectioned at a thickness of 4 μm. Then, the paraffin-embedded tissue sections were deparaffinized, rehydrated and subjected to antigen retrieval in EDTA buffer in a microwave. The sections were blocked with 5% BSA for 30 min at room temperature. The samples were subsequently incubated with specific primary antibodies against CD11b, LRG1, MPO, iNOS, and H3cit overnight at 4 °C. After rinsing with PBS, fluorochrome-conjugated secondary antibodies were added and incubated for 1 h at room temperature. The slides were counterstained with DAPI (D1306; Invitrogen). The details of the antibodies used are listed in Table S3. Observation and imaging were performed with a confocal microscope (Cell Observer; ZEISS, Germany). The analysis of fluorescence images relies primarily on ImageJ.

### IHC staining

Paraffin-embedded tissue sections were deparaffinized with dimethylbenzene, dehydrated in an ethanol gradient, and subjected to antigen retrieval with EDTA buffer. Next, the tissues were blocked with normal goat serum and incubated with specific primary antibodies against fibronectin, LRG1 and p-Stat3 overnight at 4 °C. The tissue sections were then incubated with secondary antibodies for 1 h, and positive staining was visualized with an HRP DAB substrate kit, and the nuclei were counterstained with hematoxylin. The expression of LRG1 was quantified on the basis of the intensity of staining and the percentage of positive cells. In brief, the proportion of positive cells was estimated and given a score ranging from 1 to 4 (1, less than 5%; 2, 5–25%; 3, 26–50%; 4, > 51%). The average intensity of the positively stained cells was also given a score on a scale from 1 to 4 (1, no staining; 2, weak staining; 3, moderate staining; 4, strong staining). The final IHC score of each tissue sample was then calculated by multiplying the positive percentage score by the intensity score.

For LRG1, MPO, H3cit, IL6, F4/80, a-SMA, CD31, panCK, CD8a, and GZMb, tissue staining was performed with TSA (tyramide signal amplification) according to the manufacturer’s instructions. The slides were counterstained with DAPI (D1306; Invitrogen). The details of the antibodies used are listed in Table S3.

### Tissue interstitial fluid collection

Tissue interstitial fluid from normal liver, PMN-liver and tumor tissue was isolated. Briefly, the tissues were chopped into pieces, and the tissue pieces were placed on a 70 µM filter on top of a 50 ml conical tube, followed by centrifugation at 200 × g for 10 min. The flowthrough tissue interstitial fluid was flash-frozen at -80 °C.

### ELISA

ELISA kits were used to measure the levels of human LRG1 (Ray Bio), human IL6 (Boster, EK0410), mouse LRG1 (ELK Biotechnology), and mouse IL6 (Boster, EK0411) in cell culture supernatants or serum samples according to the manufacturer’s instructions. We detected plasma MPO-DNA using a previously described sandwich ELISA method. Briefly, 96-well microtiter plates were coated with 5 µg/ml anti-MPO monoclonal antibody (R&D, AF3667) as the capture antibody overnight at 4 °C. After blocking with 1% BSA buffer for 1 h, 50 µl of sample was added per well and incubated for 2 h at room temperature. A Quant-iTTM PicoGreenTM dsDNA Reagent (Thermo Fisher, P7589) and a kit were used to measure the levels of MPO-DNA following the manufacturer’s instructions. The absorbance was measured using a microplate reader.

### Tissue dissociation

For flow cytometry of liver cells, single-cell suspensions were prepared from freshly excised mouse livers by mechanical trituration, after which the samples were passed through 70 mm steel mesh, and hepatocytes were isolated from the cell suspensions by centrifugation at 50 g for 5 min. The remaining cells were used for flow cytometry. For flow sorting of liver cells, livers were extracted and minced, and hepatocytes were isolated by mechanical trituration and centrifugation. The remaining liver tissue was digested with collagenase II (1 mg/ml) and DNase (100 µg/ml) in DMEM for 20 min at 37 °C. The cells were then filtered through 70-mm strainers to remove small fragments of undigested tissue for subsequent experiments.

### Flow cytometry and sorting

Single-cell suspensions from mouse tissues were first stained with anti-mouse CD16/32 (BioLegend, #101320) to block the IgG Fc receptor, after which the cells were stained with surface fluorescent antibodies on ice for 30 min. The details of the antibodies used are listed in Table S3. Flow cytometry was performed on a Beckman CytoFLEX flow cytometer.

### Isolation and culture of primary mouse hepatocytes

Mouse primary hepatocytes were isolated by liver perfusion medium using a 2-step retrograde procedure. Under terminal anesthesia, the mice underwent laparotomy, the inferior vena cava was then cannulated, and the superior vena cava was clamped to achieve retroperfusion of the liver using the portal vein as an outlet. The liver was perfused sequentially with buffer A (HBSS + 0.2 mg/ml EDTA + 1 mg/ml glucose) and then buffer B (HBSS + 0.75 mg/ml collagenase IV + 0.02 mg/ml DNase + 1 mg/ml glucose). After perfusion, the liver capsule was removed, and the liver was gently swirled in PBS to yield a cell suspension. Hepatocytes were collected by three rounds of centrifugation (50 g for 3 min) and cultured in DMEM/F12 supplemented with 10% fetal bovine serum, 1× ITS (Sigma‒Aldrich), 40 ng/ml dexamethasone (Sigma‒Aldrich) and penicillin/streptomycin.

### Neutrophil isolation

Human neutrophils were isolated from the peripheral blood of healthy volunteers by density gradient separation using Ficoll (Cytiva, 17544202) and centrifugation at 400 × g for 40 min at room temperature. To isolate neutrophils from bone marrow, bone marrow cells from 8- to 12-week-old BALB/c mice were harvested in PBS, and the extraction of neutrophils from bone marrow cells was performed using a Mouse Neutrophil Isolation Kit (Solarbio) according to the manufacturer’s instructions.

### Two-chamber neutrophil migration assays

Neutrophil migration assays were performed using a Transwell migration assay. Briefly, 5×10^5^ freshly isolated neutrophils in RPMI 1640 were added to the upper chamber, and rhLRG1 (20 µg/ml) was added to the lower chamber as a chemoattractant. The migrated cells in the lower chamber were counted after 4 h.

### In vitro NET analysis

To assess NET formation, neutrophils (1×106 cells) were seeded on coverslips coated with poly-L-lysine in 24-well plates for 30 min before rhLRG1 (20 µg/ml), PAD4i and SB431542 were added. After 6 to 8 h at 37 °C, the neutrophils were fixed with 4% paraformaldehyde (PFA) for 10 min at room temperature, washed twice with PBS, blocked in PBS containing 2% BSA for 30 min, and then incubated with anti-H3cit (1:100, ab5103, Abcam) and anti-MPO (10 µg/ml, AF3667, R&D) antibodies in blocking buffer overnight at 4 °C. After three washes in PBS, the cells were incubated with fluorochrome-conjugated secondary antibodies for 1 h and then counterstained with DAPI. Observation and imaging were performed with a confocal microscope (Cell Observer; ZEISS, Germany).

### Transwell migration assays

For DLD1 cells and HCT116 cells (5×10^4^), Transwell migration assays were performed. DLD1 cells and HCT116 cells were plated in the upper wells for 24 h. After the cells adhered, the medium was replaced with 300 µl of serum-free conditioned medium supplemented with neutrophils (1×10^5^), rhLRG1 (20 µg/ml) or DNase (0.25 mg/ml). Complete medium supplemented with 10% FBS was added to the bottom to induce chemotaxis. After 48 h, the cells on the upper surface of the membrane were removed, and the cells that crossed the membrane were fixed with 4% paraformaldehyde and stained with crystal violet. The number of penetrated cells was counted under a light microscope in three fields of view, and the average number of cells was calculated.

### Cytokine array analyses

Serum from the two CRC models was obtained by centrifugation and stored at −80 °C for use in cytokine assays. This was performed using the Mouse Inflammation Array GS1 (Raybiotech, Peachtree Corners, GA, USA) following the instructions of the manufacturer (Wayen Biotechnologies Inc., Shanghai, China).

### RNA-seq data analysis

Raw RNA-seq sequencing data (FASTQ format) were aligned to the mouse reference genome mm10 using HISAT2 (v2.1.0) with default parameters to ensure alignment accuracy. Exon-aligned uniquely mapped reads were quantified using featureCounts (Subread package v2.0.6) to generate the raw count matrix. Gene expression data were normalized to transcripts per million (TPM), followed by hierarchical clustering analysis and heatmap visualization using pheatmap (v1.0.12). For the public dataset GSE109480 (retrieved from the GEO database), its raw expression matrix underwent identical TPM normalization, and differential gene expression bar plots were generated using ggplot2 (v3.5.0).

### Single-cell sequencing data analysis

Single-cell sequencing data were processed using the Seurat (v4.2.0) pipeline. Strict quality control was applied: low-quality cells with total UMI counts <1000 or detected genes <200 were filtered out, along with apoptotic or damaged cells whose mitochondrial gene content was >25%. Red blood cells with hemoglobin gene expression (e.g., Hba-a1 and Hbb-bt) >1% were excluded. To mitigate doublet interference, DoubletFinder (v2.0.3) was employed to predict doublets.

### Differential analysis and pathway enrichment

Differentially expressed genes (DEGs) were identified using the Wilcoxon rank-sum test via Seurat’s FindMarkers function, focusing on neutrophil subsets whose expression was significantly upregulated in the *Lrg1*^(+/+)Hep^-PMN and *Lrg1*^(+/+)Hep^-Ctrl groups (adjusted *p* value < 0.05, Benjamini–Hochberg correction; log-fold change [logFC] >0.5). For these DEGs, Gene Ontology (GO) functional enrichment analysis was performed using clusterProfiler (v4.7.1), with Benjamini‒Hochberg-adjusted *q* values < 0.05 as the significance threshold. The top 20 enriched terms were ranked by enrichment factors. For the PMN-MDSC signature score, individual cells were scored using the AddModuleScore function, which calculates the average expression levels of selected genes at the single-cell level and then subtraction by the aggregated expression of control feature sets.

### Pseudotime analysis

We used Monocle3 (v1.3.1) to analyze the pseudotime distribution in the scRNA-seq data and construct cell trajectories to identify state transitions within the neutrophil populations. During trajectory and pseudotime computation, cells with high differentiation potential predicted by CytoTRACE (v0.3.3) were selected as the root nodes.

### Statistical analysis

Statistical tests were carried out using GraphPad Prism (v9.5.0). Unless otherwise stated, the experimental data are presented as the mean ± standard deviation (SD) of at least three biologically independent replicates. To compare the parametric data, a two-tailed unpaired Student’s *t* test was used to determine statistical significance. A *p* value less than 0.05 was considered to indicate statistical significance.

## Supplementary information

Supplementary figures

Supplementary figure legends

Tables

Unprocessed images

## Data Availability

Raw murine liver scRNA-seq and bulk RNA-seq data for the liver have been deposited in GEO (GSE306164 and GSE305183, respectively). This paper does not report the original code. Any additional information required to reanalyze the data reported in this paper is available from the lead contact upon request.
