# Supplementary material for: Hepatocyte-derived LRG1 primes the liver for metastasis and impairs immunotherapy
Source: Cell Mol Immunol. 2026 Apr 10;23(5):560–74. doi: 10.1038/s41423-026-01408-9 (PMC13129104; doi:10.1038/s41423-026-01408-9)

Fig S1: Pre-metastatic niche formation in liver with tumor progression in mouse models , related to Fig. 1.

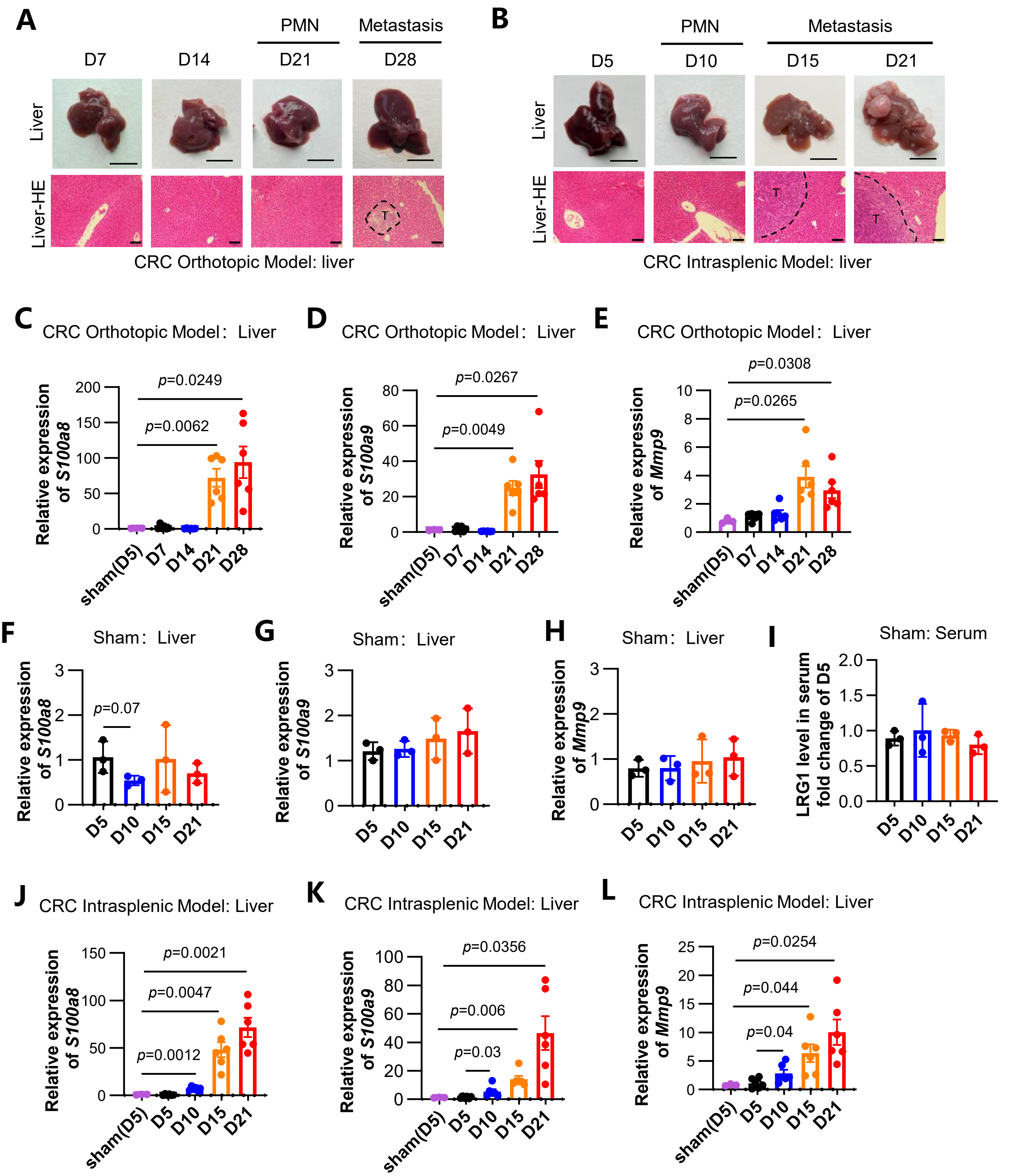

Fig S2: Serological LRG1 is associated with pre-metastatic niche formation in liver, related to Fig. 1.

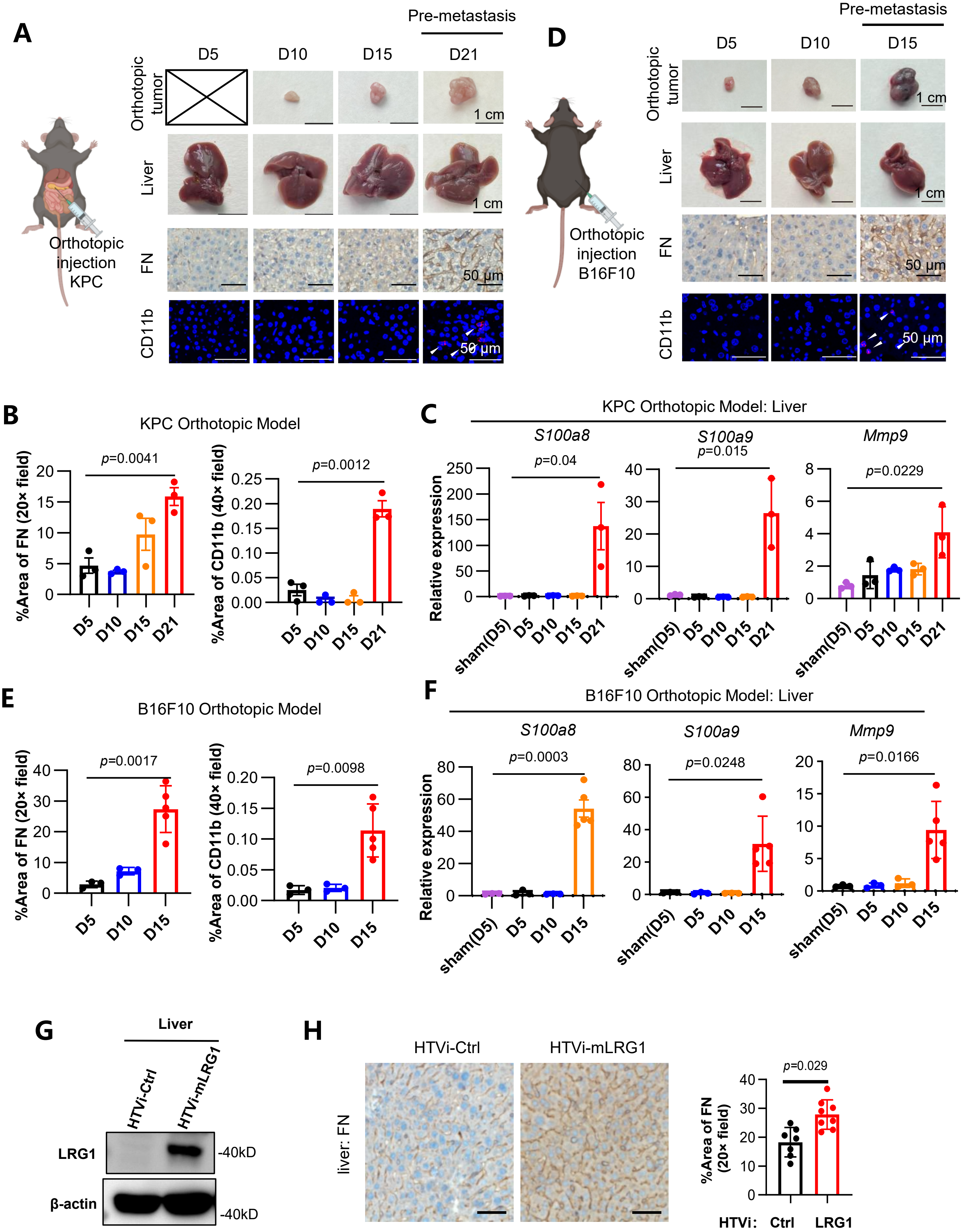

Fig S3: LRG1 is derived from hepatocytes and promotes CRLM, related to Fig. 2.

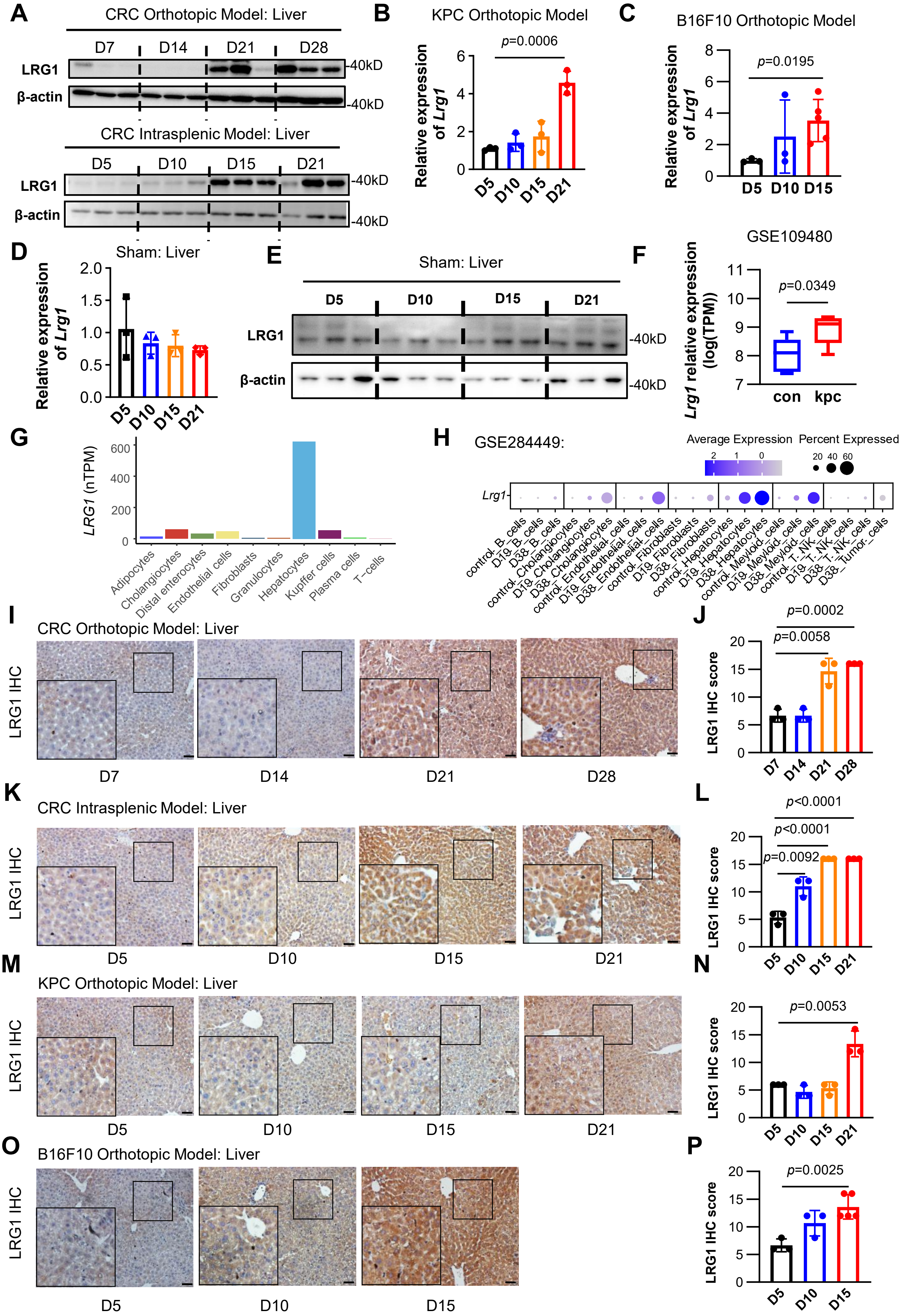

Fig S4: Hepatic-specific LRG1 depletion in mouse model, related to Fig. 2.

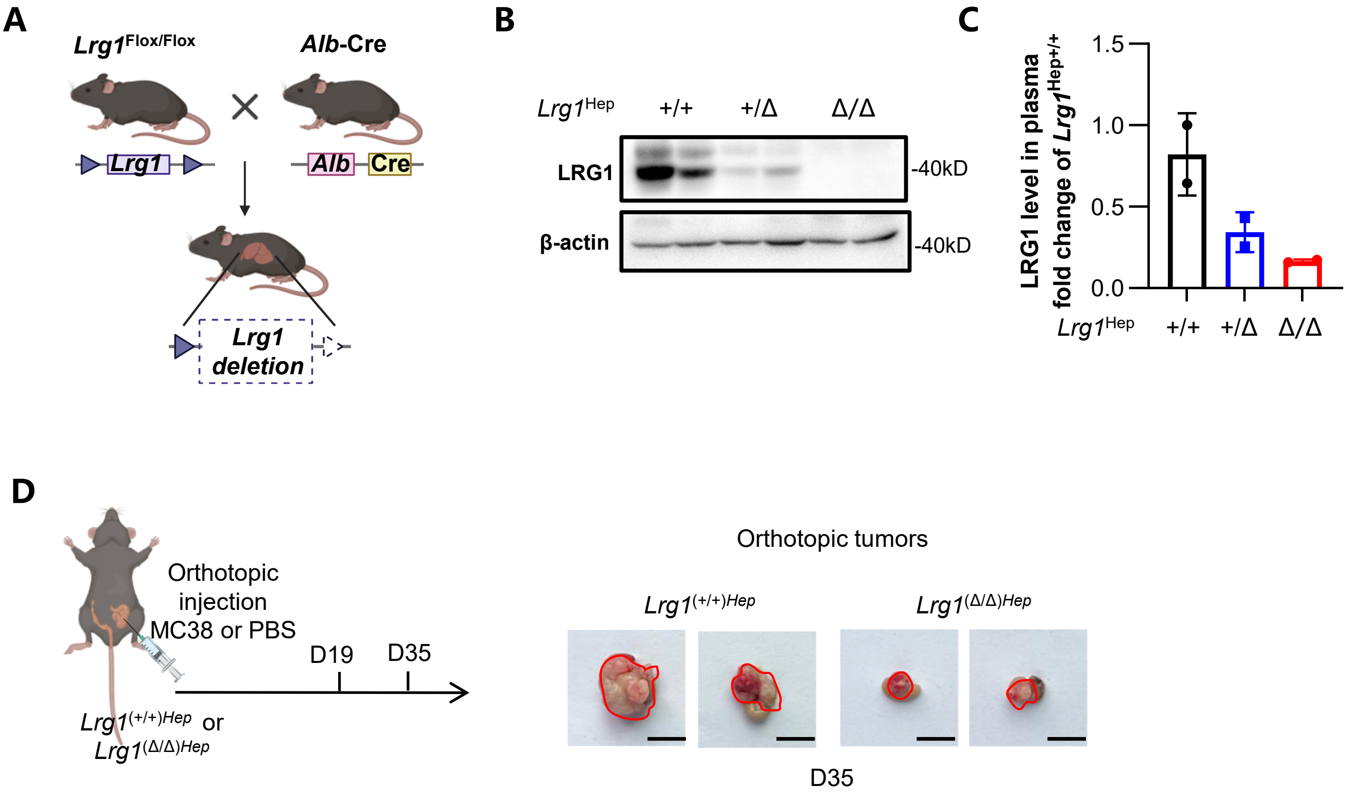

Fig S5: Hepatic LRG1 drives the formation of pre-metastatic niche in the liver, related to Fig. 3.

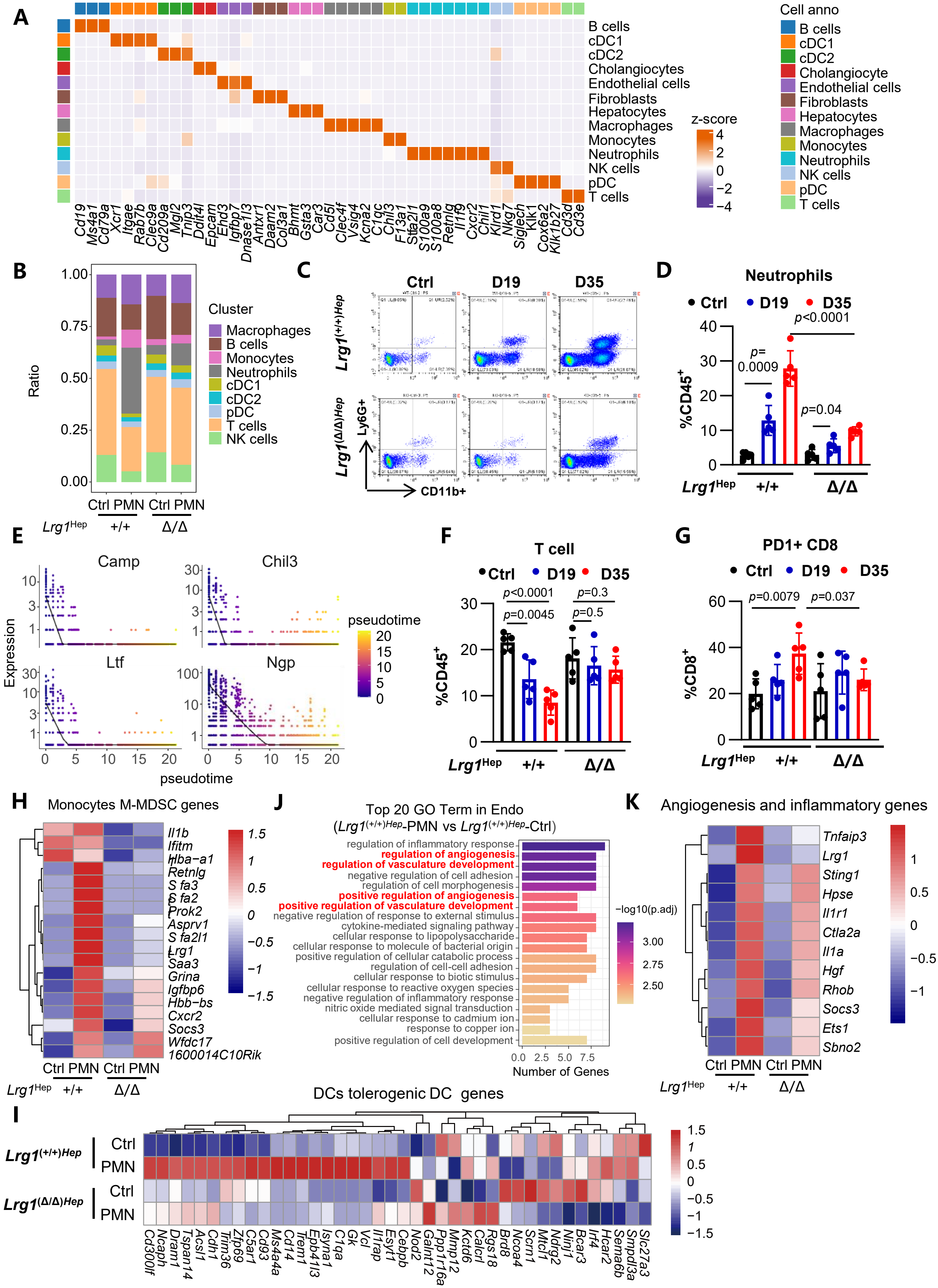

Fig S6: LRG1 promotes NET formation of neutrophils via TGFBR/AKT signaling, related to Fig. 4.

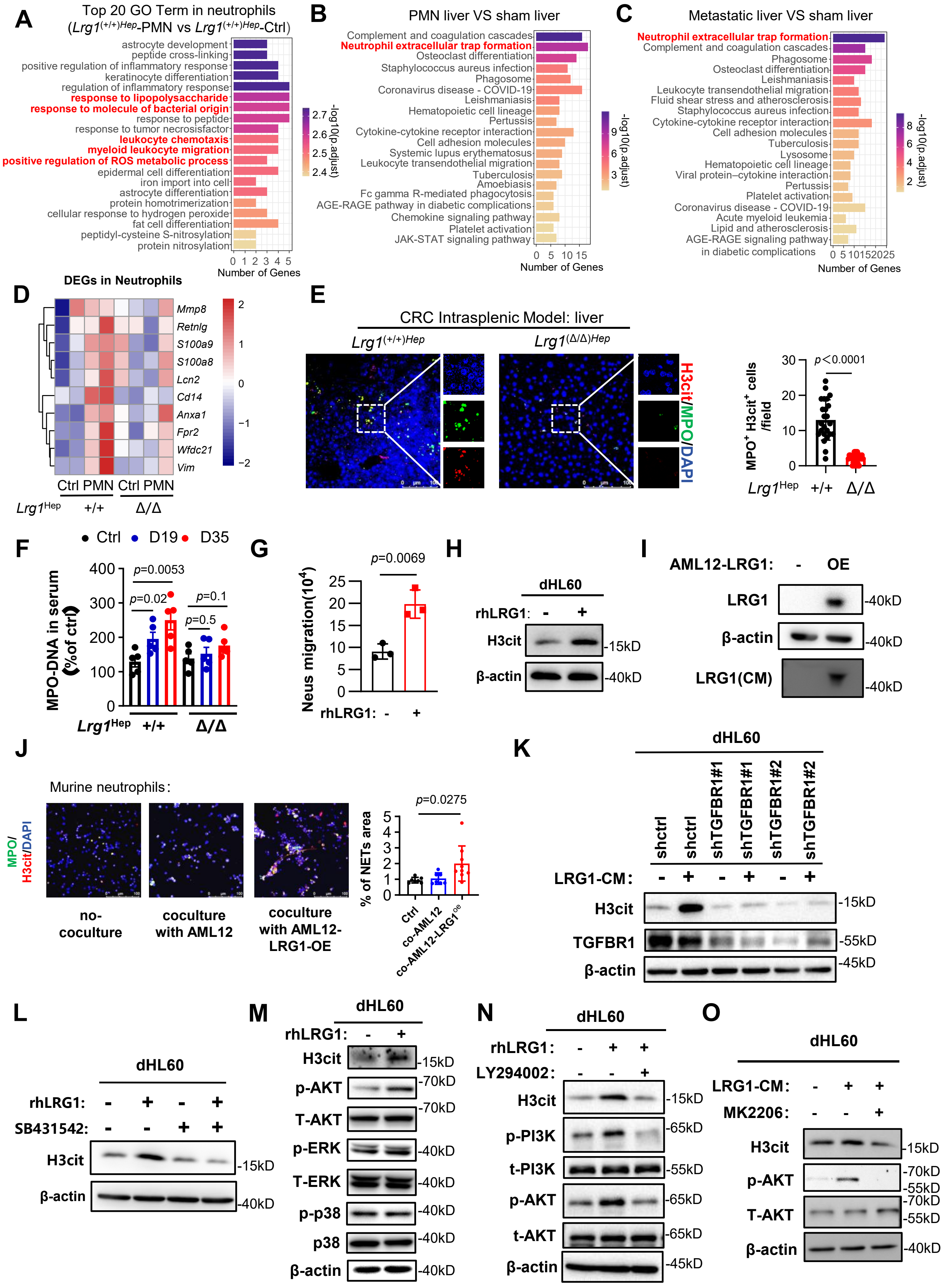

Fig S7: LRG1 promotes tumor cell migration through directing NET formation of neutrophils, related to Fig. 4.

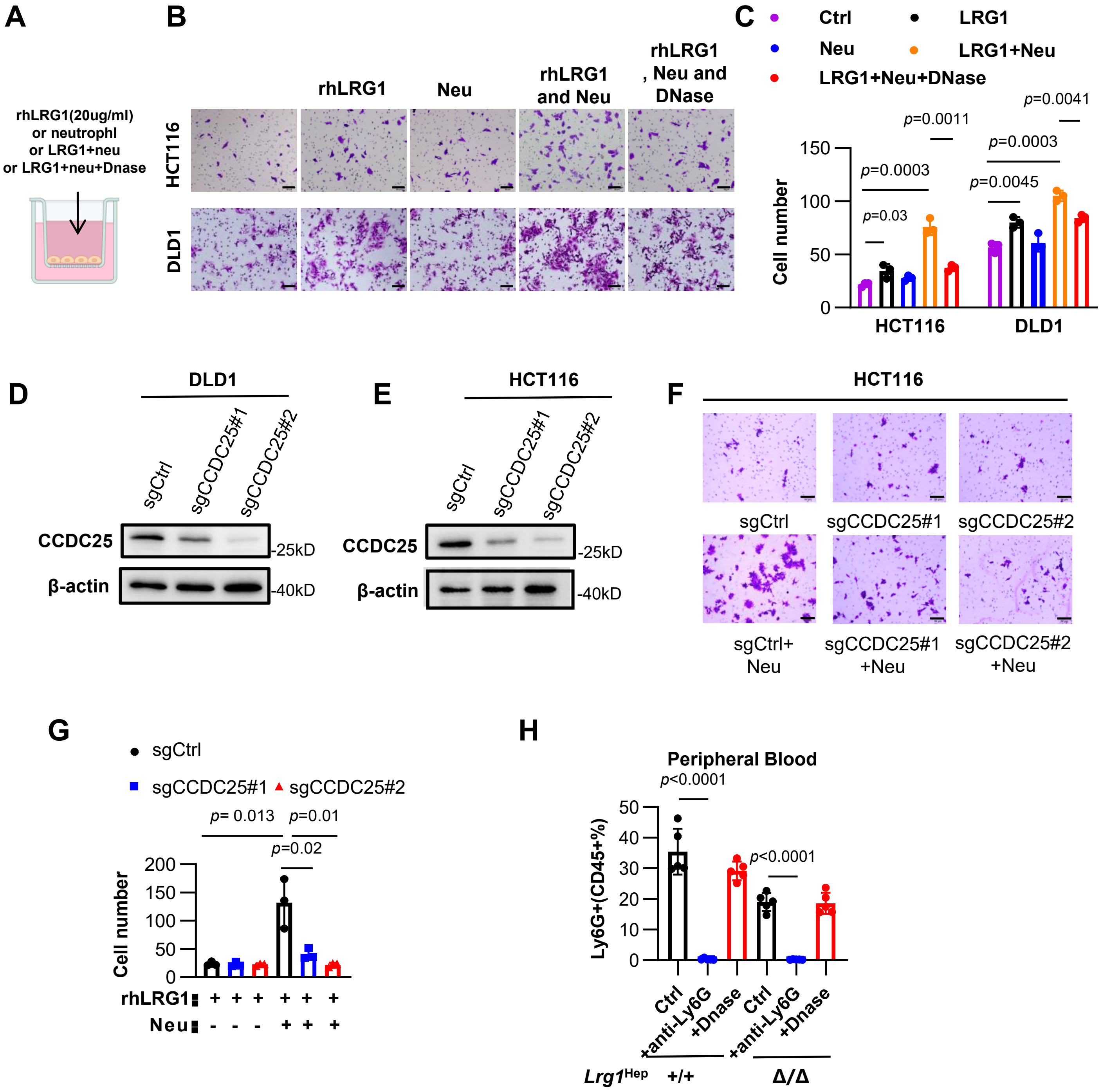

Fig S8: The IL6/STAT3 pathway promotes expression of LRG1 in hepatocytes, related to Fig. 5.

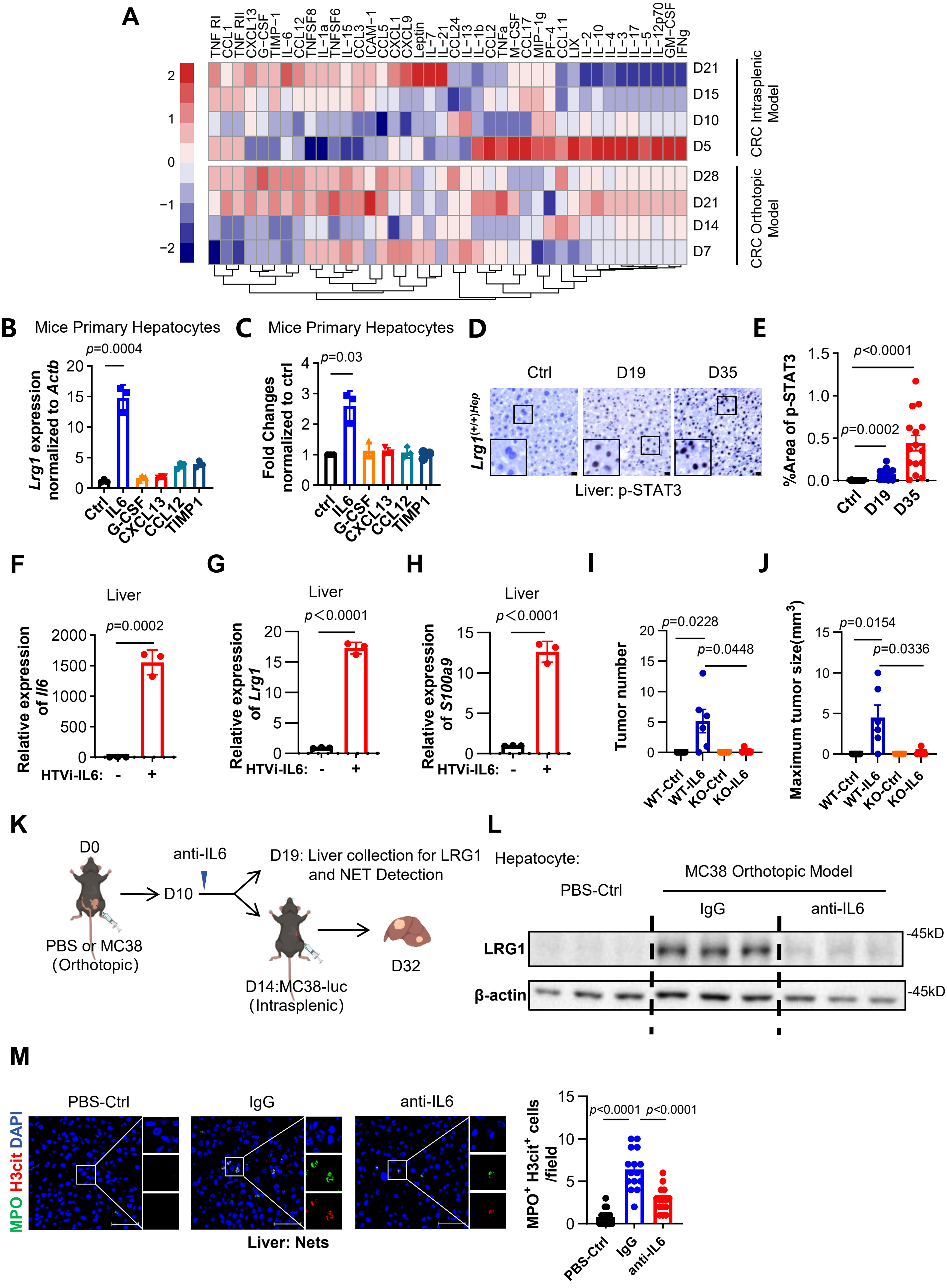

Fig S9: Macrophage-derived IL6 induced hepatic LRG1 expression related to Fig. 5.

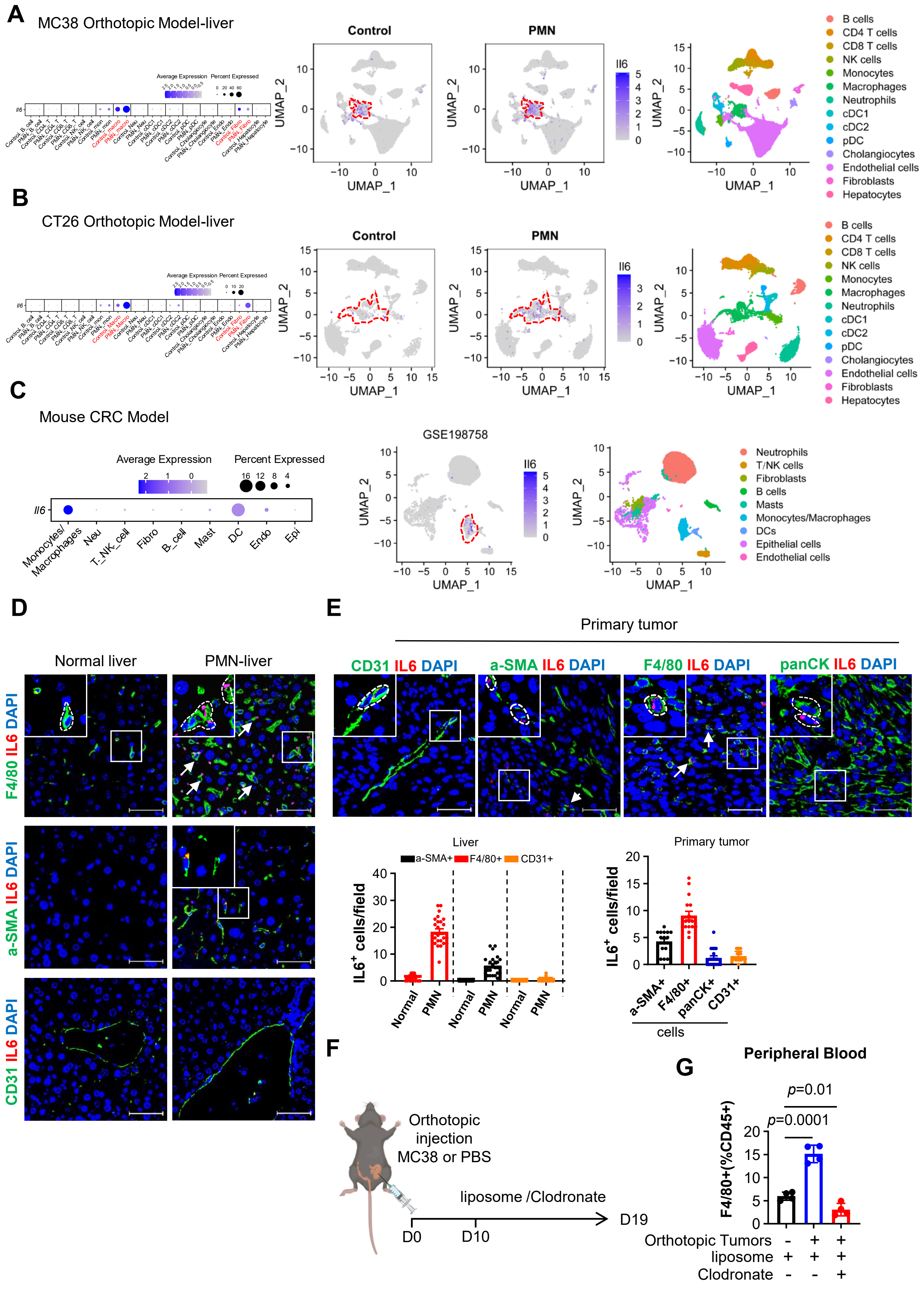

Fig S10: Targeting LRG1 reduces colorectal cancer liver metastasis, related to Fig. 6.

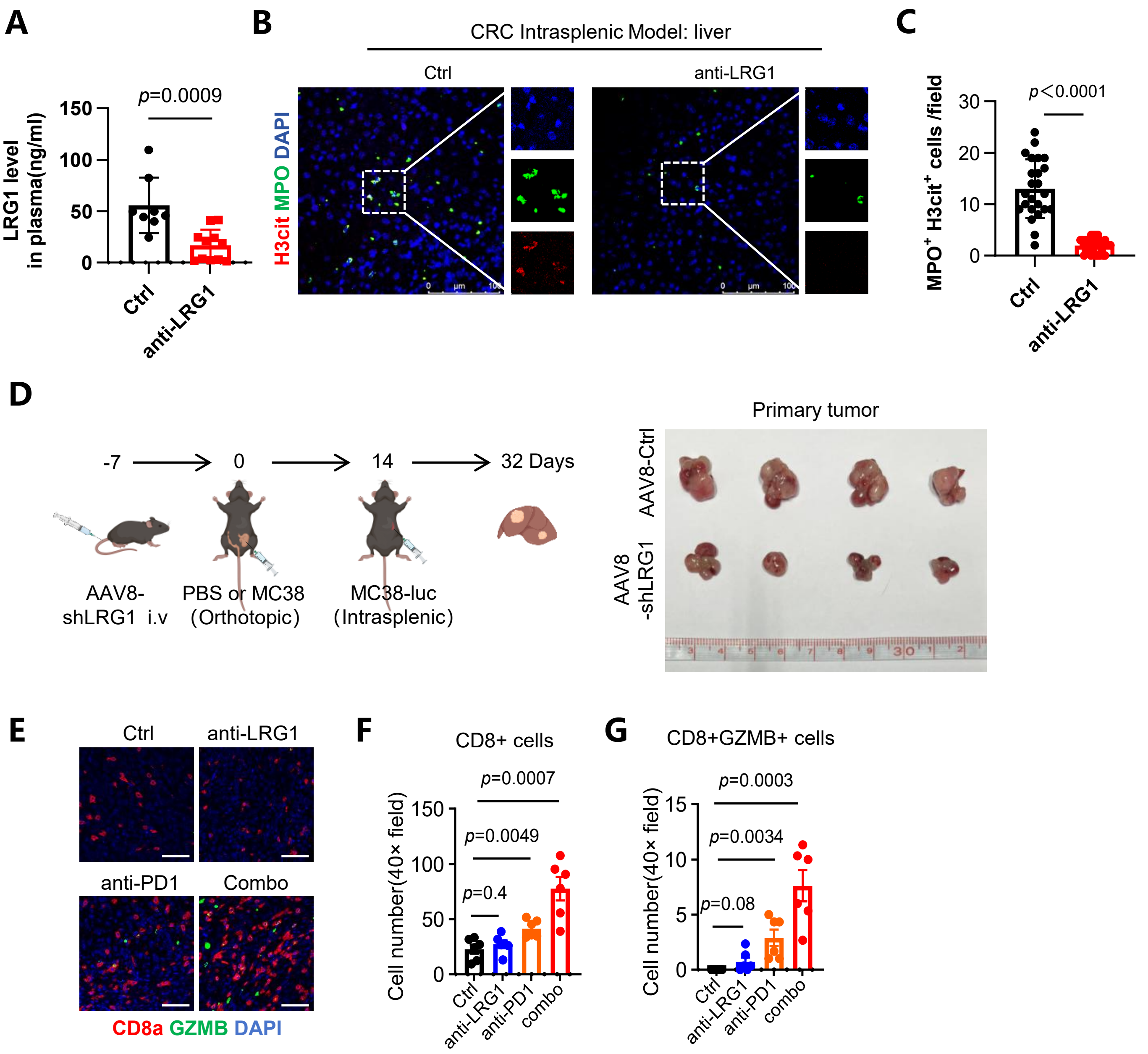

Supplement: Supplementary file 1 — Supplementary figures [file 41423_2026_1408_MOESM1_ESM.pdf]
