# Supplementary material for: Hepatocyte-derived LRG1 primes the liver for metastasis and impairs immunotherapy
Source: Cell Mol Immunol. 2026 Apr 10;23(5):560–74. doi: 10.1038/s41423-026-01408-9 (PMC13129104; doi:10.1038/s41423-026-01408-9)
Supplement: Supplementary file 2 — Supplementary figure legends [file 41423_2026_1408_MOESM2_ESM.pdf]

Fig S1: Pre-metastatic niche formation in liver with tumor progression in mouse models, related to Fig. 1.

(A) Representative Images of liver and H&E staining in the liver of CRC orthotopic model. T, tumor. Liver scale bars, 1 cm. H&E scale bars, 50 μm.
(B)Representative Images of liver and H&E staining in the liver of CRC intrasplenic model. T, tumor. Liver scale bars, 1 cm. H&E scale bars, 50 μm.
(C-E) The relative expression of *S100a8*, *S100a9* and *Mmp9* (normalized to *Actb* expression) in liver of CRC orthotopic model was analyzed by qRT-PCR. n=6 from two independent experiments. Data are means ± SEM.
(F-H) The relative expression of *S100a8*, *S100a9* and *Mmp9* (normalized to *Actb* expression) in liver of sham surgery group was analyzed by qRT-PCR. n=3. Data are means ± SD.
(I) ELISA analysis of serum samples for LRG1 from sham surgery mice at day 5, day 10, day 15 and day 21. n=3. Data are means ± SD.
(J-L) The relative expression of *S100a8*, *S100a9* and *Mmp9* (normalized to *Actb* expression) in liver of CRC intrasplenic model was analyzed by qRT-PCR. n=6 from two independent experiments. Data are means ± SEM.
p values were obtained by two-tailed unpaired Student's *t* test.

Fig S2: Serological LRG1 is associated with pre-metastatic niche formation in liver, related to Fig. 1.

(A) Schematic of KPC orthotopic model (left). Representative images of livers and orthotopic tumors (upper right). Representative images of IHC staining of FN and immunofluorescence staining of CD11b (Arrows indicate CD11b+ cells) in the liver of KPC orthotopic model (bottom right). Scale bars are as indicated.
(B) Quantification of FN and CD11b in A, n=3. Data are means ± SEM.
(C) The relative expression of *S100a8*, *S100a9* and *Mmp9* (normalized to *Actb* expression) in liver of KPC orthotopic model was analyzed by qRT-PCR. Data are means ±SEM.
(D) Schematic of B16F10 orthotopic model (left). Representative images of livers and orthotopic tumors (upper right). Representative images of IHC staining of FN and immunofluorescence staining of CD11b (Arrows indicate CD11b+ cells) in the liver of B16F10 orthotopic model (bottom right). Scale bars are as indicated.
(E) Relative quantification of FN and CD11b in D, n=3 on day 5, 10 and n=5 on day 15. Data are means ± SD.
(F) The relative expression of *S100a8*, *S100a9* and *Mmp9* (normalized to *Actb* expression) in liver of B16F10 orthotopic model was analyzed by qRT-PCR. n=3 on day 5 and 10 and n=5 on day 15. Data are means ± SEM.
(G) Western blot detected expression of LRG1 and β-actin in liver of HTVi-Ctrl and

HTVi-LRG1.
(H) Representative images and quantification of FN of IHC staining of FN in the liver of HTVi model. n=7 in HTVi-ctrl group and n=8 in HTVi-LRG1 group. Data are means ± SD.
p values were obtained by two-tailed unpaired Student's *t* test.

Fig S3: LRG1 is derived from hepatocytes and promotes CRLM, related to Fig. 2.

(A) Western blot analysis of LRG1 expression in liver of CRC orthotopic model at different days (day 7, day 14, day 21 and day 28) and CRC intrasplenic model on different days (day 5, day 10, day 15 and day 21).
(B) The relative expression of *Lrg1* (normalized to *Actb* expression) in liver of KPC orthotopic model at different days (day 5, day 10, day 15 and day 21). Dots represent individual samples. n=3. Data are means ± SD.
(C)The relative expression of *Lrg1* (normalized to *Actb* expression) in liver of B16F10 orthotopic model at different days (day 5, day 10, and day 15). Dots represent individual samples. n=3 on day 5, 10 and n=5 on day 15. Data are means ± SD.
(D-E) Quantitative real-time PCR and western blot analysis of LRG1 expression in sham surgery group at different days (day 5, day 10, day 15 and day 21) n=3. Data are means ± SD.
(F) mRNA-seq analysis of *Lrg1* expression in liver of KPC mice (GSE109480).
(G) Relative LRG1 expression in different cells in liver. Data source: The Human Protein Atlas database.
(H) scRNA-seq analysis expression of *Lrg1* in different cells from liver of CRC orthotopic model at different days (day 19 and day 38) (GSE284449).
(I-J) Representative images (I) and relative quantification of IHC staining of LRG1(J) in liver of CRC orthotopic model. n=3. Scale bars, 50 μm. Data are means ± SD.
(K-L) Representative images(K) and relative quantification of IHC staining of LRG1(L) in liver of CRC intrasplenic model. n=3. Scale bars, 50 μm. Data are means ± SD.
(M-N) Representative images(M) and relative quantification of IHC staining of LRG1(N) in liver of KPC orthotopic model. n=3. Scale bars, 50 μm. Data are means ± SD.
(O-P) Representative images(O) and relative quantification of IHC staining of LRG1(P) in liver of B16F10 orthotopic model. n=3 on day 5, 10 and n=5 on day 15. Scale bars, 50 μm. Data are means ± SD.
p values were obtained by two-tailed unpaired Student's *t* test.

Fig S4: Hepatic-specific LRG1 depletion in mouse model, related to Fig. 2.

(A) Schematic diagram of hepatocyte-specific *Lrg1* knockout mouse model.
(B) Western blot detected expression of LRG1 and β-actin in liver of *Lrg1*$^{(+/+)Hep}$, *Lrg1*$^{(+/\Delta)Hep}$, and *Lrg1*$^{(\Delta/\Delta)Hep}$ mice. n=2.
(C) ELISA analysis of serum samples for LRG1 from *Lrg1*$^{(+/+)Hep}$, *Lrg1*$^{(+/\Delta)Hep}$, and *Lrg1*$^{(\Delta/\Delta)Hep}$ mice. n=2.
(D) Representative images of orthotopic tumors in Figure 2F.

Fig S5: Hepatic LRG1 drives the formation of pre-metastatic niche in the liver, related to Fig. 3.

(A) Cell type annotation markers.
(B) Proportion of immune cell subpopulations in each group.
(C-D) Quantification of Neutrophils by flow cytometry in liver of mice from different groups as indicated in Fig 2F. n=5 per group. Data are means ± SD.
(E) Example expression dynamics of selected genes along neutrophil pseudotime.
(F-G) Quantification of T cells, and PD1+ T cells by flow cytometry in liver of mice from different groups as indicated in Fig 2F. n=5 per group. Data are means ± SD.
(H) Heatmap of M-MDSC genes in monocytes of each groups.
(I) Heatmap of tolerogenic DC genes in DCs of each groups.
(J) Gene Ontology (GO) analysis of enriched pathways of endothelial cells in liver from *Lrg1*$^{(+/+)Hep}$-PMN and *Lrg1*$^{(+/+)Hep}$-Ctrl group.
(K) Heatmap of angiogenesis and inflammatory genes in endothelial cells of each group.
p values were obtained by two-tailed unpaired Student's *t* test.

Fig S6: LRG1 promotes NETs formation of neutrophils via TGFBR/AKT signaling, related to Fig. 4.

(A) Gene Ontology (GO) analysis of enriched pathways of neutrophils in liver from *Lrg1*$^{(+/+)Hep}$-PMN and *Lrg1*$^{(+/+)Hep}$-Ctrl group.
(B-C) KEGG pathway analysis from bulk RNA-seq of PMN versus sham liver tissue (B) and metastasis liver versus sham liver (C), highlighting significant alteration in NETs.
(D) Heatmap of selected genes of enriched pathways in (Figure S6A).

(E) Representative images and quantification of Immunofluorescence staining of MPO (green) and H3cit (red) in liver from CRC Intrasplenic Model. Dots represent field of view (FOV) from all samples (n=6 in *Lrg1*$^{(+/+)Hep}$ group, n=9 in *Lrg1*$^{(\Delta/\Delta)Hep}$ group). Data are means ± SEM.
(F) Serological levels of MPO-DNA in mice from different groups as indicated in Fig 2F. Data are means ± SEM.
(G) Migration of human neutrophils recruited by rhLRG1. n=3 independent experiments, Data are means ± SD.
(H) Western blot analysis of the expression H3cit and β-actin in dHL60 cells treated with rhLRG1.
(I) Western blot analysis showing the expression of LRG1 and β-actin in AML12-ctrl, AML12-LRG1oe cells and conditioned medium.
(J) Immunofluorescence analyses of NETs formation in murine neutrophils cocultured with or without AML12-ctrl or AML12-LRG1oe cells. Dots represent field of view (FOV) from 3 independent experiments. Data are means ± SD.
(K) Western blot detected expression of H3cit, TGFBR1 and β-actin in dHL60-shTGFBR1 cells treated with or without LRG1- conditioned medium (CM).
(L) Western blot analysis of the expression H3cit and β-actin in dHL60 cells after the indicated treatments.
(M) Western blot analysis showing the expression of H3cit, p-AKT, t-AKT, p-ERK, t-ERK, p-p38, t-p38 and β-actin in dHL60 treated with or without rhLRG1.
(N) Western blot analysis showing the expression of H3cit, p-AKT, t-AKT, p-PI3K, t-PI3K and β-actin in dHL60 treated with or without rhLRG1 and LY294002.
(O) Western blot analysis showing the expression of H3cit, p-AKT, t-AKT and β-actin in dHL60 treated with or without rhLRG1 and MK2206.
p values were obtained by two-tailed unpaired Student's *t* test.

Fig S7: LRG1 promotes tumor cell migration through directing NETs formation of neutrophils, related to Fig. 4.

(A-C) Schematic of transwell migration assays (A). Transwell migration assays of DLD-1 and HCT116 cells treated under the indicated conditions (B and C). Neu, neutrophils. n=3 independent experiments. Scale bars, 50 μm. Data are means ± SD.
(D-E) Western blot detected expression of CCDC25 and β-actin in DLD-1-sgCCDC25 and HCT116-sgCCDC25 cells.
(F-G) Transwell migration assays of HCT116-sgCCDC25 cells treated with depicted condition (F and G). Neu, neutrophils. n=3 independent experiments. Scale bars, 50 μm. Data are means ± SD.
(H) Neutrophils were quantified by flow cytometry in peripheral blood of mice indicated in Fig 4N. Data are means ± SD.
p values were obtained by two-tailed unpaired Student's *t* test.

Fig S8: The IL6/STAT3 pathway promotes expression of LRG1 in hepatocytes, related to Fig. 5.

(A) Cytokine array analysis of serum from CRC orthotopic mice model (day 7, day 14, day 21 and day 28) and from CRC intrasplenic mice model (day 5, day 10, day 15 and day 21).
(B-C) Quantitative real-time PCR analyses of the expression *Lrg1* (B) and ELISA analyses of media (C) of mice primary hepatocytes treated with vehicle or recombinant IL6/G-CSF/CXCL13/CCL12/TIMP1. n=3 independent experiments. Data are means ± SD.
(D-E) Representative images of IHC staining (D) and quantification of p-stat3 in liver (E). Scale bars 10 μm. Dots represent field of view (FOV) from 5 sample per group. Data are means ± SEM.
(F-H) The relative expression of *Il6*, *Lrg1* and *S100a9* (normalized to *Actb* expression) in liver of HTVi IL6-overexpression (OE) mice model was analyzed by qRT-PCR. n=3. Data are means ± SD.
(I-J) Quantification of the number (I) and maximum tumor size (J) of liver metastases in each group in Fig 5L. Data are means ± SEM.
(K-M) Schematic of the experimental design (K). Western blot detected expression of LRG1 and β-actin in hepatocytes from mice in indicated group (L). Representative images and quantification of Immunofluorescence staining of MPO (green) and H3cit (red) in liver. Dots represent field of view (FOV) from 3 samples per group (M). Scale bars, 50 μm. Data are means ± SEM.
p values were obtained by two-tailed unpaired Student's *t* test.

Fig S9: Macrophage-derived IL6 induced hepatic LRG1 expression related to Fig. 5.

(A-B) scRNA-seq analysis expression of *Il6* in different cells from liver of MC38 CRC orthotopic model (A) and CT26 CRC orthotopic model (B). The red dotted circle encloses the macrophages.
(C) scRNA-seq analysis expression of *Il6* in different cells from primary tumor of mice CRC model. The red dotted circle encloses the Monocytes and macrophages.
(D-E) Representative images of immunofluorescence co-staining of F4/80, a-SMA or CD31 and IL6 in normal liver and PMN liver (D). Representative images of immunofluorescence co-staining of F4/80, a-SMA, panCK or CD31 and IL6 in primary tumor (E). Shown are the quantification of co-stained cell numbers in all field of view

(FOV).

(F-G) Schematic of CRC orthotopic model treated with liposome Clodronate (F). Macrophages were quantified by flow cytometry in peripheral blood of mice (G). n=4. Data are means ± SD.

p values were obtained by two-tailed unpaired Student's *t* test.

Fig S10: Targeting LRG1 reduces colorectal cancer liver metastasis, related to Fig. 6.

(A) ELISA analysis of serological levels of LRG1 in mice from different groups as indicated in Fig 6A. Data are means ± SD.

(B-C) Representative images (B) and quantification (C) of Immunofluorescence staining of MPO (green) and H3cit (red) in liver from different groups as indicated in Fig 6A. DAPI is shown in blue. Scale bars, 100 μm. Dots represent field of view (FOV) from all samples. Data are means ± SD.

(D) Representative images of orthotopic tumors in Figure 6E.

(E-G) Representative images and quantification of immunofluorescence staining of CD8a (red) and GZMB (green) in tumor from different groups as indicated in Fig 6K. n=6, Scale bars, 50 μm. Data are means ± SEM.

p values were obtained by two-tailed unpaired Student's *t* test.
