# Supplementary material for: Hepatocyte-derived LRG1 primes the liver for metastasis and impairs immunotherapy
Source: Cell Mol Immunol. 2026 Apr 10;23(5):560–74. doi: 10.1038/s41423-026-01408-9 (PMC13129104; doi:10.1038/s41423-026-01408-9)
Supplement: Supplementary file 3 — Tables [file 41423_2026_1408_MOESM3_ESM.pdf]

Table S1 Primer sequences. Primer sequences used in qRT-PCR and PCR assays.

Gene	Forward primer (5’-3’)	Reverse primer (5’-3’)	Application
S100a8	CTTCAAGACATCGTTTGAAAGG	AGACATCAATGAGGTTGCTC	qRT-PCR
S100a9	GCACAGTTGGCAACCTTTATG	TGATTGTCCTGGTTTGTGTCC	qRT-PCR
Mmp9	TGATGTTATGATGGTCCCACTTG	GCAGAGGCATACTTGTACCG	qRT-PCR
Actb	CATTGCTGACAGGATGCAGAAGG	TGCTGGAAGGTGGACAGTGAGG	qRT-PCR
Lrg1	CCATGTCAGTGTGCAGATTC	AAGAGTGAGAGGTGGAAGAG	qRT-PCR
Il6	TACCACTTCACAAGTCGGAGGC	CTGCAAGTGCATCATCGTTGTTC	qRT-PCR
**Genotyping primers**			
Alb^{cre}	TGGATGCCACCTCTGATGAAGTC	TCCTGGCATCTGTCAGAGTTCTCC	PCR
Alb^{cre}	GGCCTCCAAGTCTTGACAGTAGAT	CTTGTGGGTCTTCCACCTTTCTTC	PCR
Lrg1$^{fl/fl}$	CAGGCTTGAGGCTTTGGTCATA	CCCTATCTCCCTAGTTGTCTGTTCAG	PCR

Table S2 Sequences of sgRNA or shRNA

gRNA or shRNA	Sequences (5'-3')
CCDC25-gRNA-1	caccgGATGCGAGTGCCTCTTCCCG
CCDC25-gRNA-2	caccgGCGCTCAACTCACGAAGCTC
TGFBR2-shRNA-1	AATGACGAGAACATAACACT
TGFBR2-shRNA-2	AGTATGCCTCTTGGAAGACA
TGFBR1-shRNA-1	CTCATGTTGATGGTCTATATC
TGFBR1-shRNA-2	GAAGTTGCTGTTAAGATATTC

Table S3 Antibodies. The catalogue numbers and dilutions of antibodies

Antibody	Manufacturer	Catalog	Application
Anti-CD11b	Abcam	ab128797	IF:(1:200)
Anti-Fibronectin	Servicebio	GB114491	IHC:(1:200)
Anti-LRG1(mouse)	Abcam	ab231188	WB:(1:1000)<br>IHC:(1:500)
Anti-β-actin	Sigma	A5441	WB:(1:5000)
Anti-CD45	Biolegend	103116	FACS:(1:50)
Anti-Ly6G	BD	560603	FACS:(1:50)
Anti-CD11b	BD	562950	FACS:(1:50)
Anti-CD3e	BD	561100	FACS:(1:50)
Anti-CD8a	BD	563234	FACS:(1:50)
Anti-PD1	Biolegend	135241	FACS:(1:50)
Anti-FVD777	STARTER	SOD0025	FACS:(1:1000)
Anti-CD45	BD	553079	FACS:(1:50)
Anti-CD8a	BD	570255	FACS:(1:50)
Anti-GZMB	BD	561142	FACS:(1:50)
Anti-CD45	BD	561483	FACS:(1:50)
Anti-CD31	BD	558738	FACS:(1:50)
Anti-CD66b	BD	561650	FACS:(1:50)
Anti-iNOS	Servicebio	GB11119	IF:(1:2000)
Anti-MPO	R&D systems	AF3667	IF:(10ug/ml)
Anti-H3cit	Abcam	ab5103	IF:(1:100)<br>WB:(1:1000)
Anti-LRG1(human)	Abcam	ab178698	IF:(1:200)
Anti-CCDC25	Proteintech	21209-1-AP	WB:(1:2000)
Anti-p-AKT	CST	4060	WB:(1:1000)
Anti-AKT	CST	9272	WB:(1:1000)
Anti-p-ERK	CST	4370	WB:(1:1000)
Anti-ERK	CST	4695	WB:(1:1000)
Anti-p-p38 MAPK	CST	9212	WB:(1:1000)
Anti-p38 MAPK	CST	4511	WB:(1:1000)
Anti-p-STAT3	CST	9145	WB:(1:1000)<br>IHC:(1:50)
Anti-STAT3	CST	9139	WB:(1:1000)
Anti-p-SMAD2/3	CST	8828	WB:(1:1000)
Anti-SMAD2/3	CST	8685	WB:(1:1000)
Anti-p-SMAD1/5	CST	9516	WB:(1:1000)
Anti-SMAD1/5	Abcam	Ab80255	WB:(1:1000)
Anti-p-PI3K	CST	4228	WB:(1:1000)

Anti-PI3K	CST	11889	WB:(1:1000)
Anti-TGFBR2	Abclonal	A1415	WB:(1:1000)
Anti-TGFBR2	DIMA Biotech	DMC467	FACS:(1:100)
Anti-CD8a	CST	9894	TSA:(1:2000)
Anti-GZMB	CST	1721	TSA:(1:2000)
Anti-IL6	Servicebio	GB11117	TSA:(1:15000)
Anti-F4/80	Servicebio	GB11027	TSA:(1:5000)
Anti-a-SMA	Servicebio	GB111364	TSA:(1:5000)
Anti-CD31	Servicebio	GB11063	TSA:(1:1000)
Anti-panCK	Huabio	ER1914-74	TSA:(1:3000)
Anti-TGFBR1	Servicebio	GB11271	WB:(1:1000)

.
