# Supplementary material for: Hepatocyte-derived LRG1 primes the liver for metastasis and impairs immunotherapy
Source: Cell Mol Immunol. 2026 Apr 10;23(5):560–74. doi: 10.1038/s41423-026-01408-9 (PMC13129104; doi:10.1038/s41423-026-01408-9)

Fig 1I:

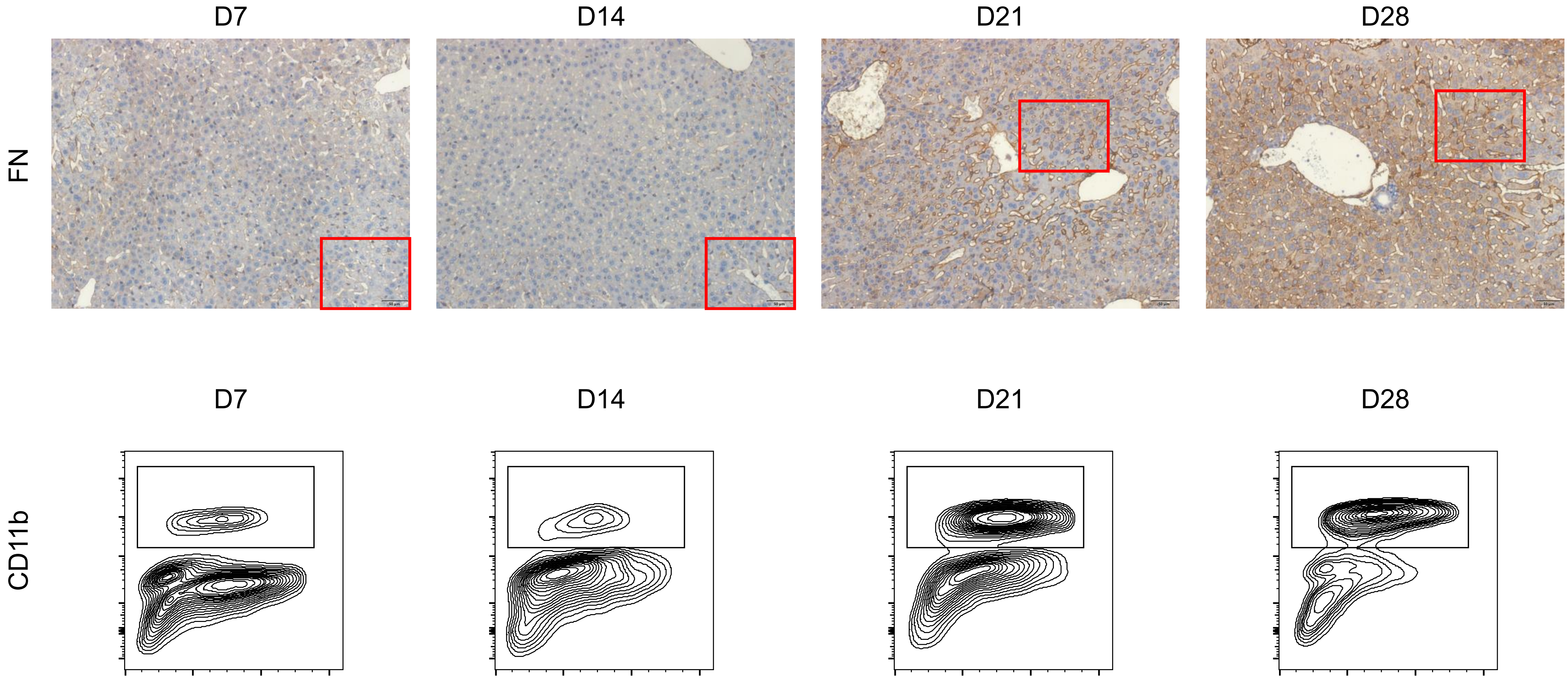

Fig 1N

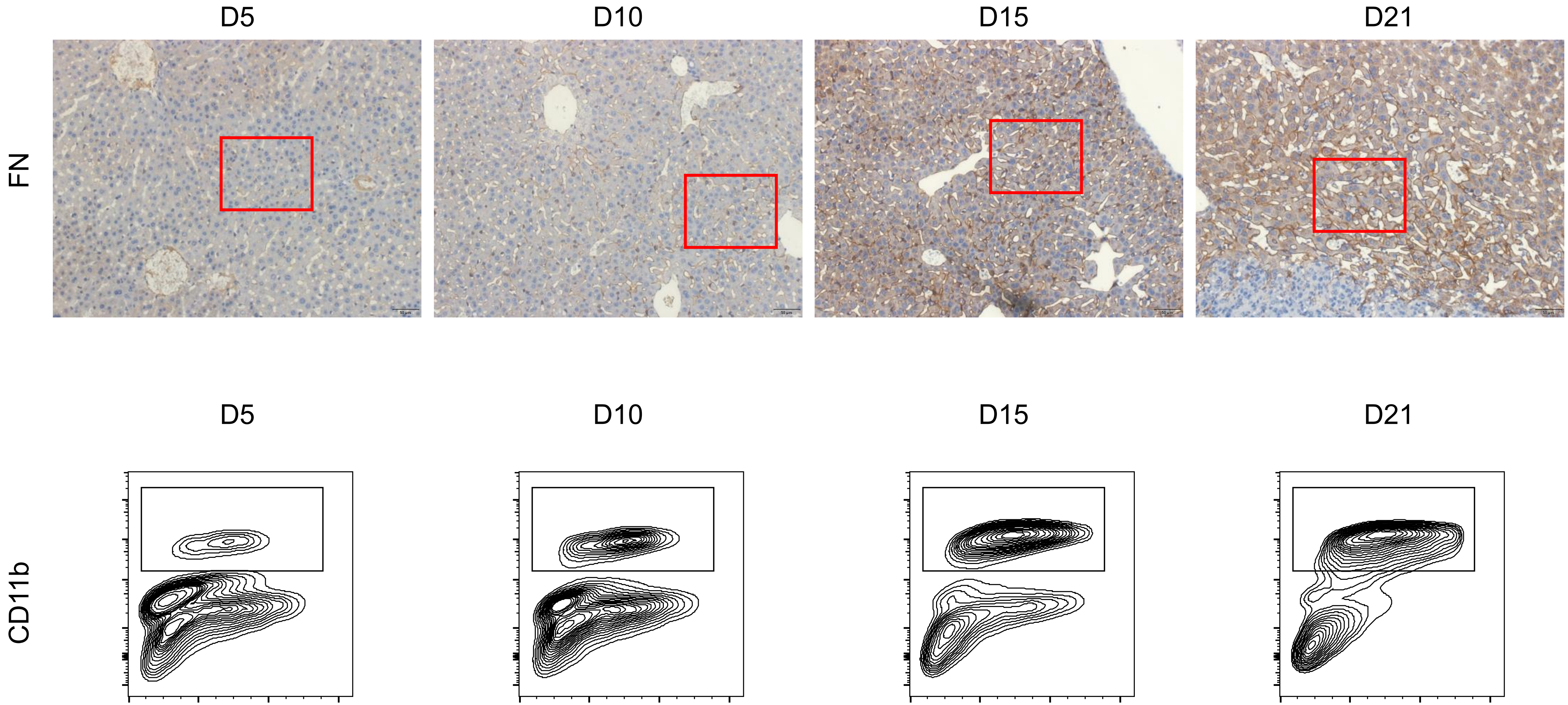

Fig 1V

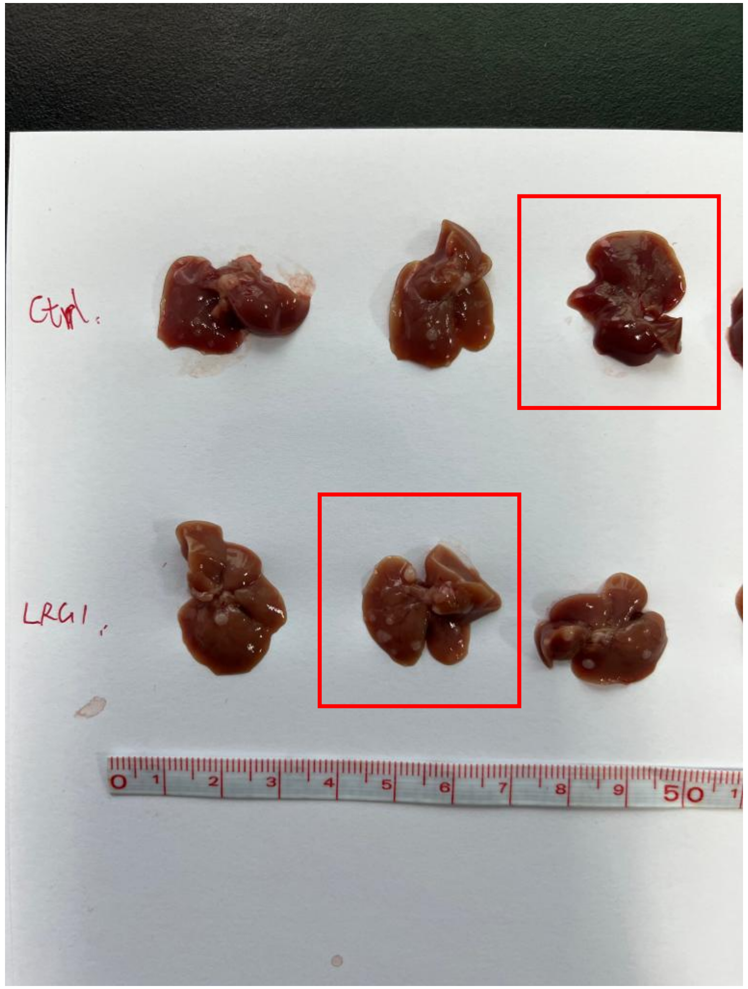

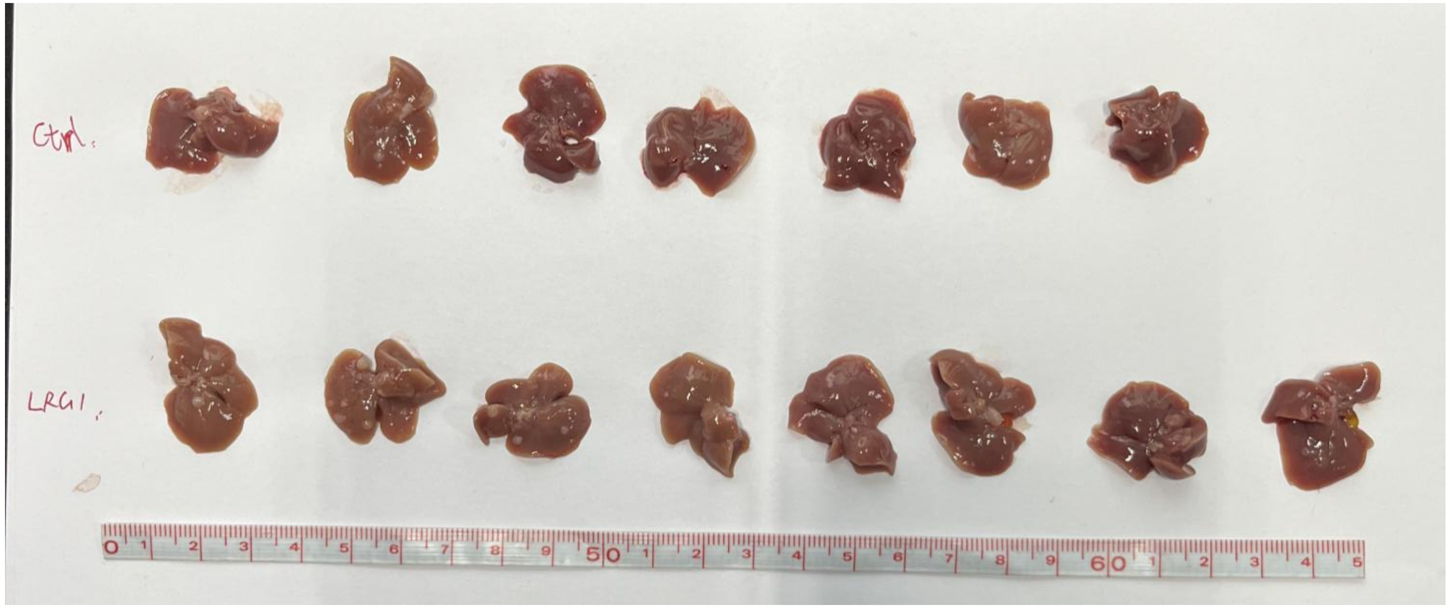

Fig 2E

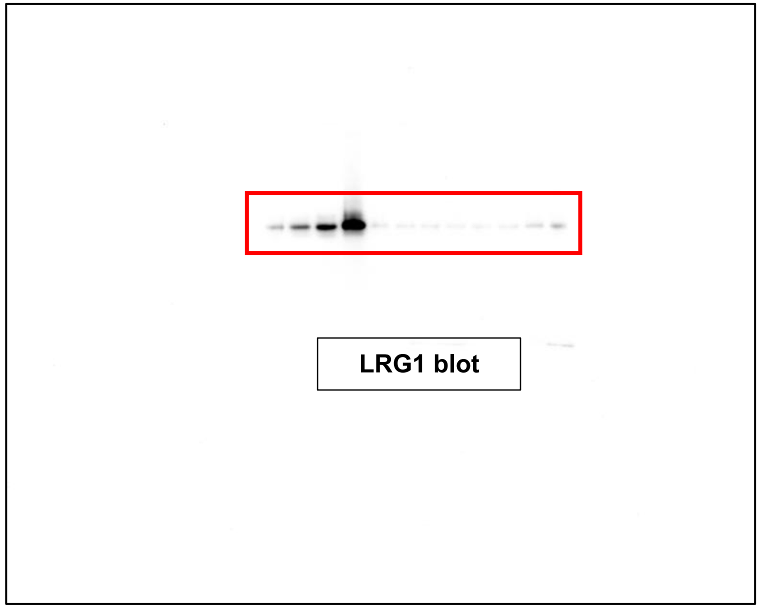

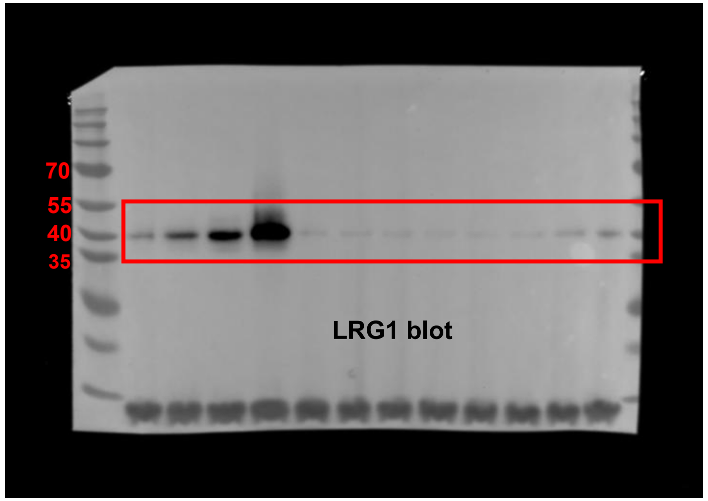

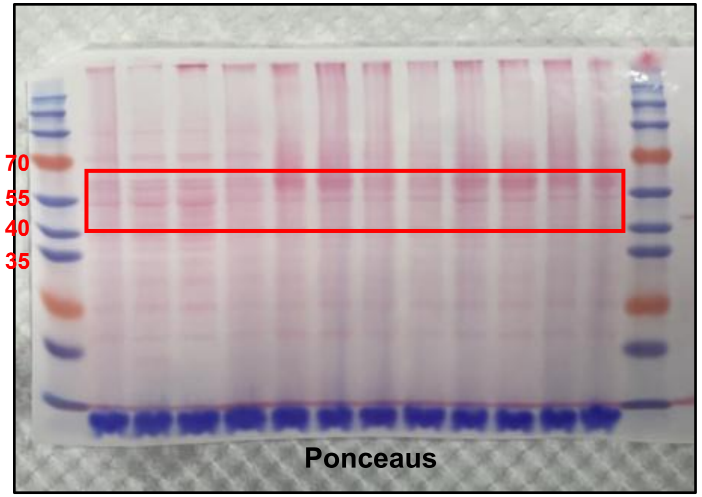

Fig 2J

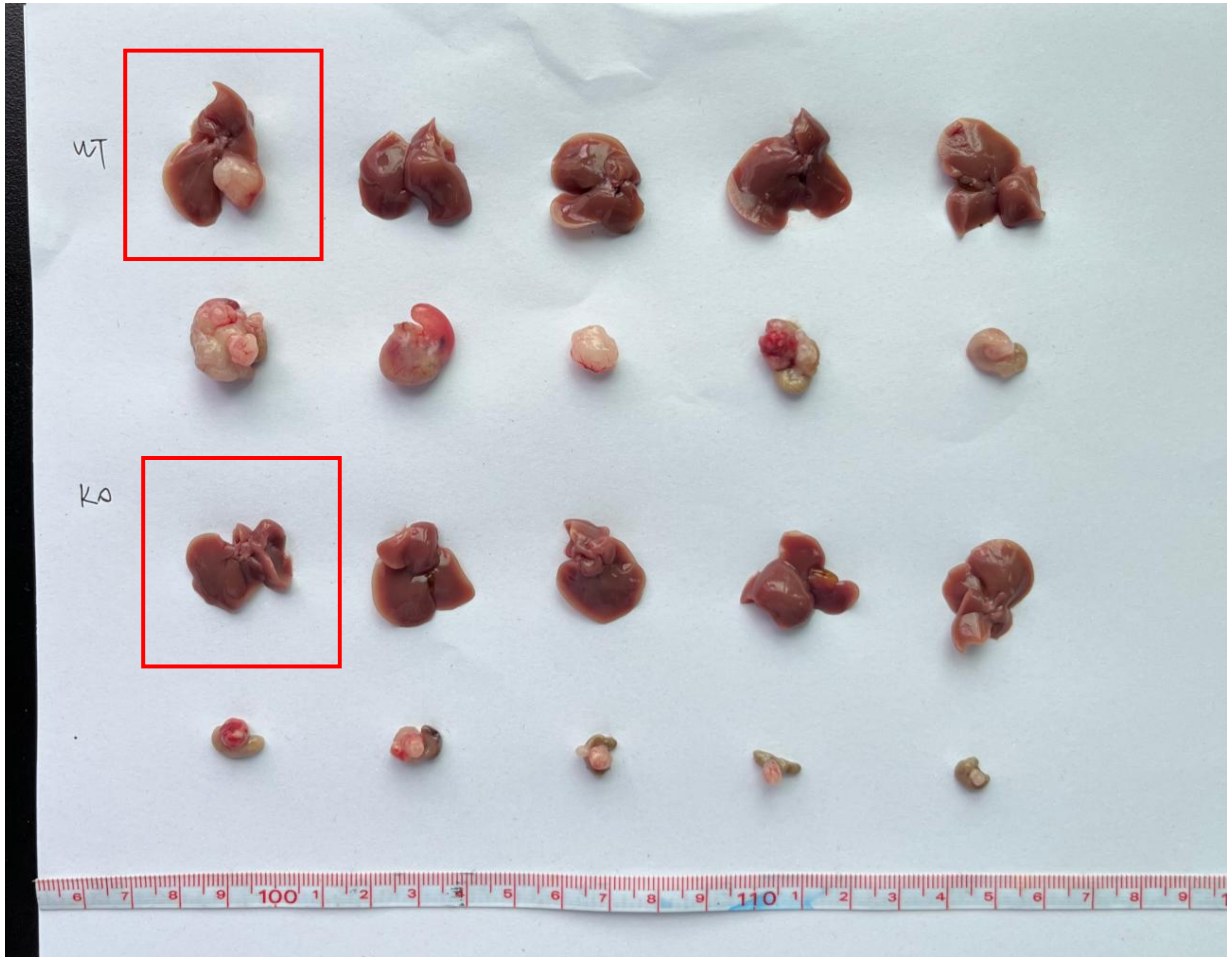

Fig 2G

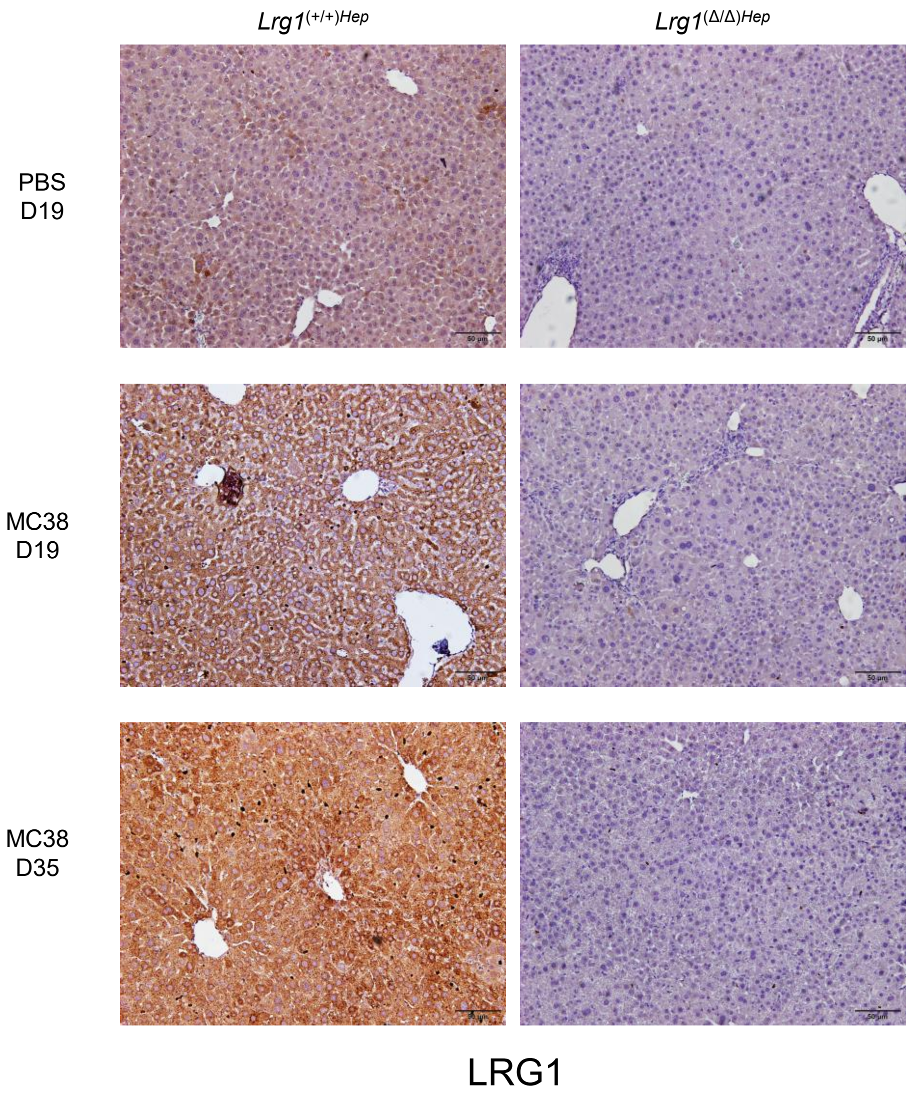

Fig 2L

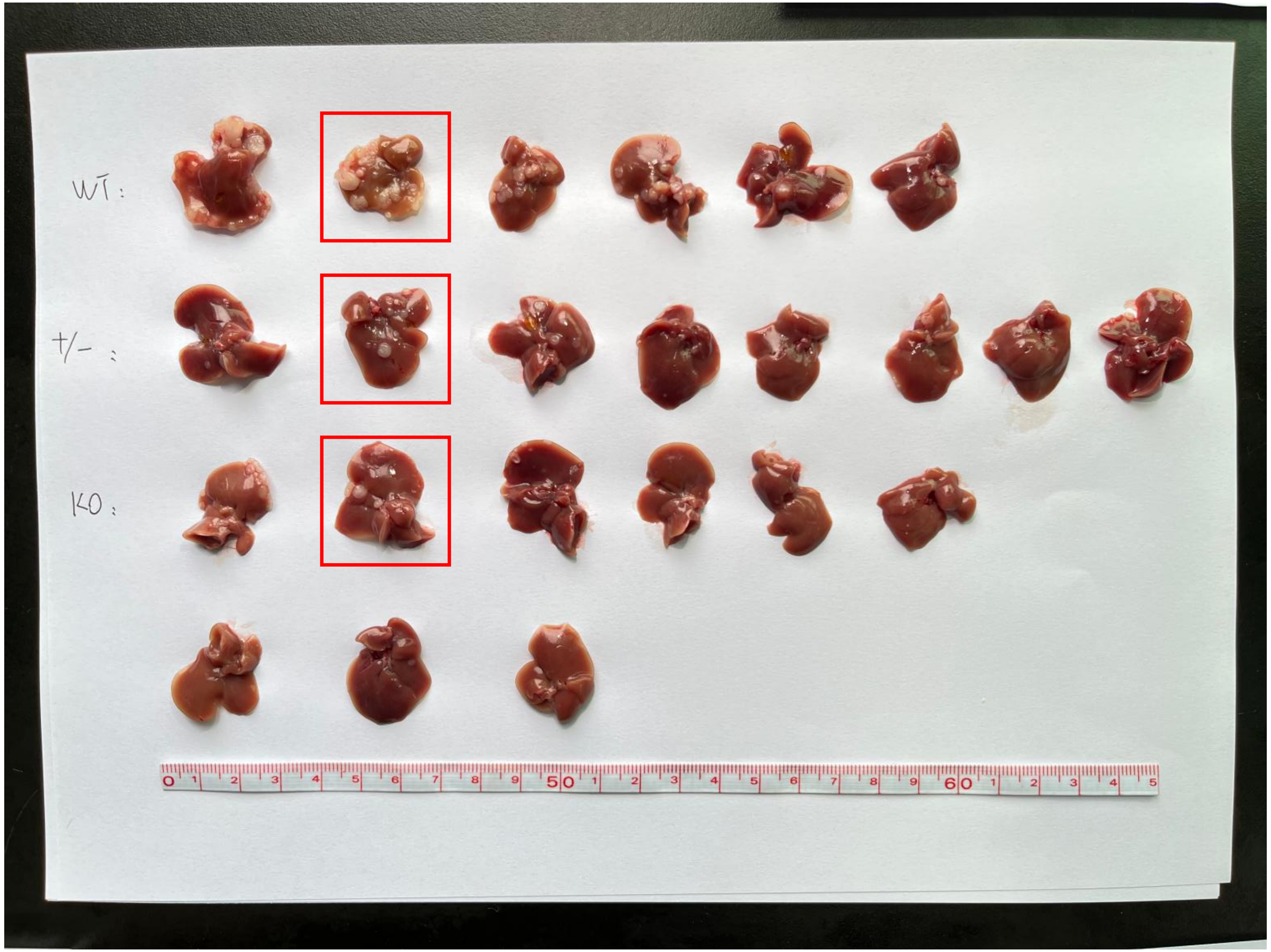

Fig 3A

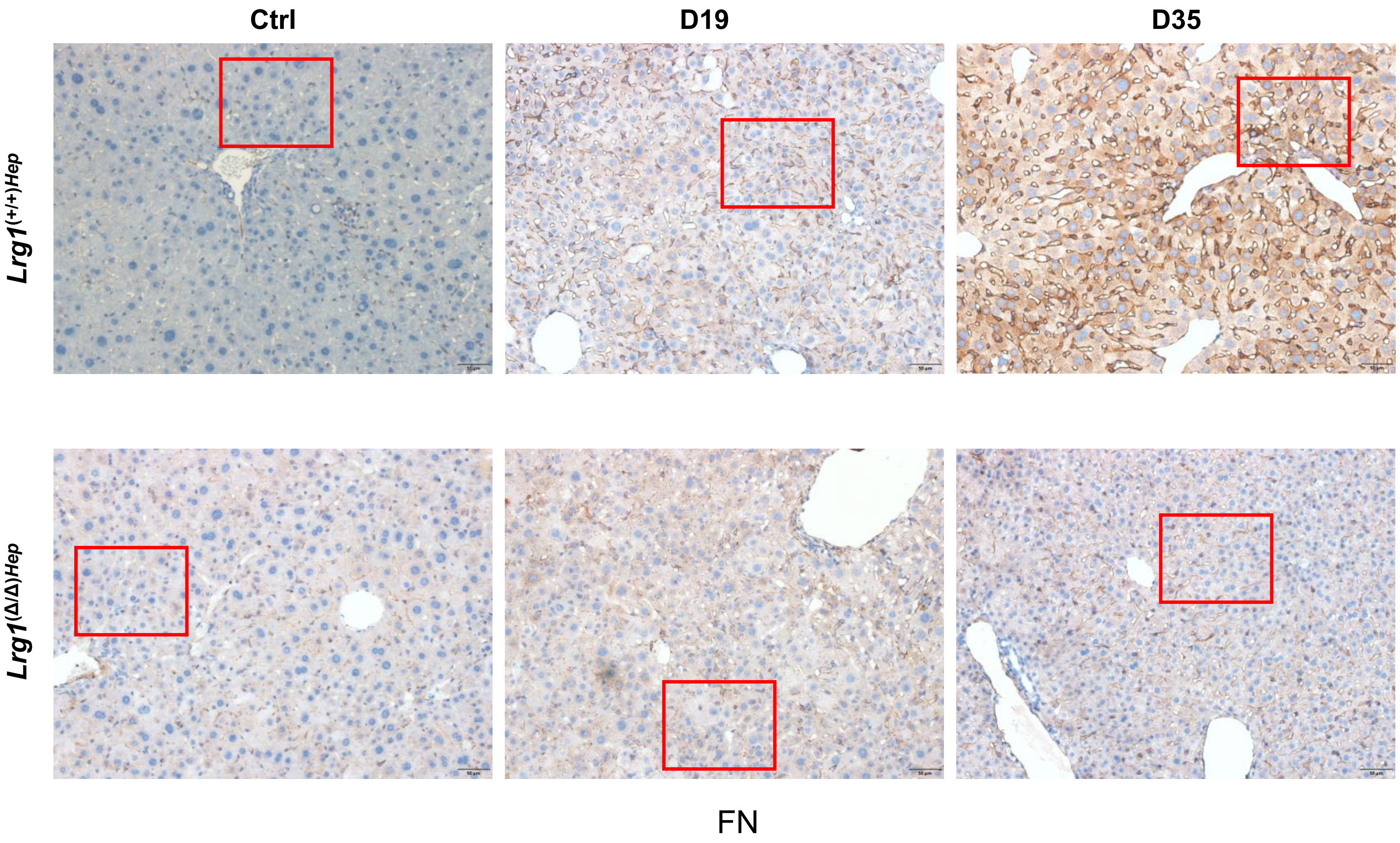

Fig 3B

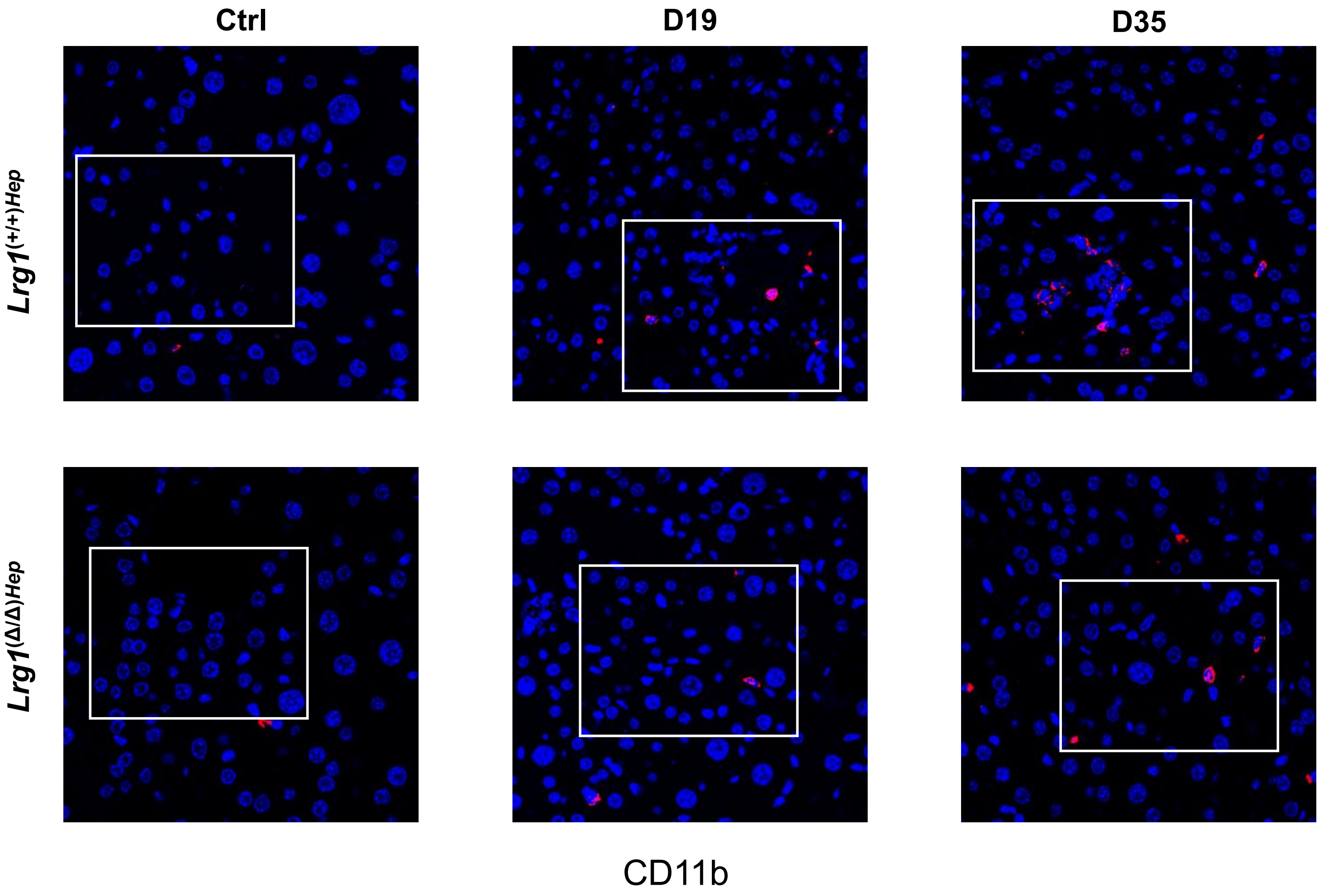

Fig 3D

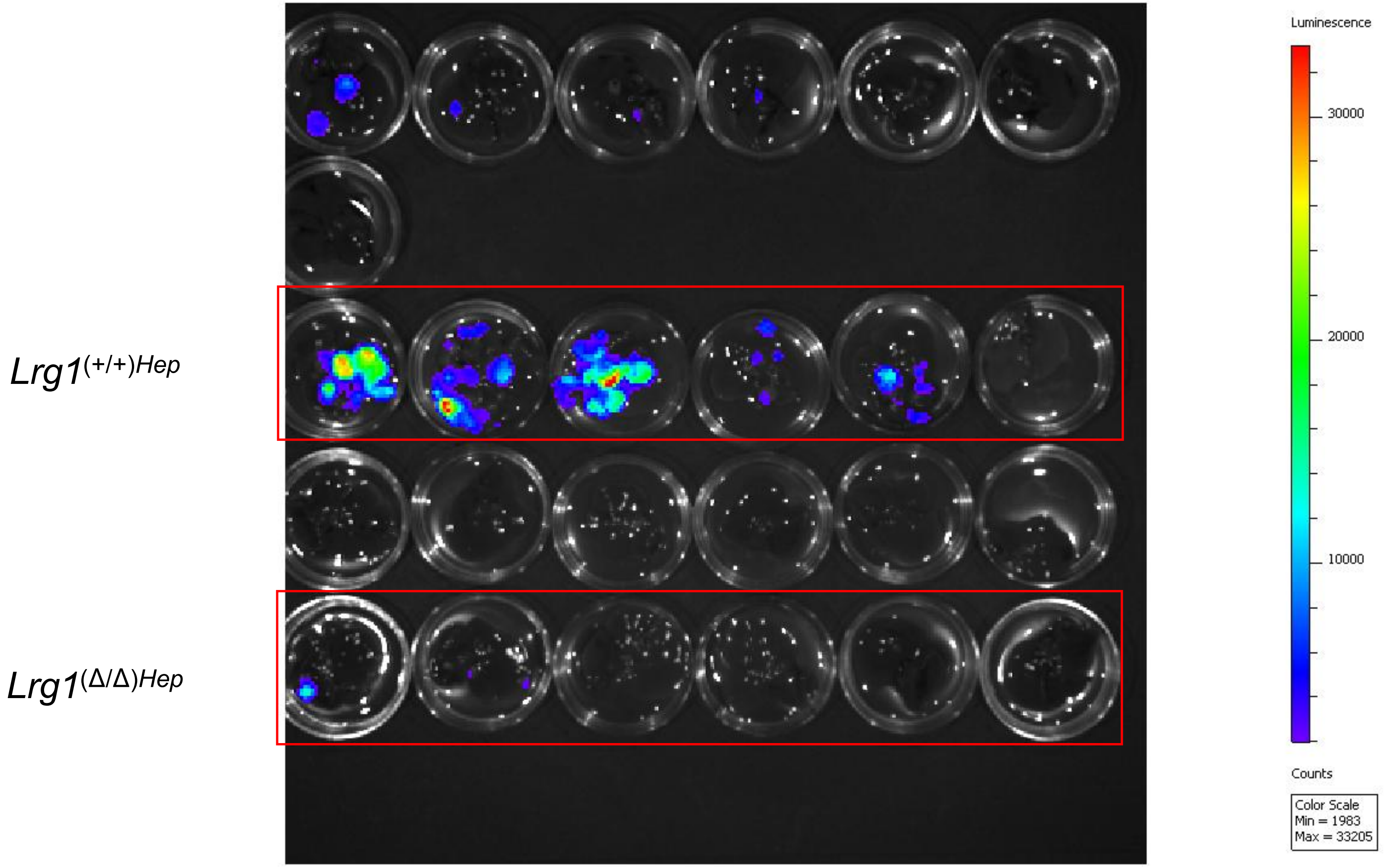

$Lrg1^{(+/+)Hep}$
$Lrg1^{(\Delta/\Delta)Hep}$
Luminescence
30000
20000
10000
Counts
Color Scale
Min = 1983
Max = 33205

Fig 3J

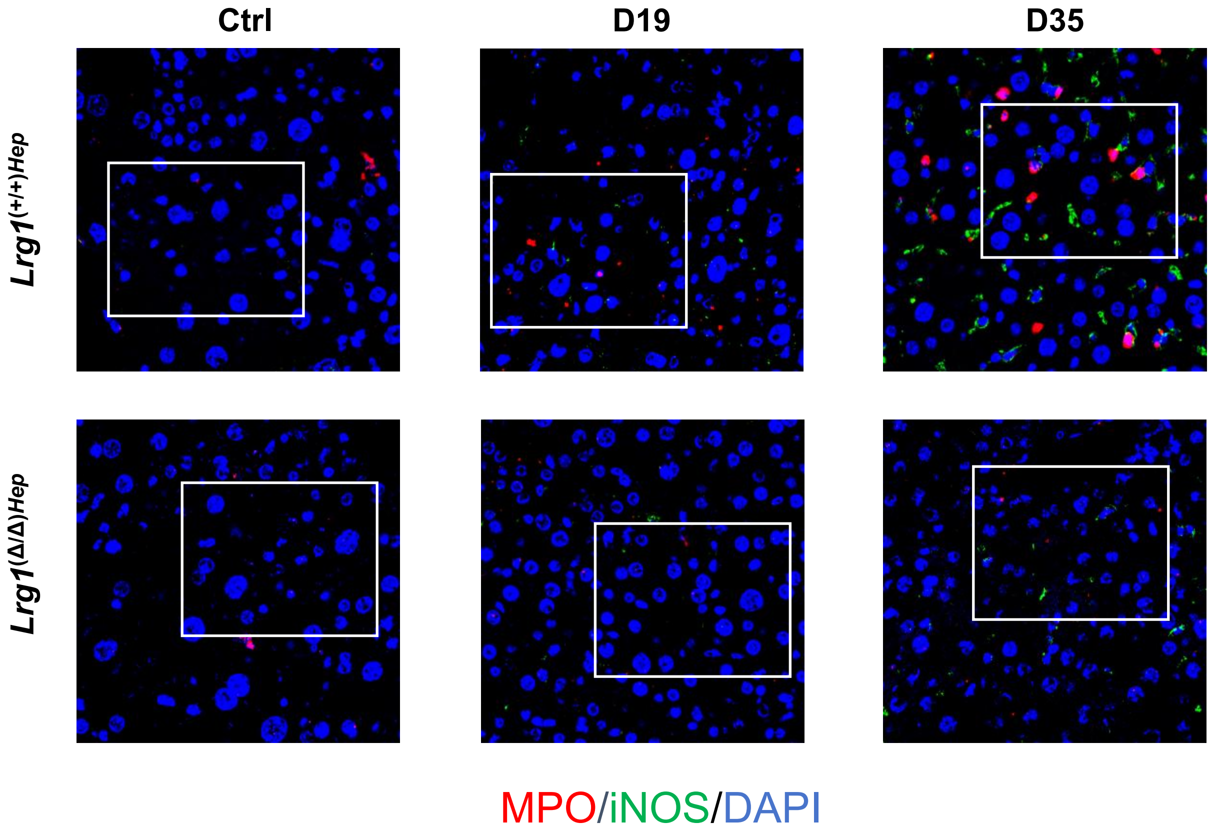

Fig 4A

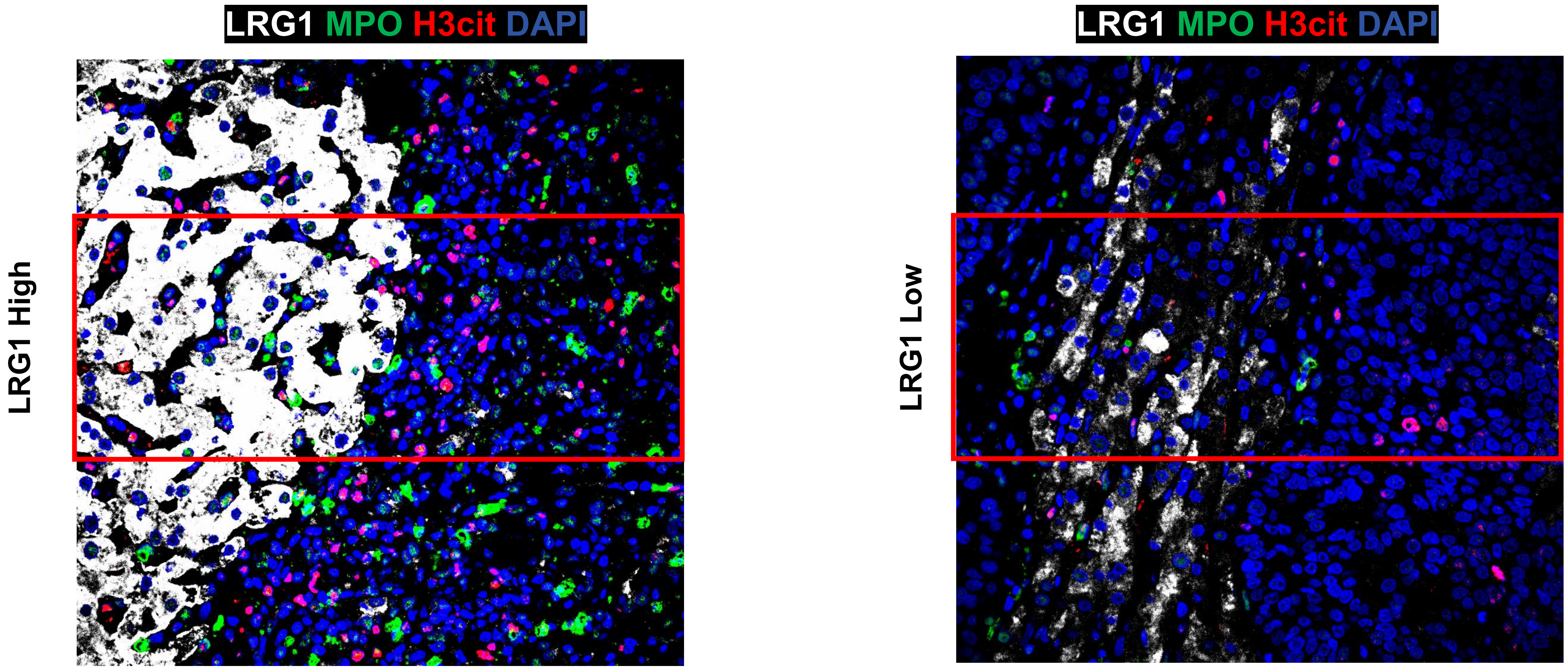

Fig 4B

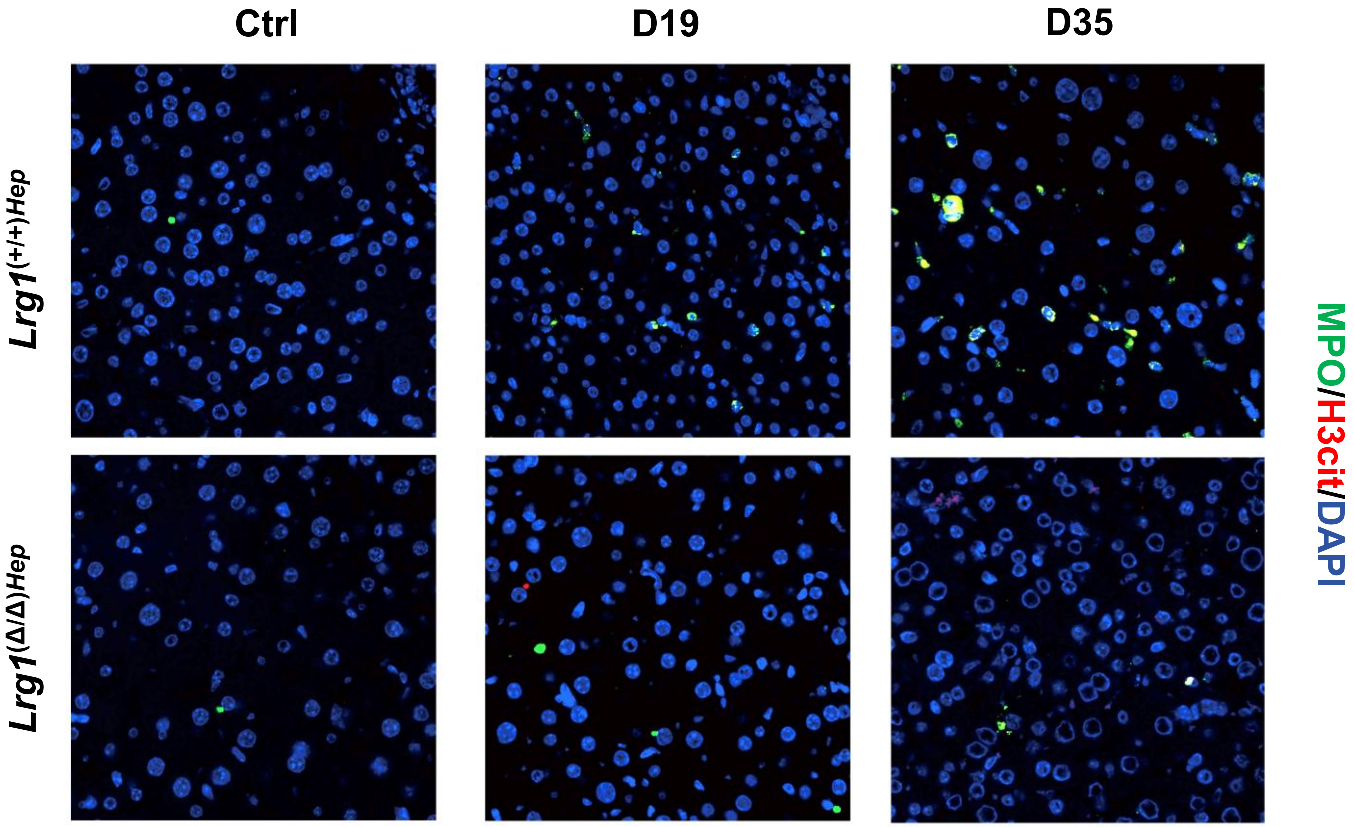

Fig 4C

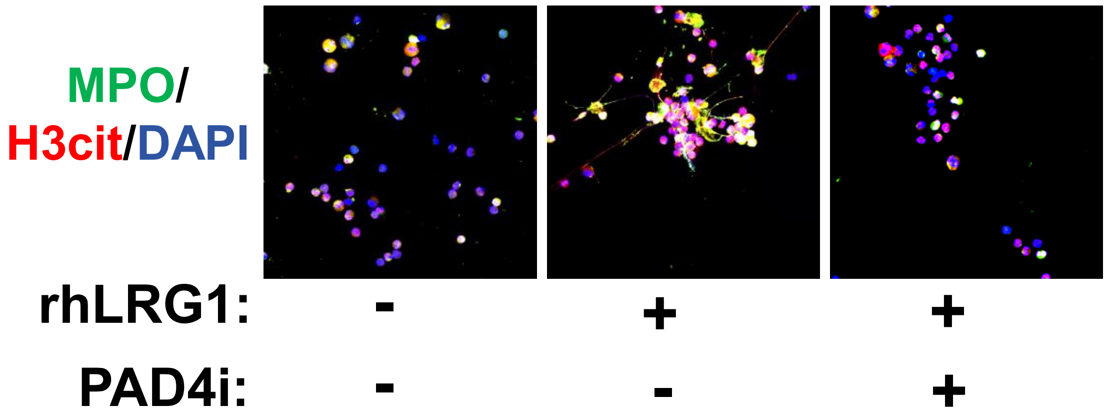
MPO/
H3cit/DAPI
rhLRG1: - + +
PAD4i: - - +

Fig 4F

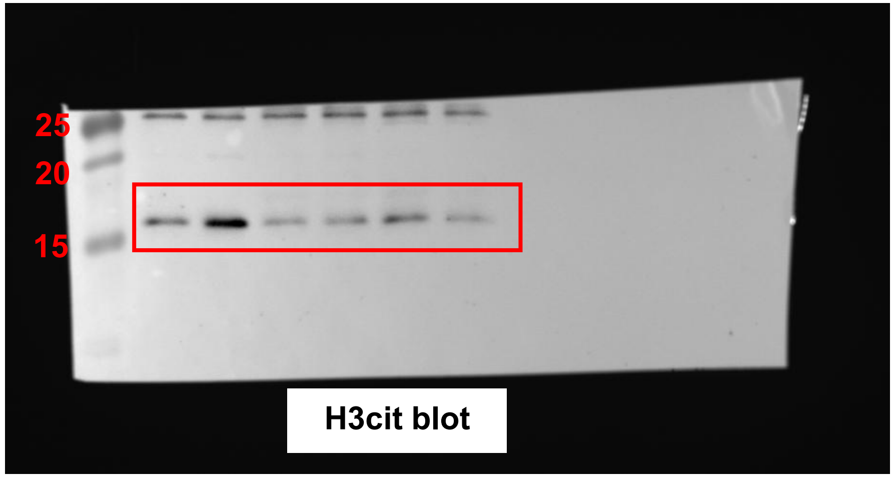
25
20
15
H3cit blot

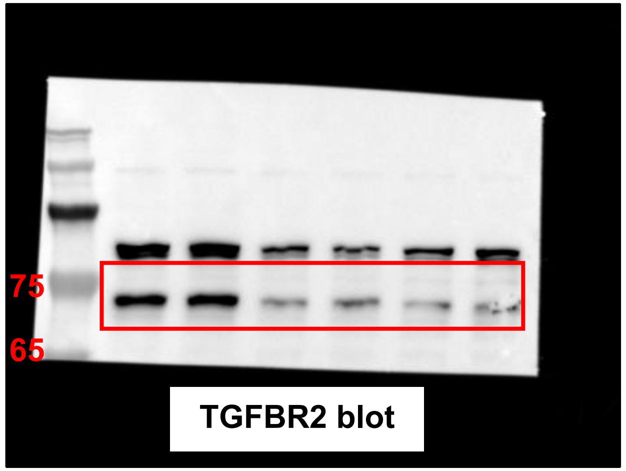
75
65
TGFBR2 blot

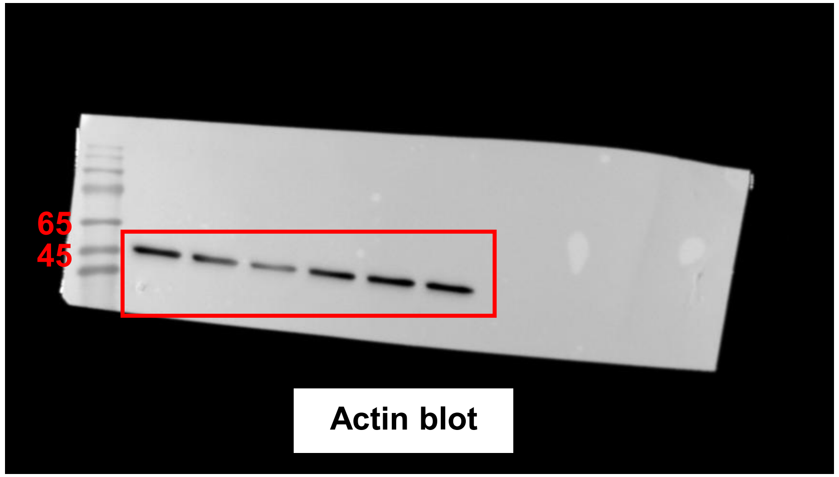
65
45
Actin blot

Fig 4G

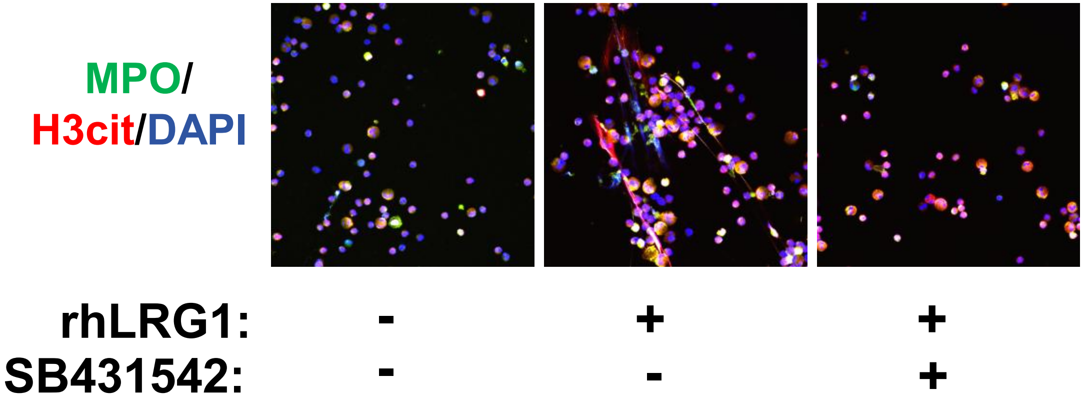

Fig 4H

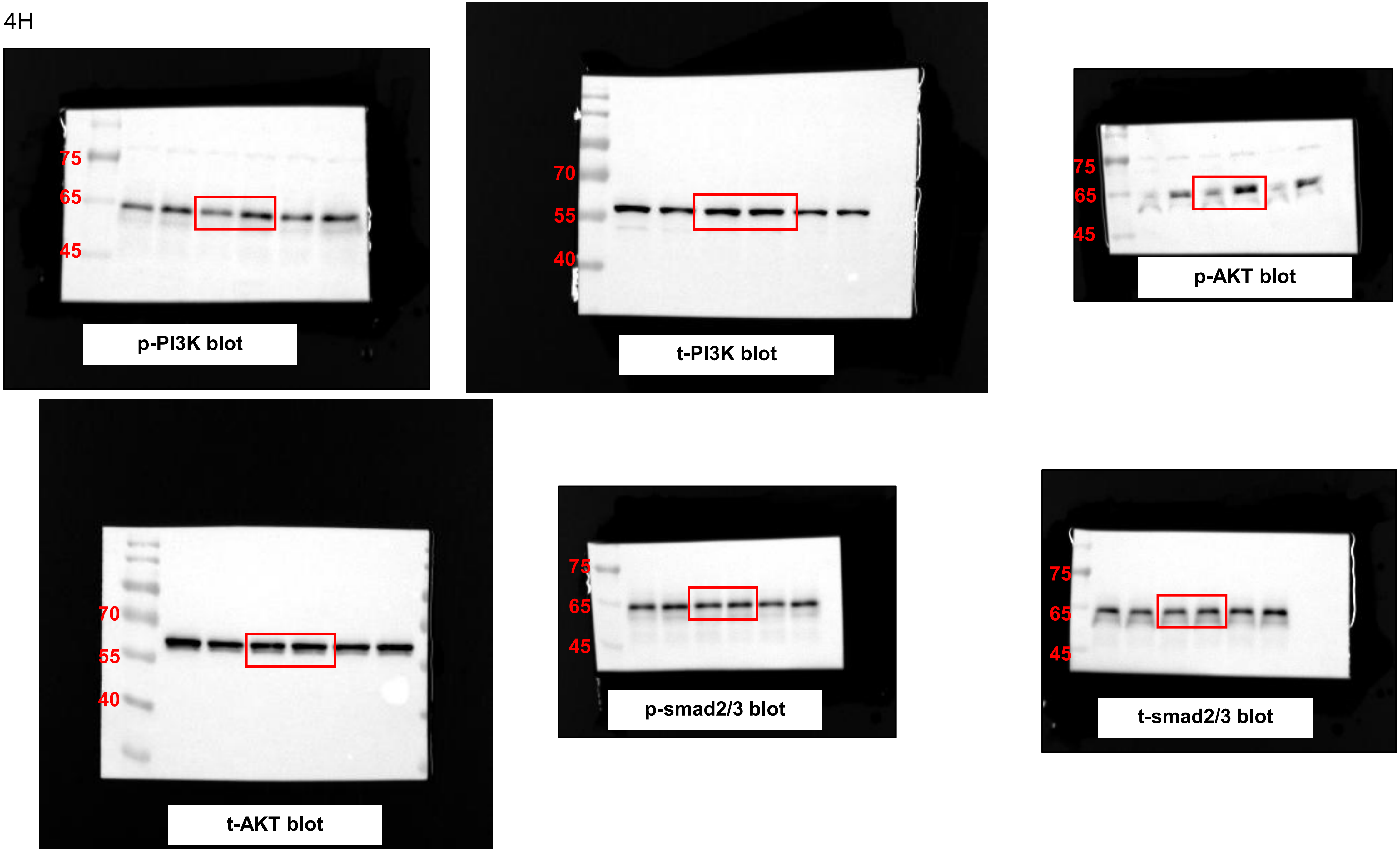
75
65
45
p-PI3K blot
70
55
40
t-PI3K blot
75
65
45
p-AKT blot
70
55
40
t-AKT blot
75
65
45
p-smad2/3 blot
75
65
45
t-smad2/3 blot

Fig 4H

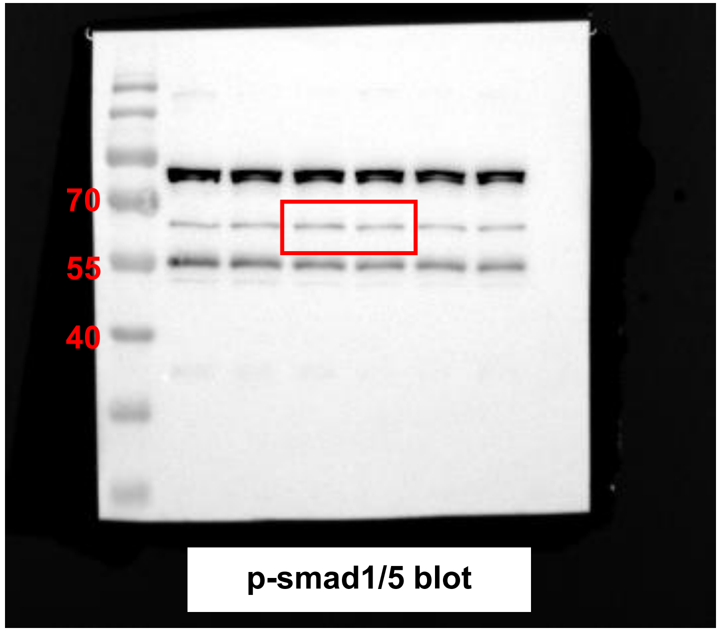

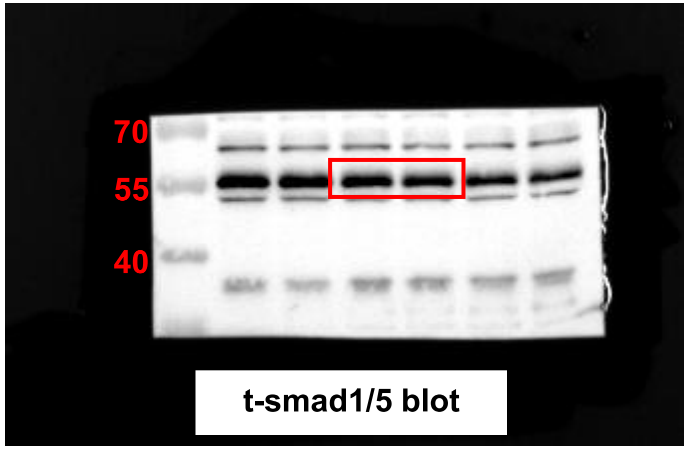

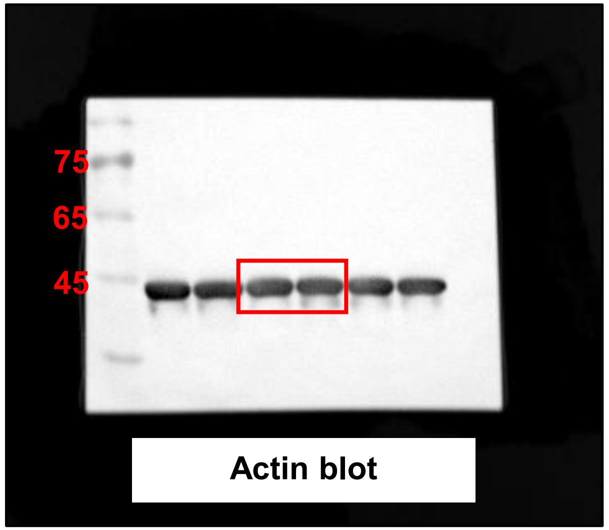

Fig 4I

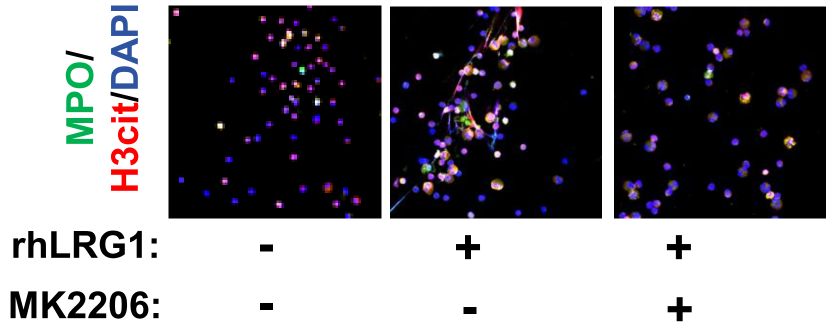
MPO/
H3cit/DAPI
rhLRG1: - + +
MK2206: - - +

Fig 4J

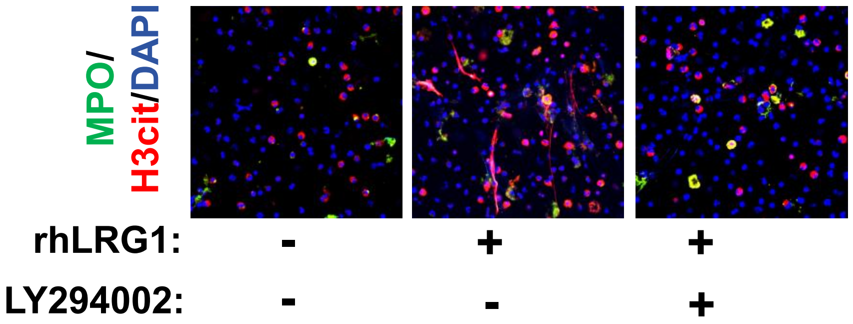
MPO/
H3cit/DAPI
rhLRG1: - + +
LY294002: - - +

Fig 4K

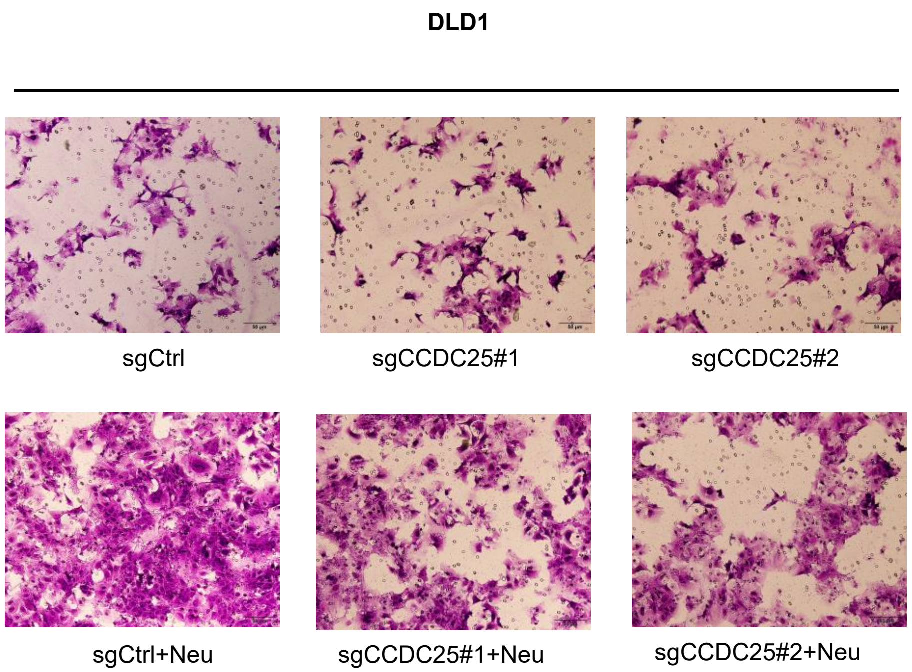
DLD1
sgCtrl
sgCCDC25#1
sgCCDC25#2
sgCtrl+Neu
sgCCDC25#1+Neu
sgCCDC25#2+Neu

Fig 4M

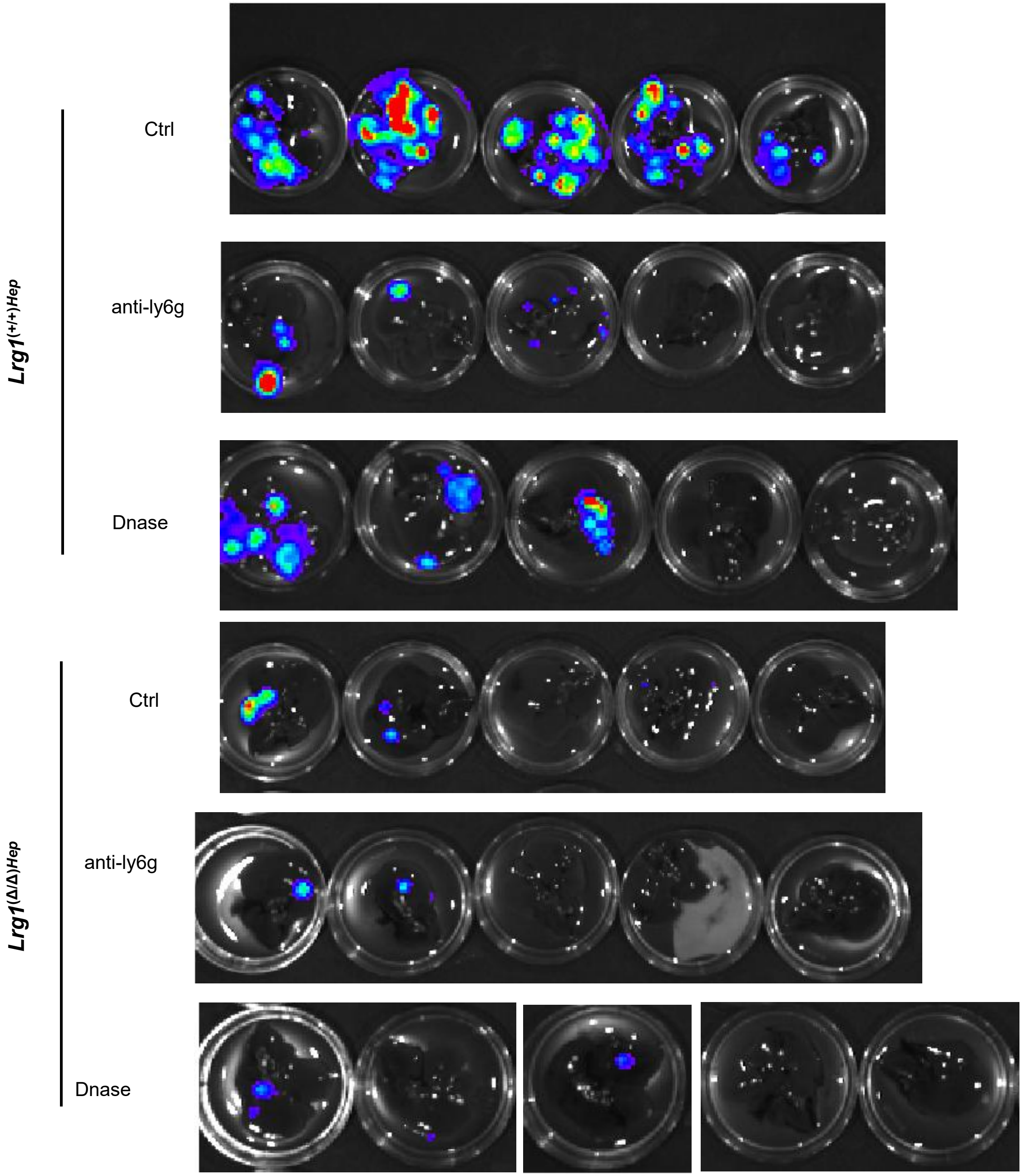

Ctrl
anti-ly6g
Dnase
$Lrg1^{(+/+)Hep}$
Ctrl
anti-ly6g
Dnase
$Lrg1^{(\Delta/\Delta)Hep}$

Fig 5B

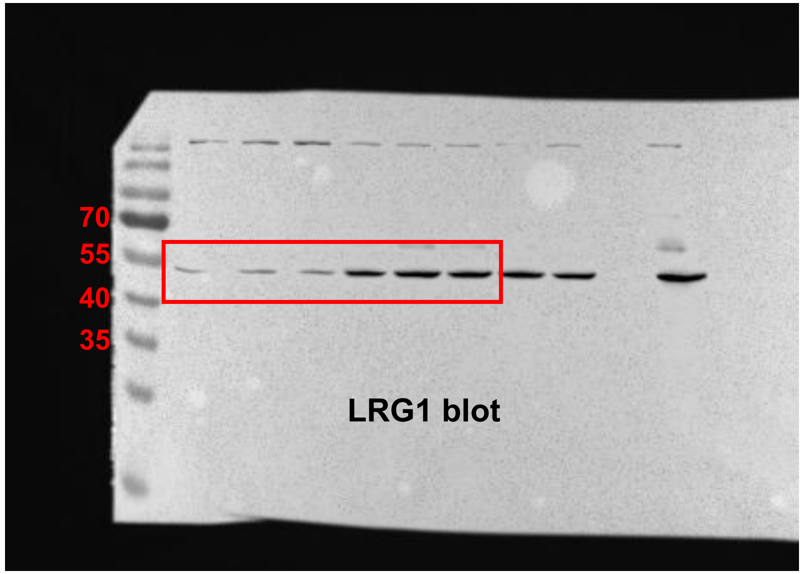

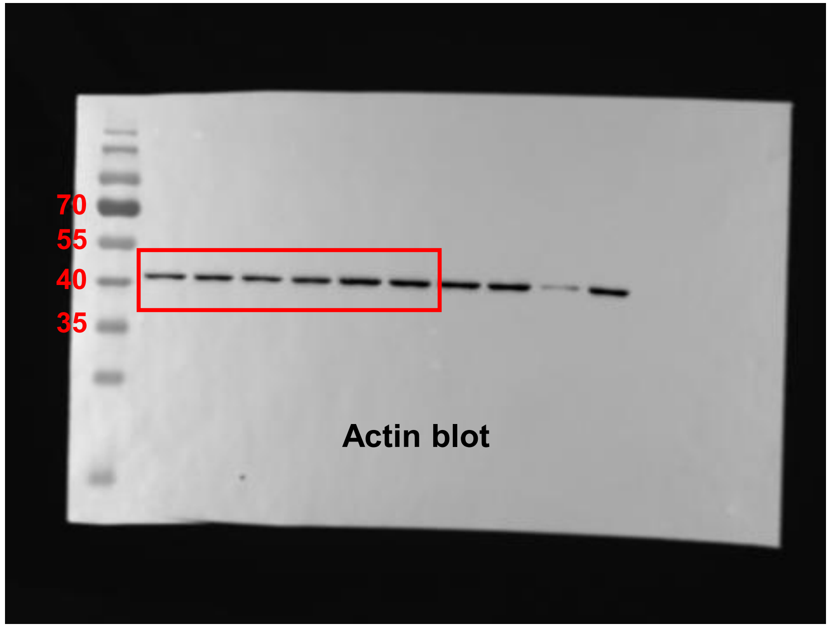

Fig 5G

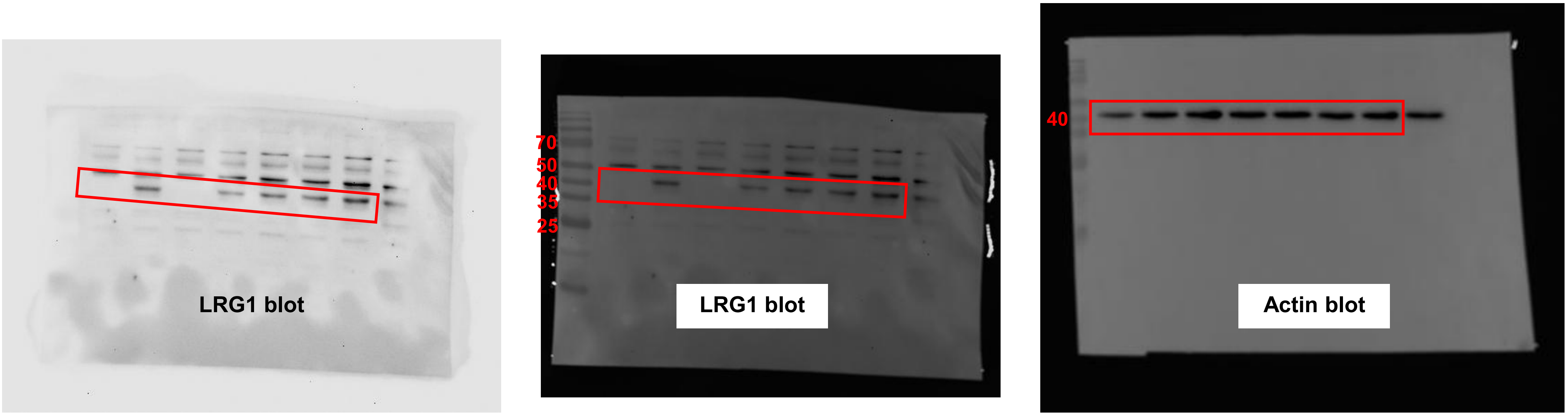

Fig 5I

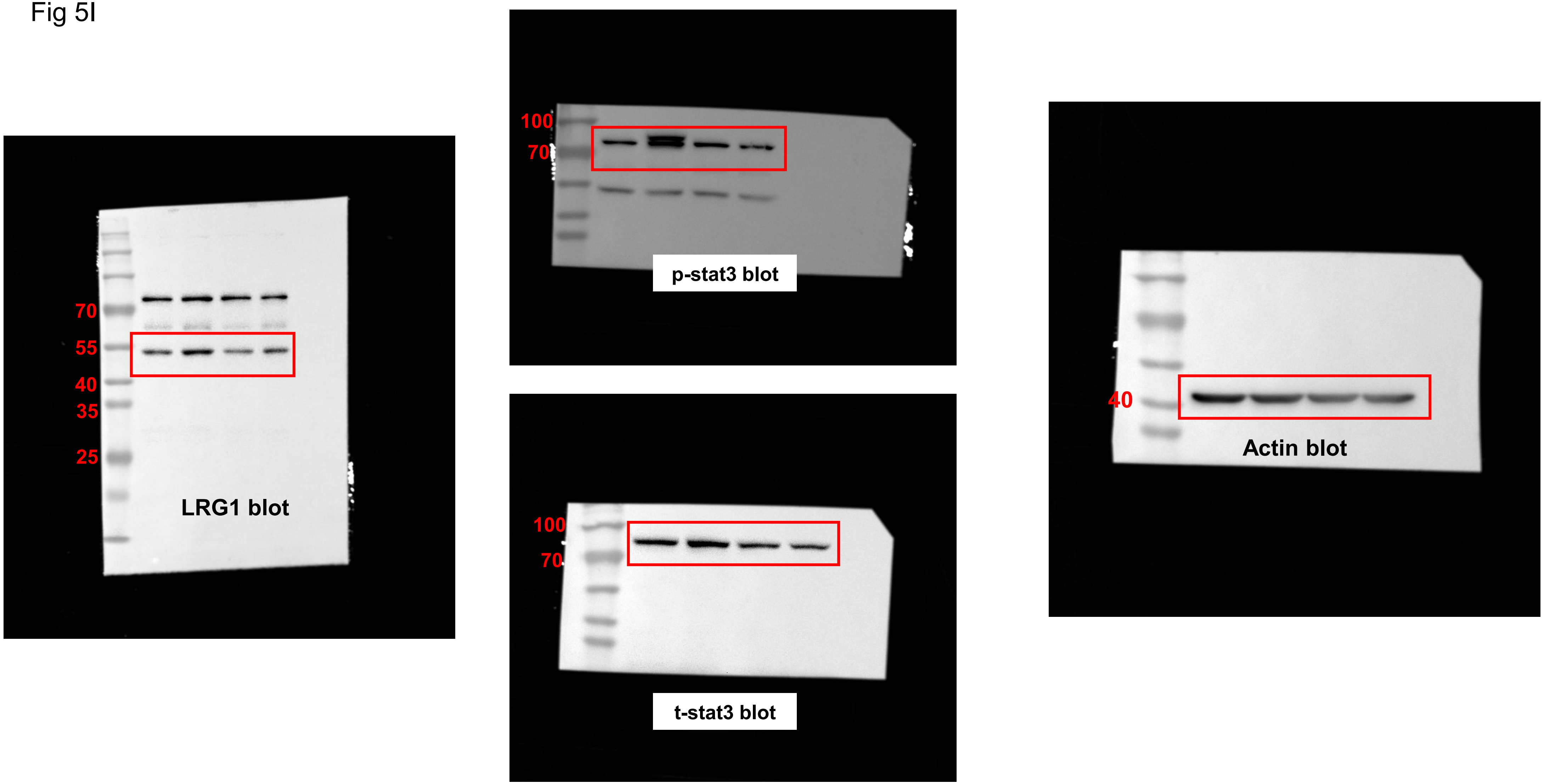

70
55
40
35
25
LRG1 blot
100
70
p-stat3 blot
100
70
t-stat3 blot
40
Actin blot

Fig 5M

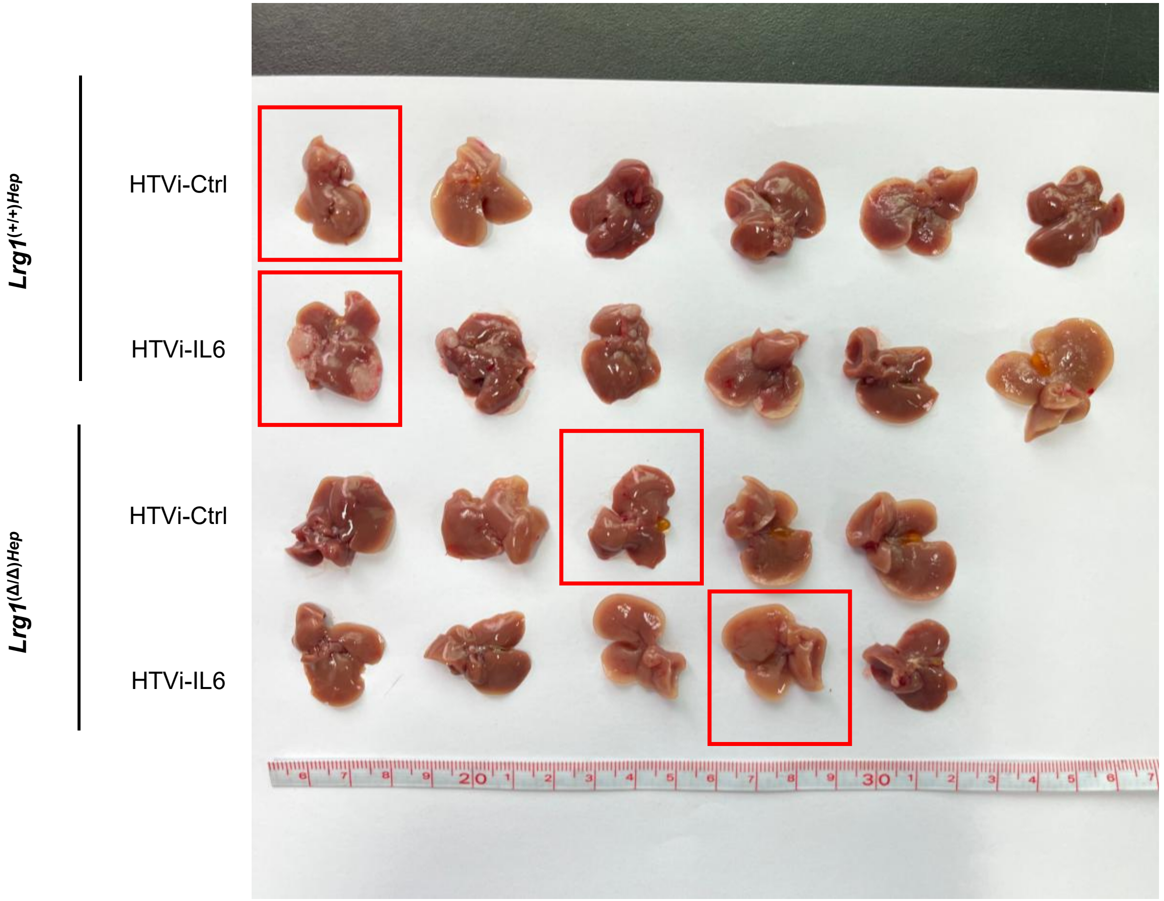

Fig 5N

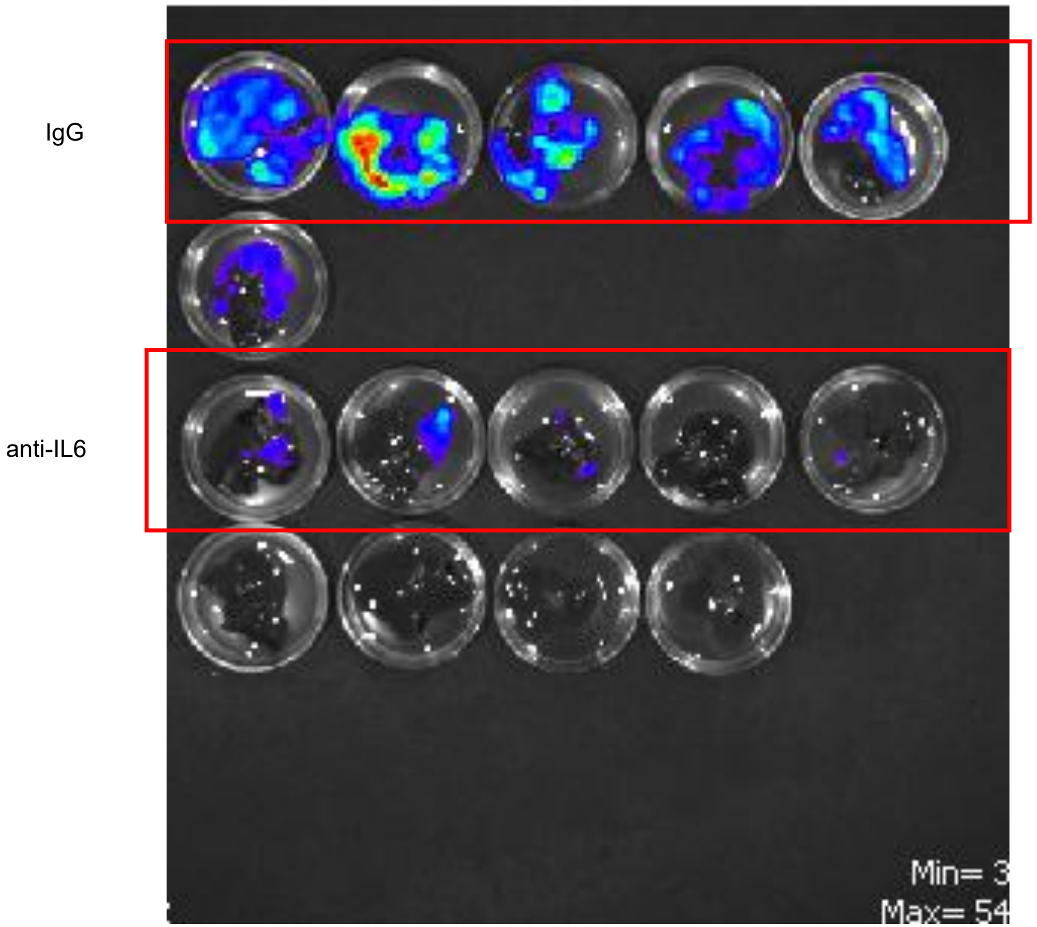

IgG
anti-IL6
Min= 3
Max= 54

Fig 5Q

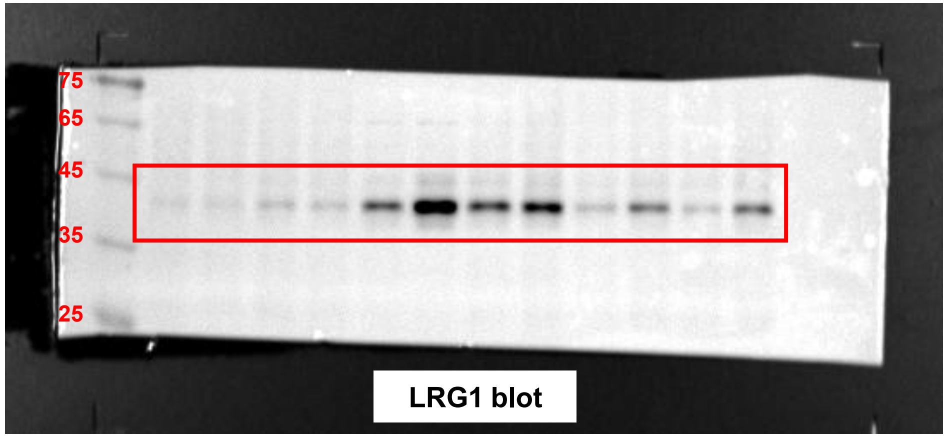

75
65
45
35
25
LRG1 blot

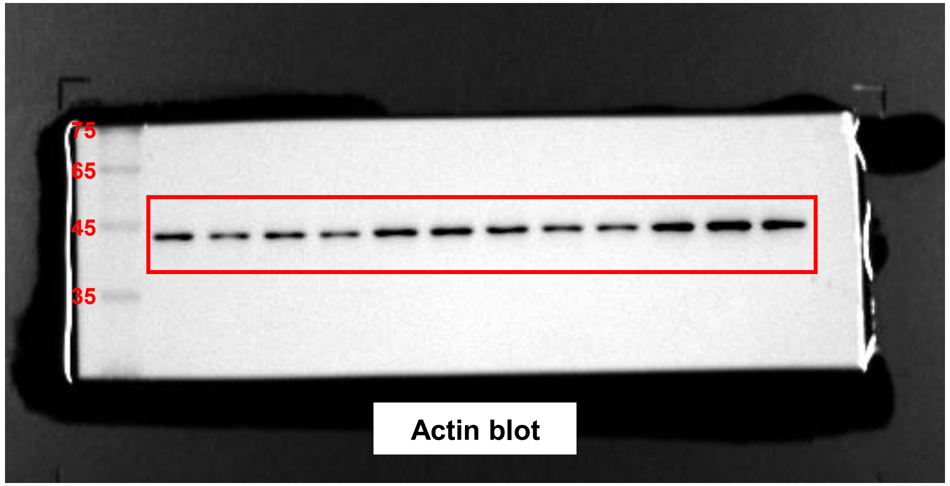

75
65
45
35
Actin blot

Fig 6B

Ctrl

anti-
LRG1

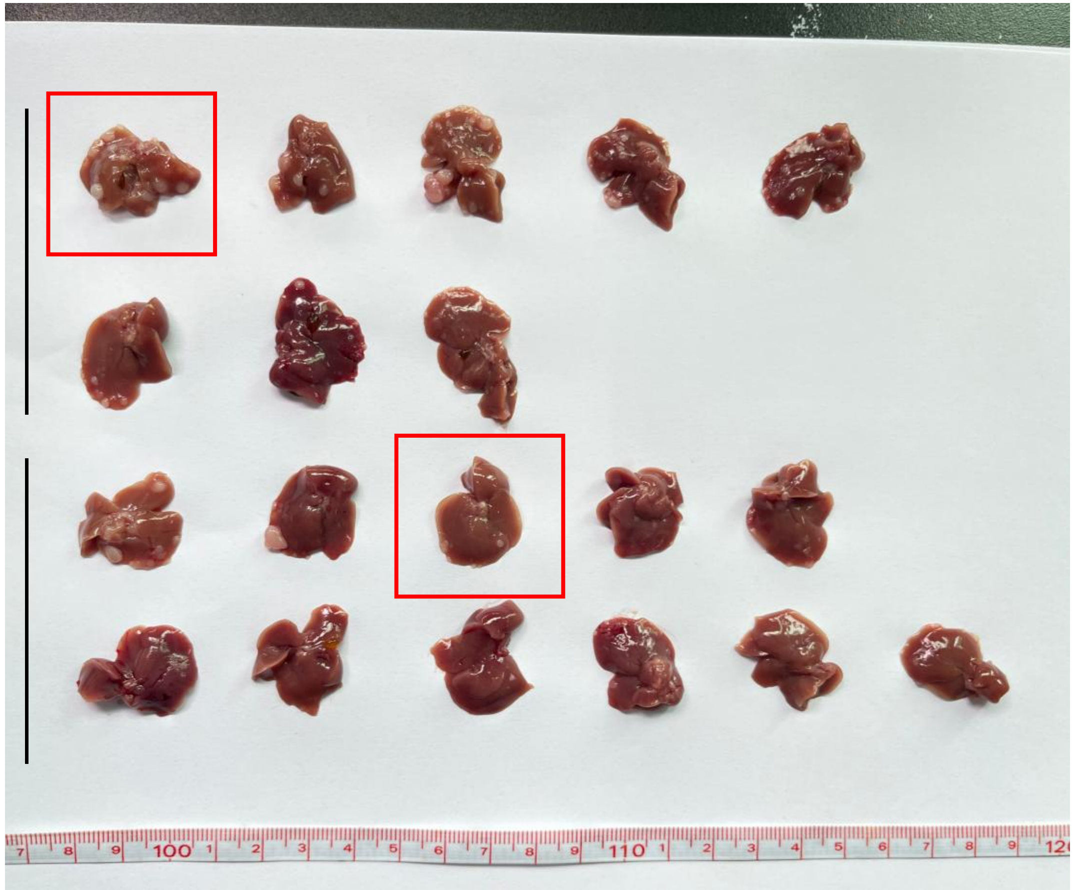

Fig 6F

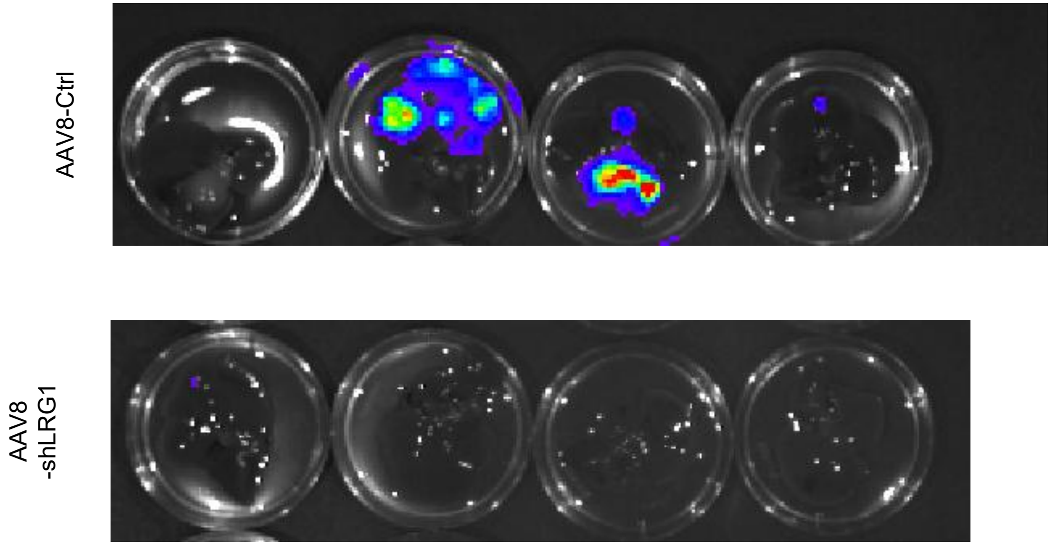

Fig 6I

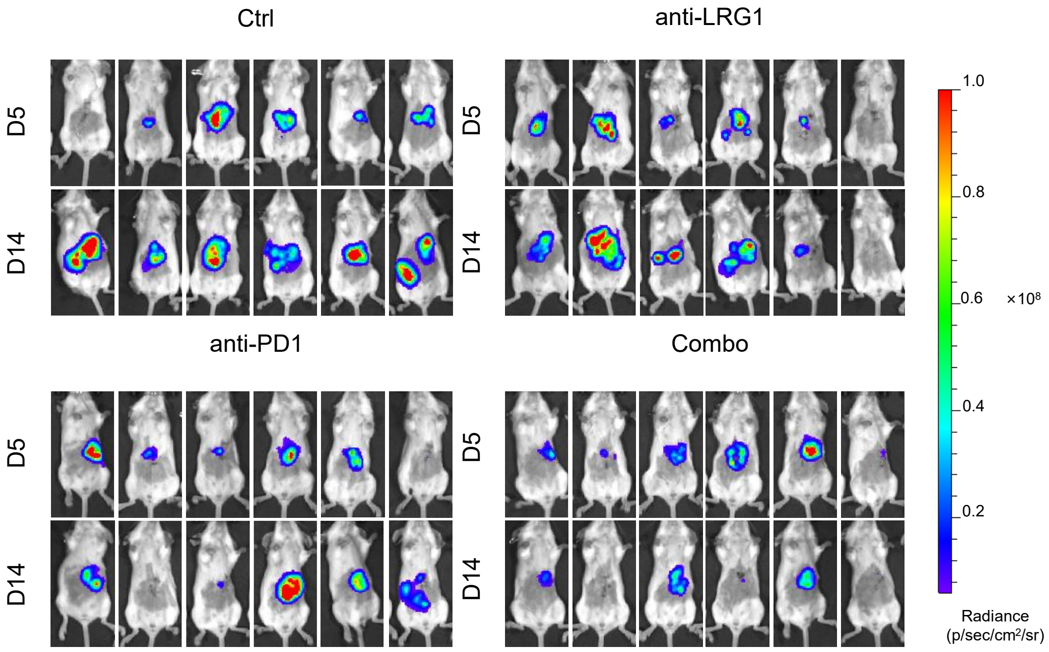

Ctrl
anti-LRG1
anti-PD1
Combo
D5
D14
D5
D14
D5
D14
D5
D14
1.0
0.8
0.6
0.4
0.2
×10^8
Radiance
(p/sec/cm²/sr)

Fig 6K

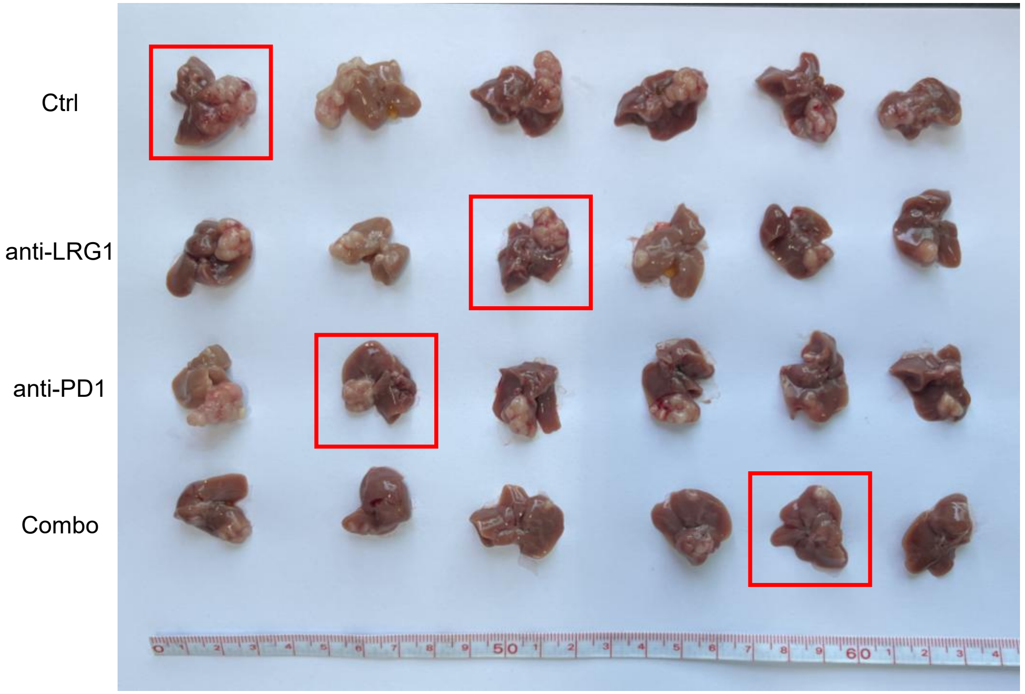

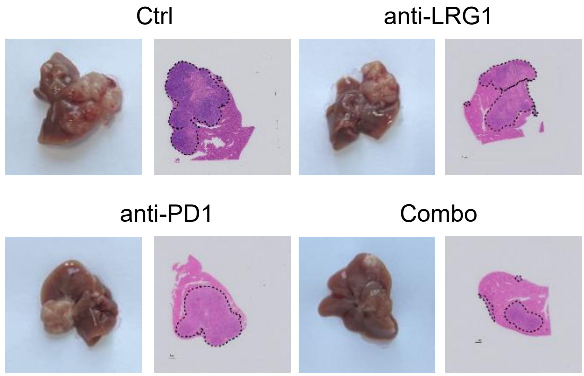

Fig 6M

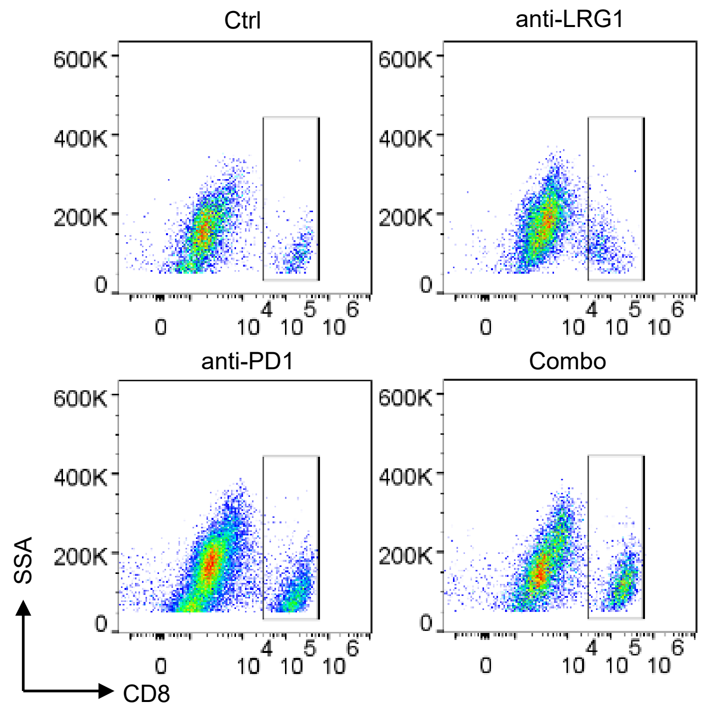

Ctrl
anti-LRG1
anti-PD1
Combo
600K
400K
200K
0
SSA
CD8

Fig 6O

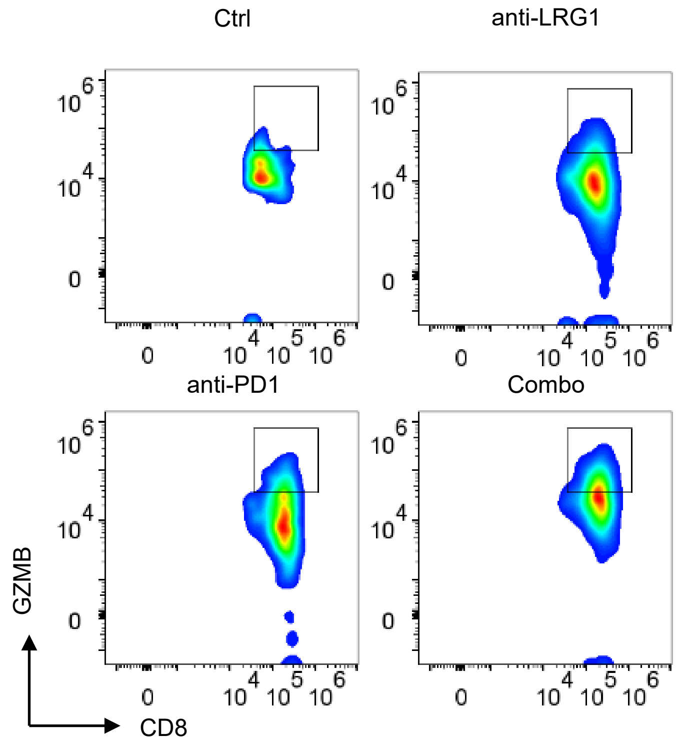

Ctrl
anti-LRG1
anti-PD1
Combo
GZMB
CD8

Fig S1A

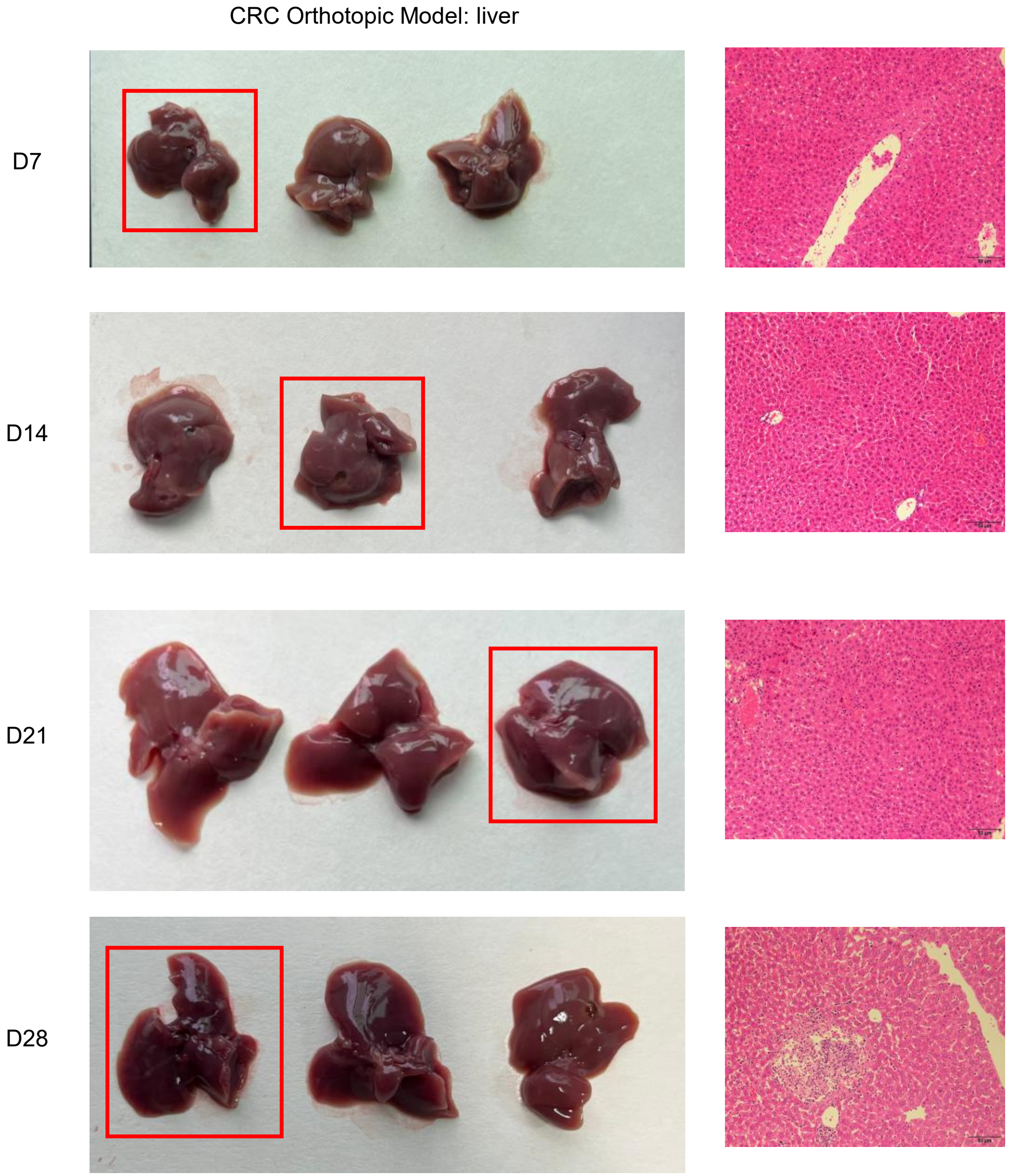

Fig S1B

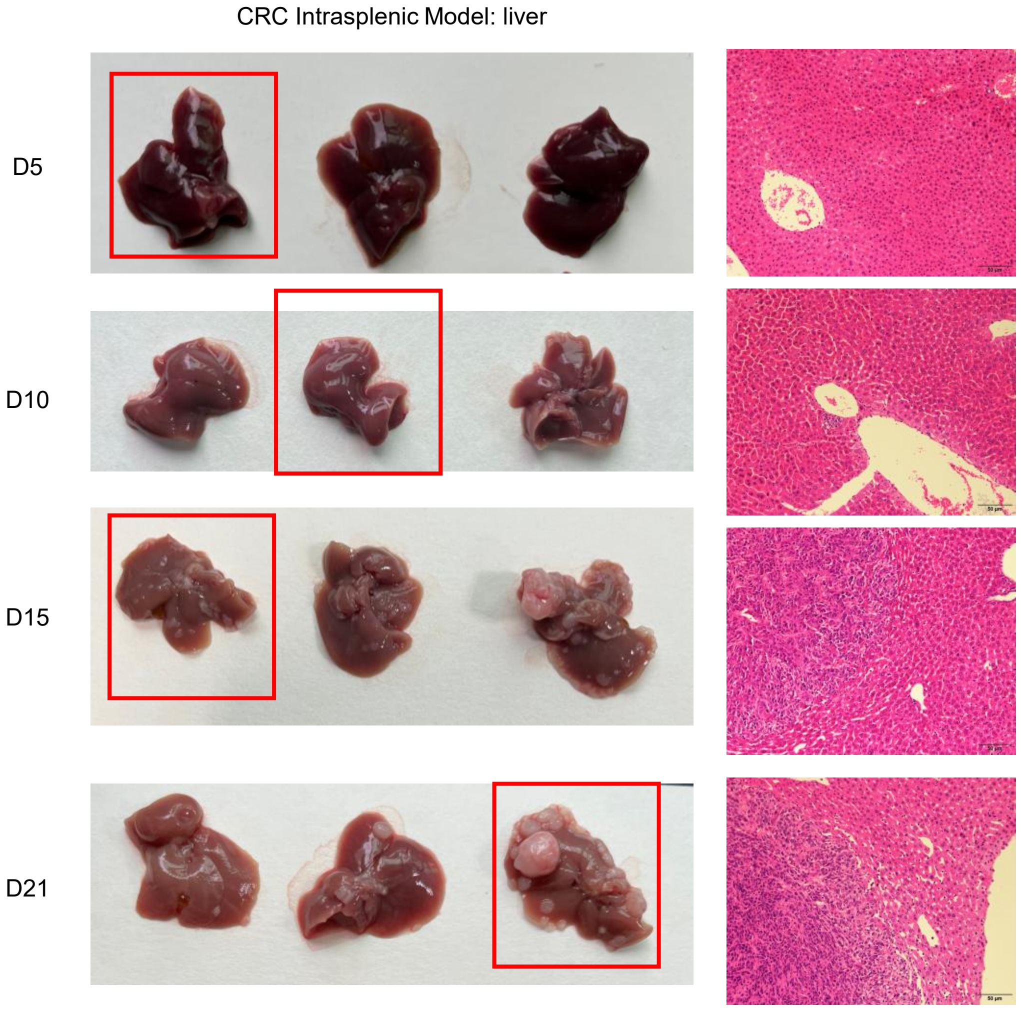

Fig S2A

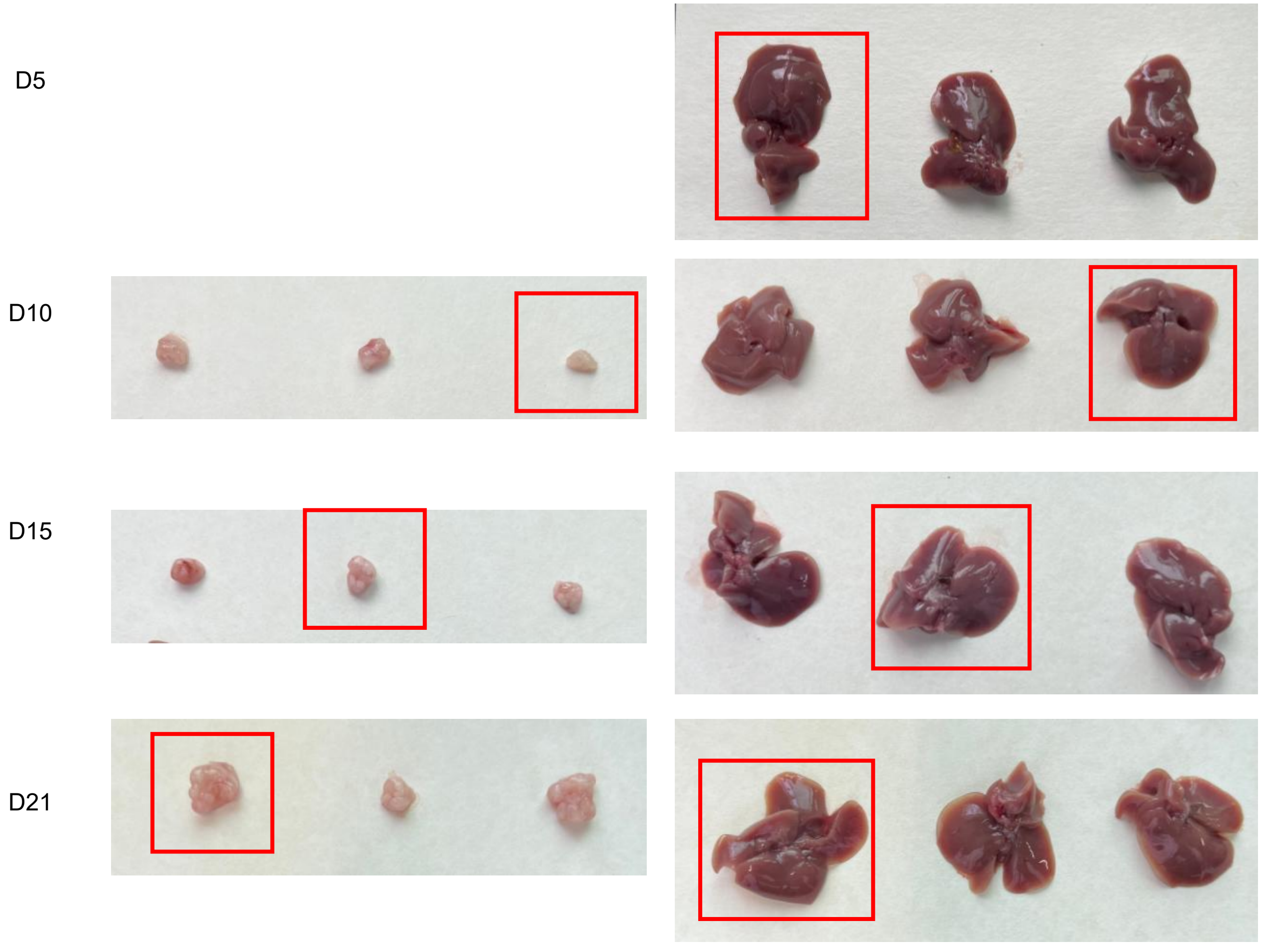

Fig S2A

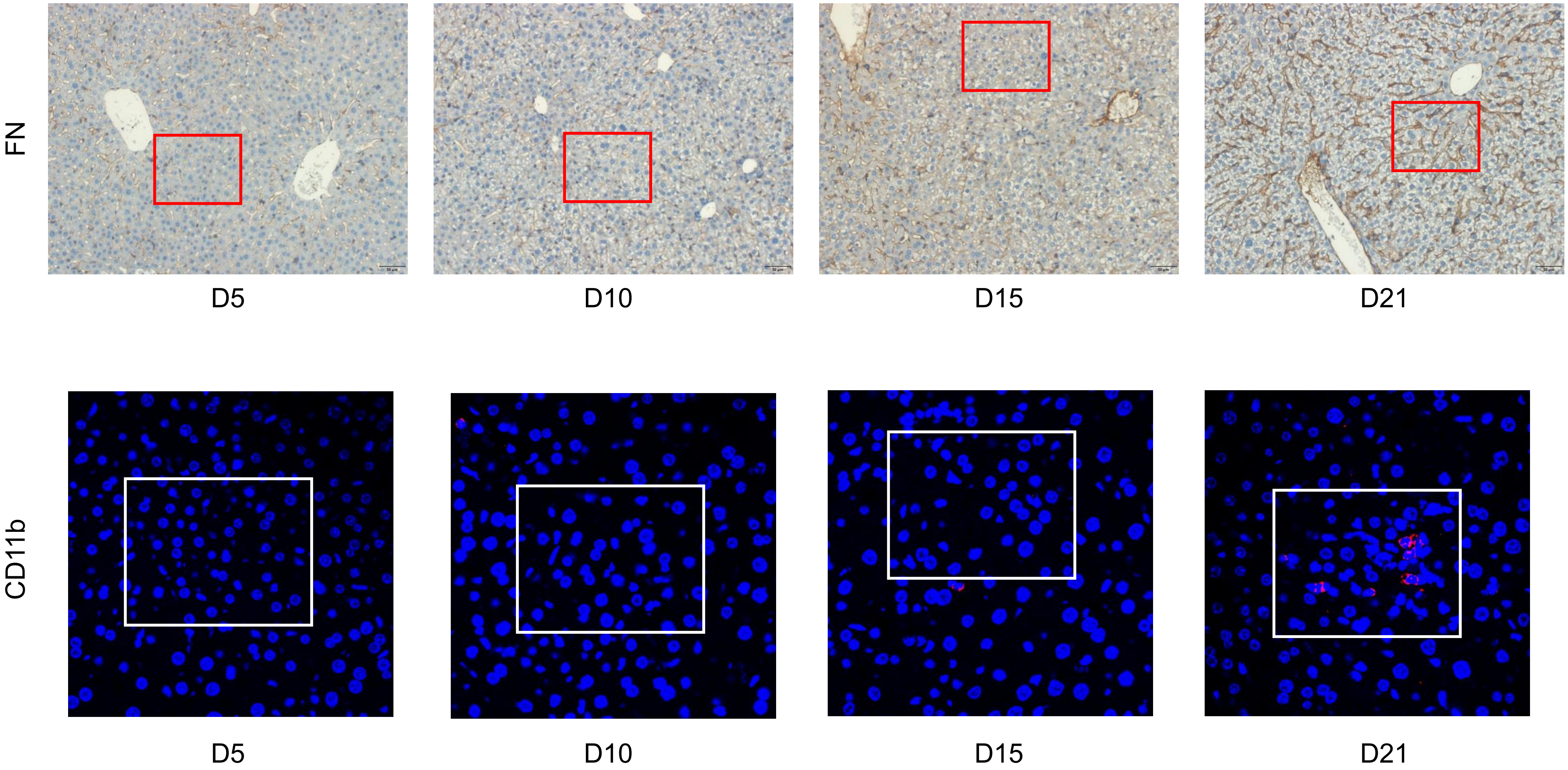

Fig S2D

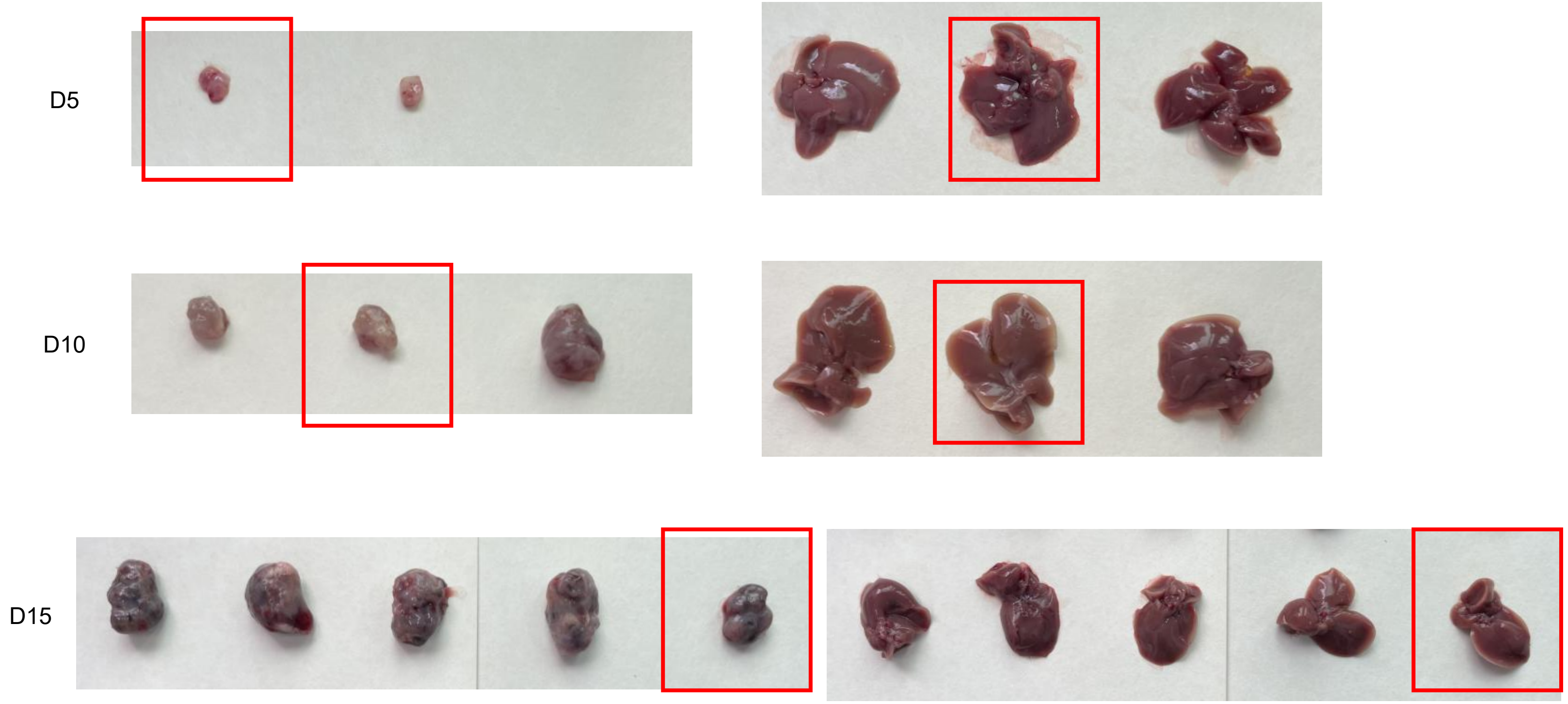

Fig S2D

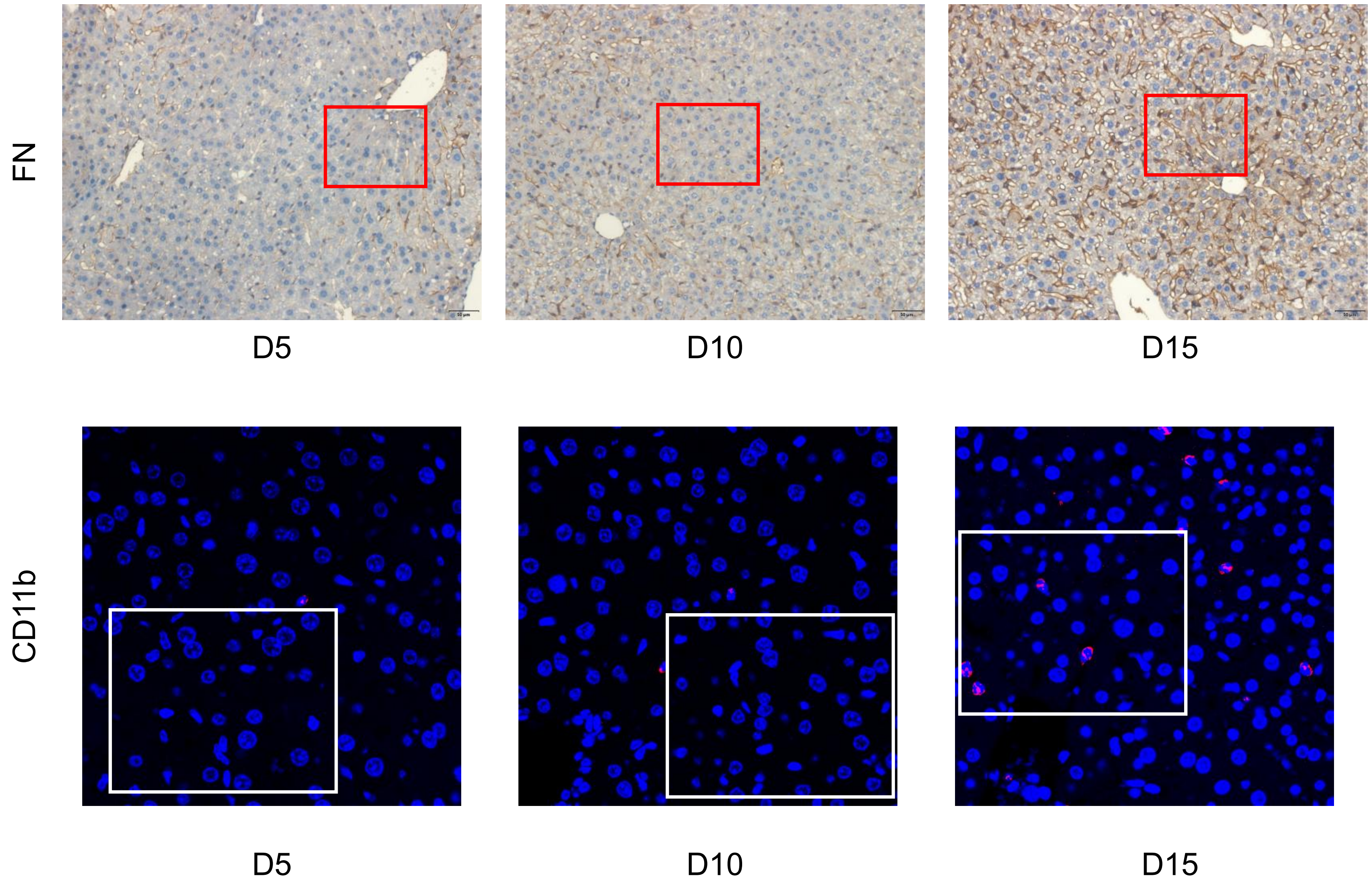

Fig S2G

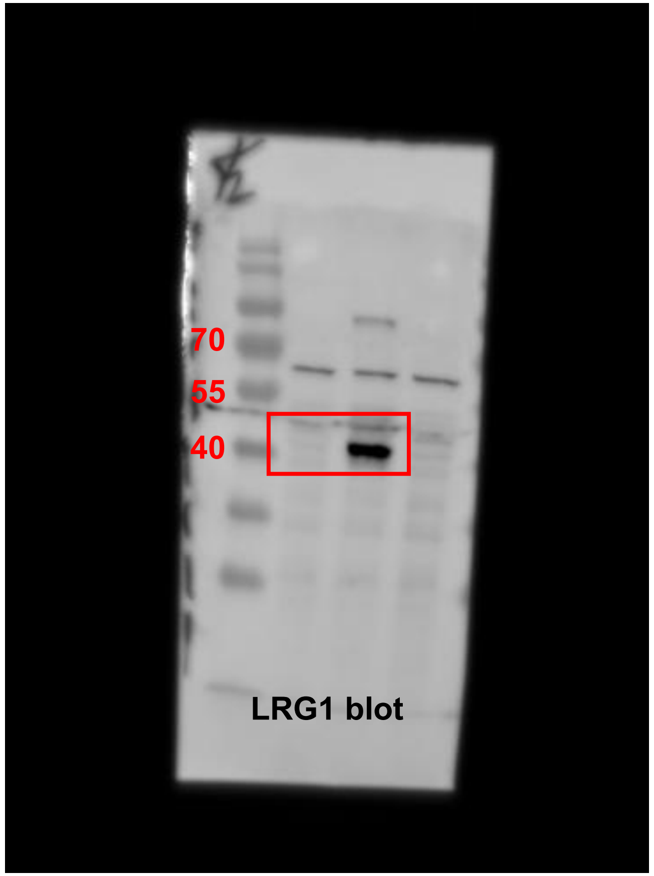

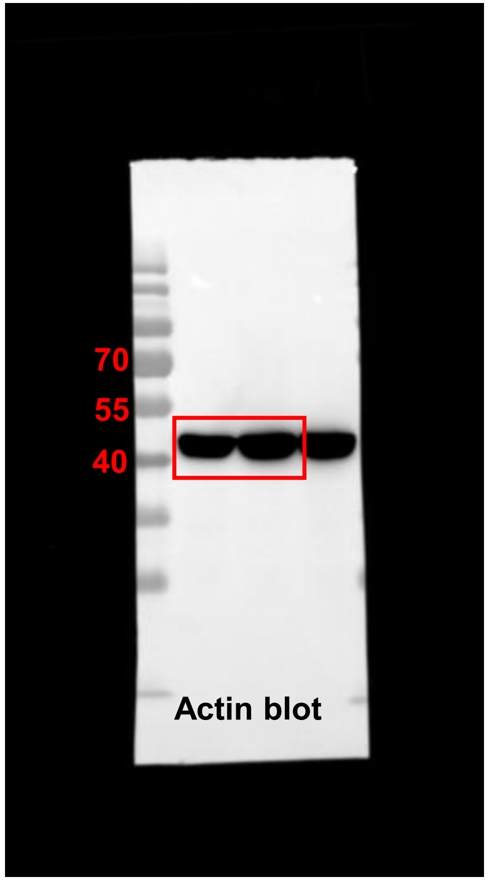

Fig S2H

HTVi-Ctrl

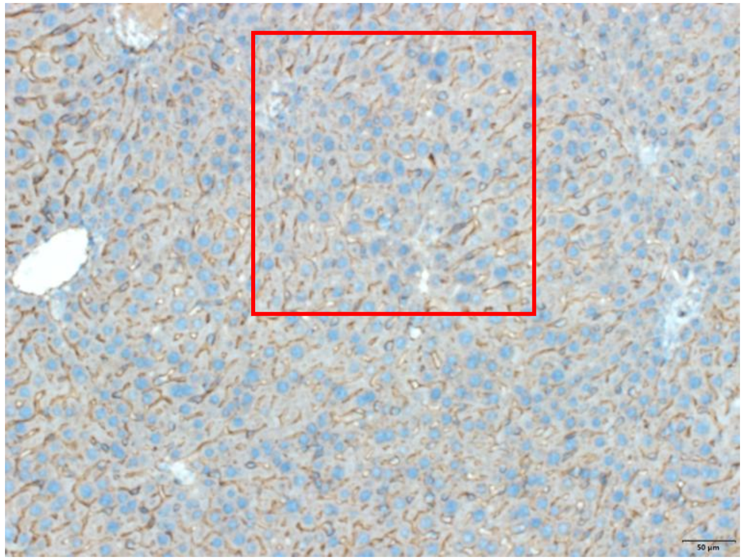

HTVi-mLRG1

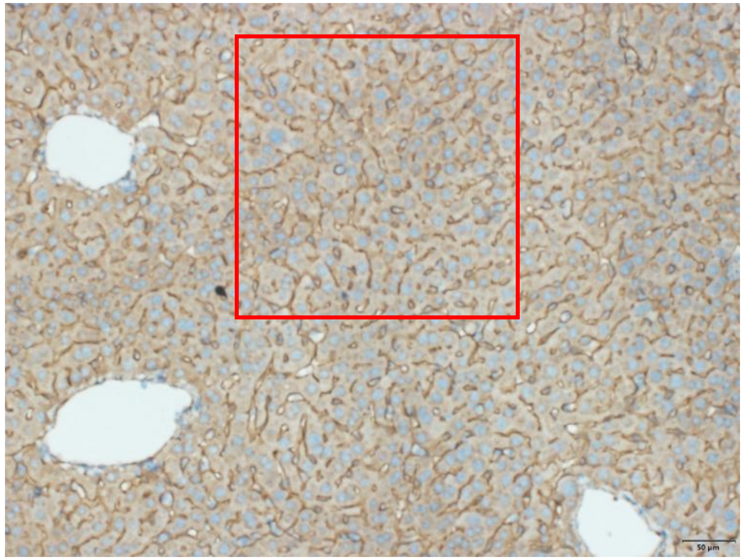

Fig S3A

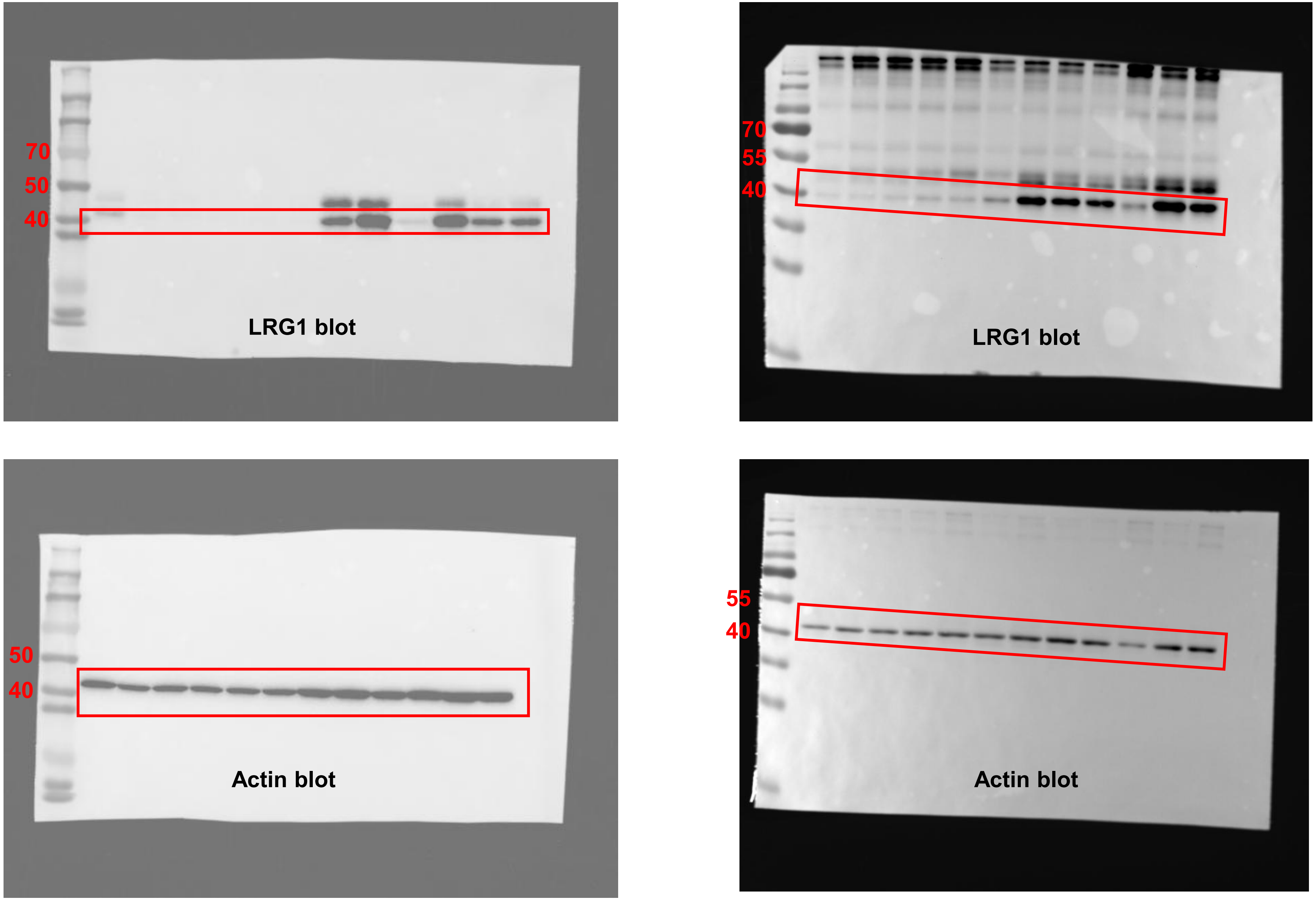
70
50
40
LRG1 blot
70
55
40
LRG1 blot
50
40
Actin blot
55
40
Actin blot

Fig S3E

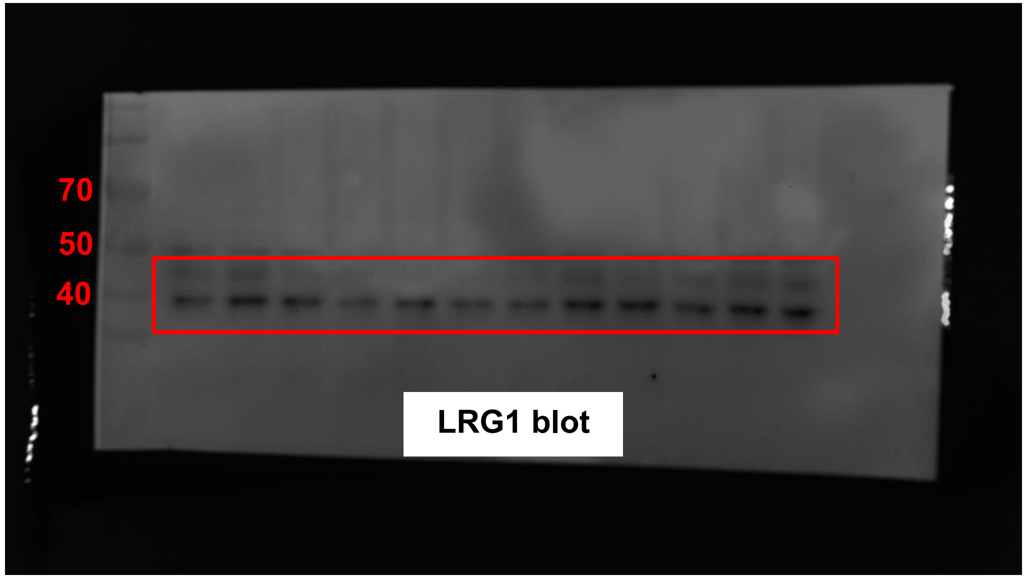

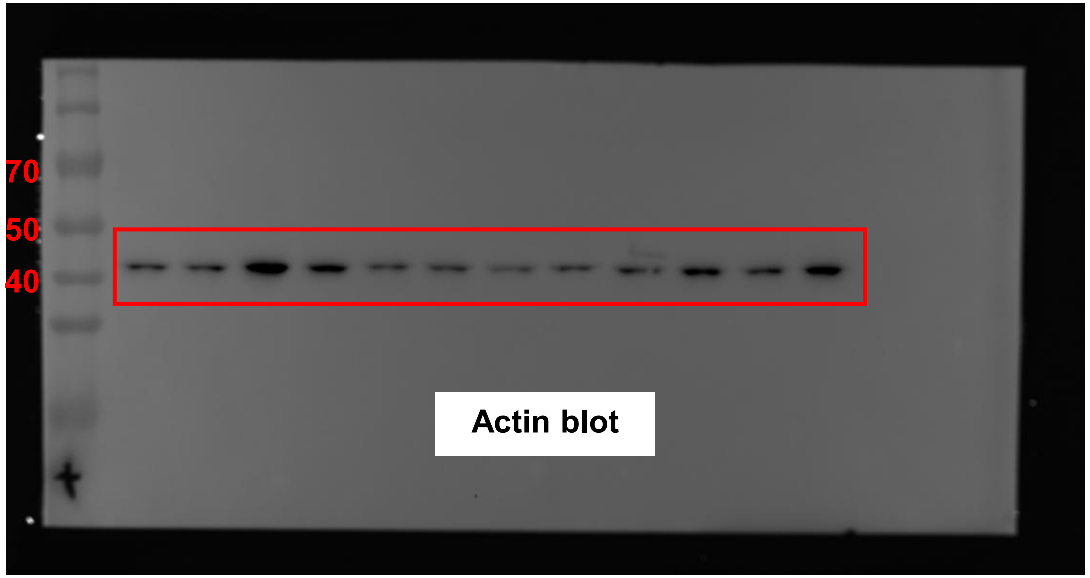

Fig S3I, K

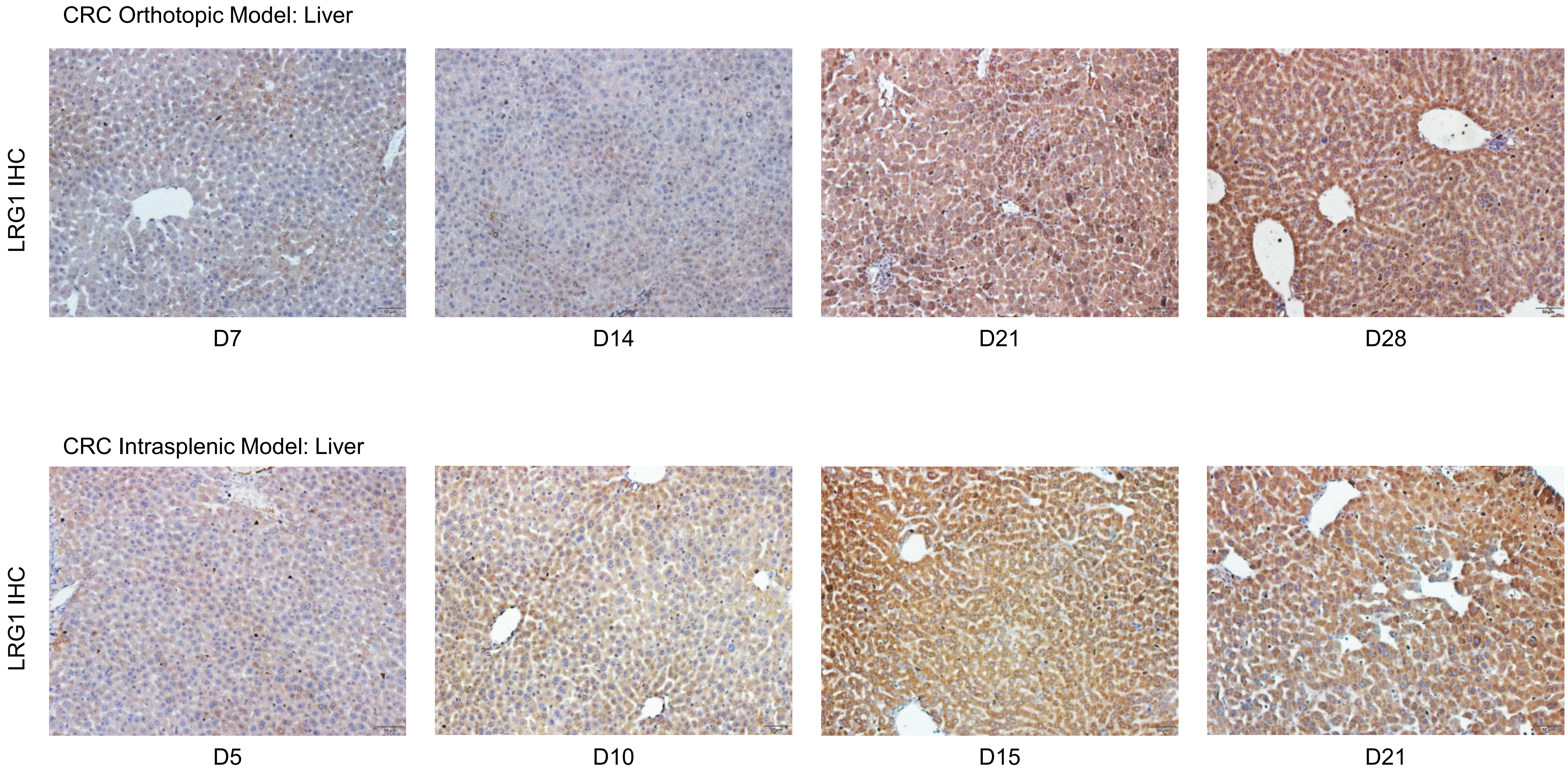
CRC Orthotopic Model: Liver
LRG1 IHC
D7
D14
D21
D28
CRC Intrasplenic Model: Liver
LRG1 IHC
D5
D10
D15
D21

Fig S3M, O

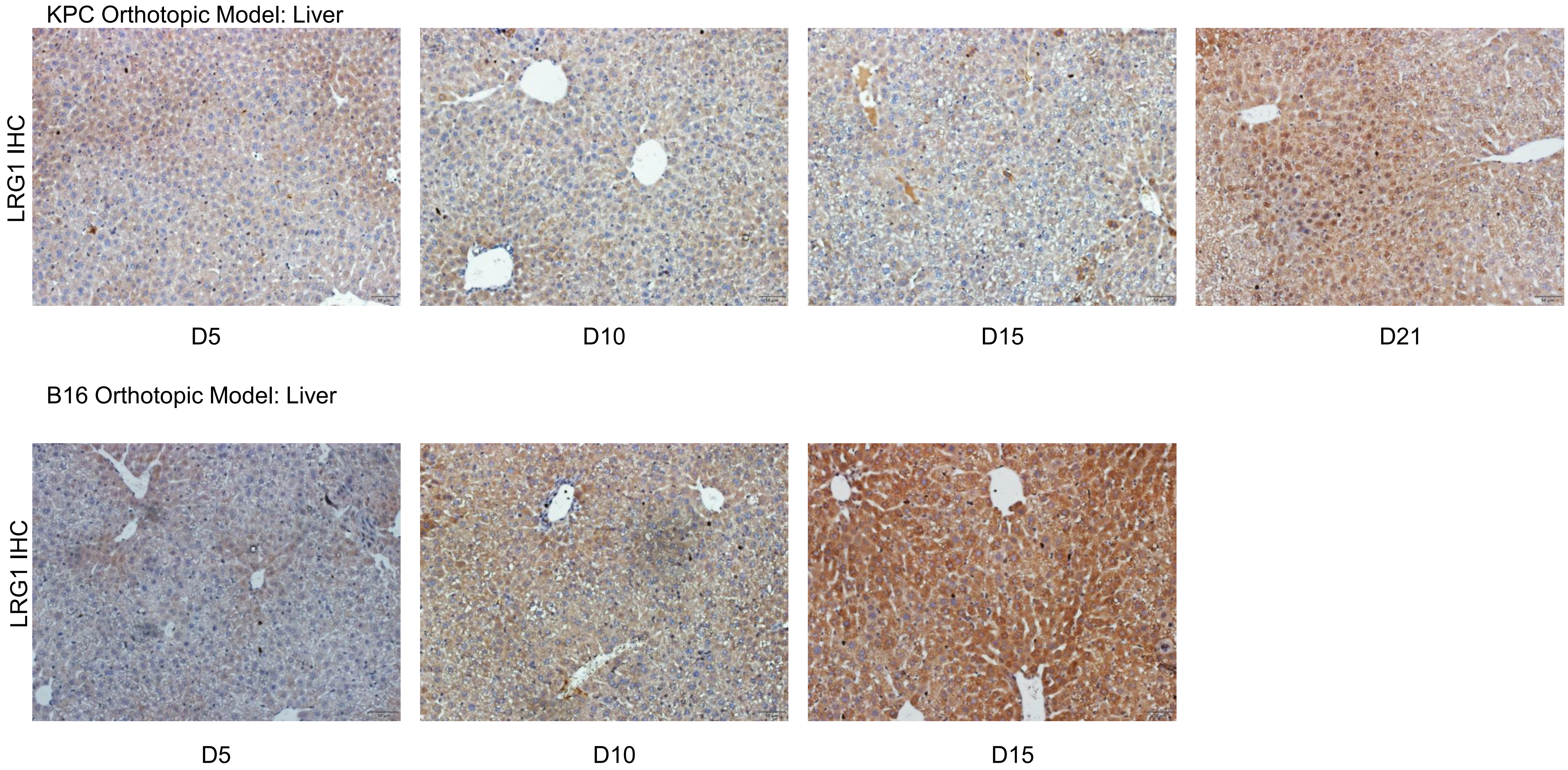
KPC Orthotopic Model: Liver
LRG1 IHC
D5
D10
D15
D21
B16 Orthotopic Model: Liver
LRG1 IHC
D5
D10
D15

Fig S4B

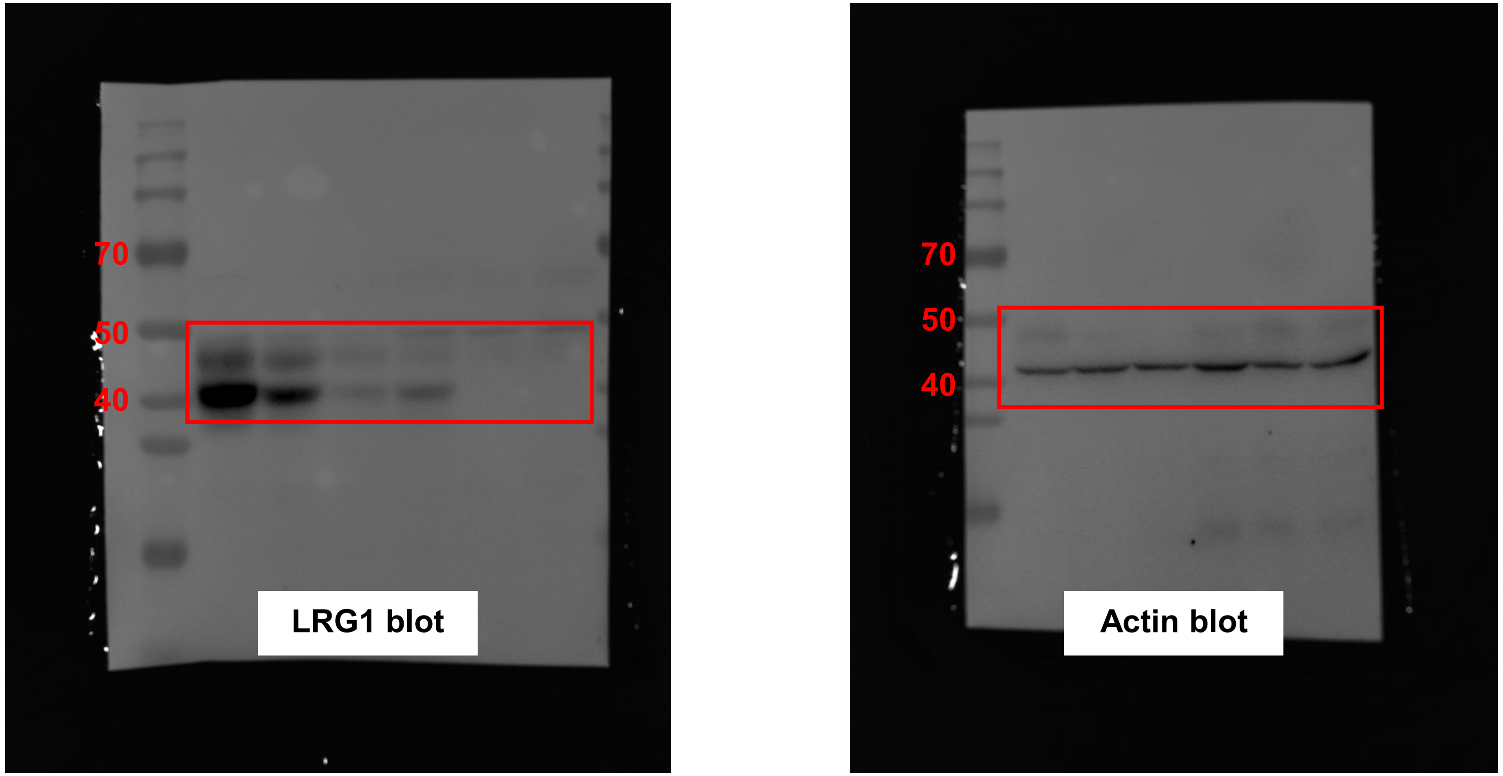

Fig S4D

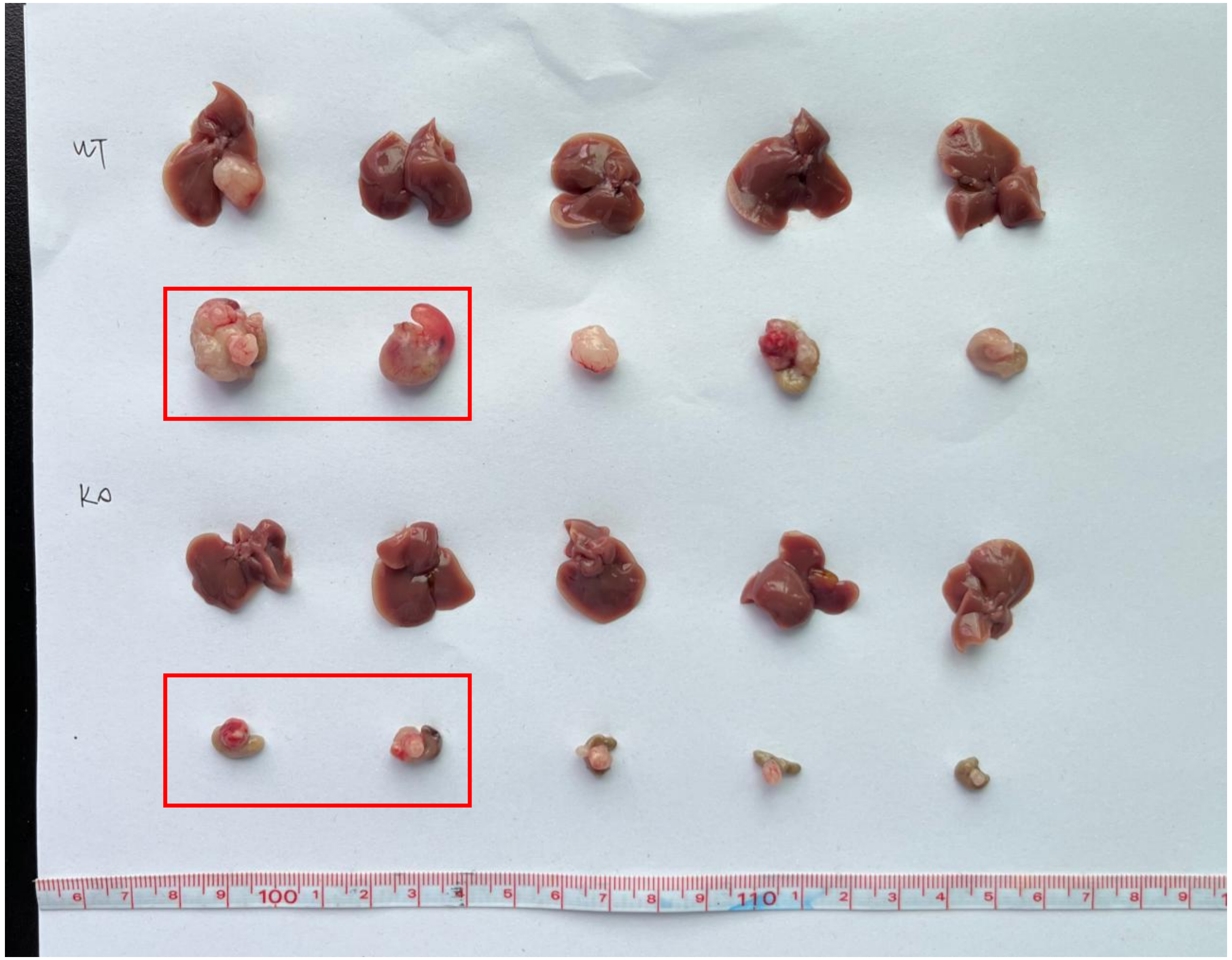

Fig S5C

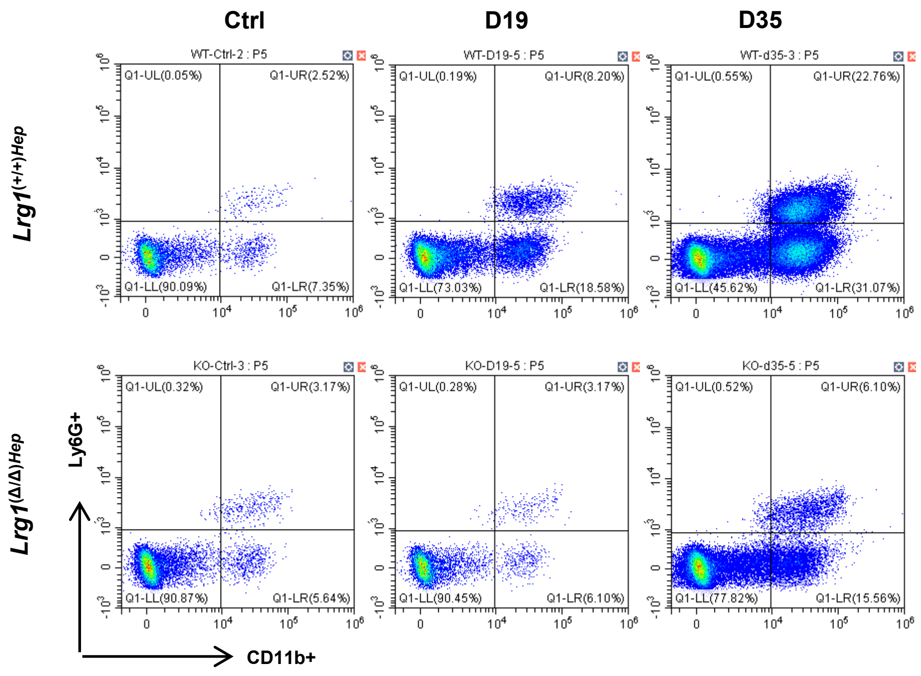
Ctrl
D19
D35
Lrg1(+/+)Hep
Lrg1(Δ/Δ)Hep
WT-Ctrl-2 : P5
Q1-UL(0.05%)
Q1-UR(2.52%)
Q1-LL(90.09%)
Q1-LR(7.35%)
WT-D19-5 : P5
Q1-UL(0.19%)
Q1-UR(8.20%)
Q1-LL(73.03%)
Q1-LR(18.58%)
WT-d35-3 : P5
Q1-UL(0.55%)
Q1-UR(22.76%)
Q1-LL(45.62%)
Q1-LR(31.07%)
KO-Ctrl-3 : P5
Q1-UL(0.32%)
Q1-UR(3.17%)
Q1-LL(90.87%)
Q1-LR(5.64%)
KO-D19-5 : P5
Q1-UL(0.28%)
Q1-UR(3.17%)
Q1-LL(90.45%)
Q1-LR(6.10%)
KO-d35-5 : P5
Q1-UL(0.52%)
Q1-UR(6.10%)
Q1-LL(77.82%)
Q1-LR(15.56%)
Ly6G+
CD11b+

Fig S6E

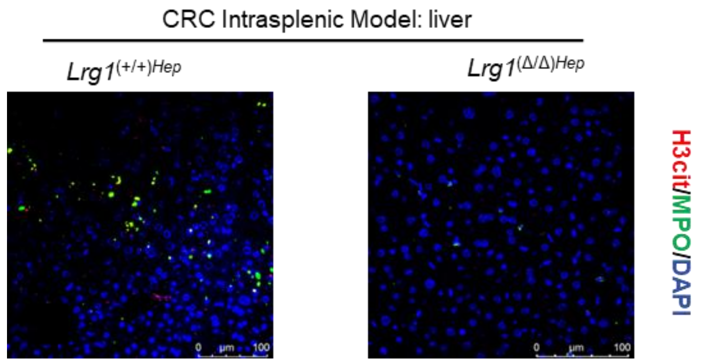
CRC Intrasplenic Model: liver
Lrg1(+/+)Hep
Lrg1(Δ/Δ)Hep
0 µm 100
0 µm 100
H3cit/MPO/DAPI

Fig S6H

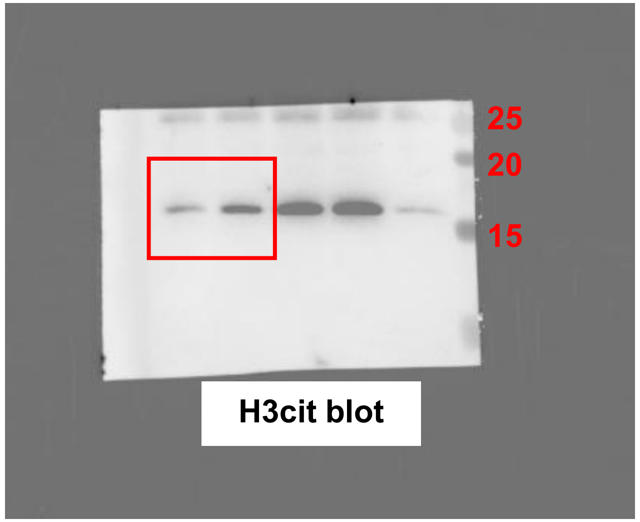

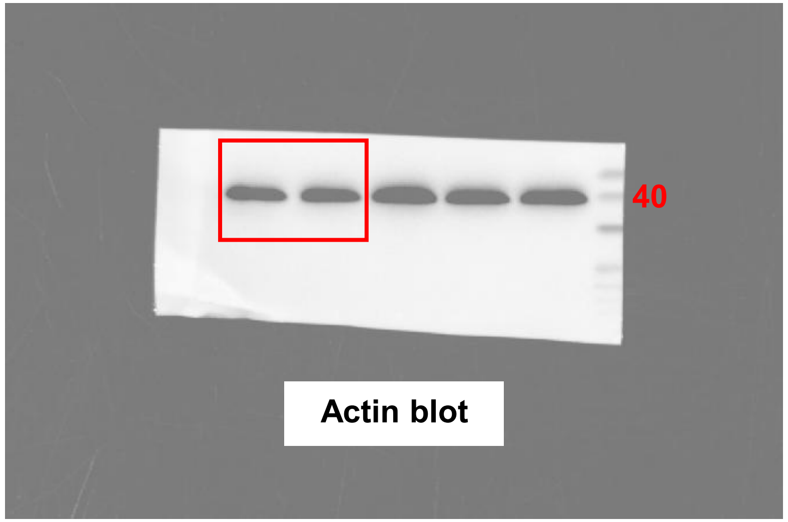

Fig S6I

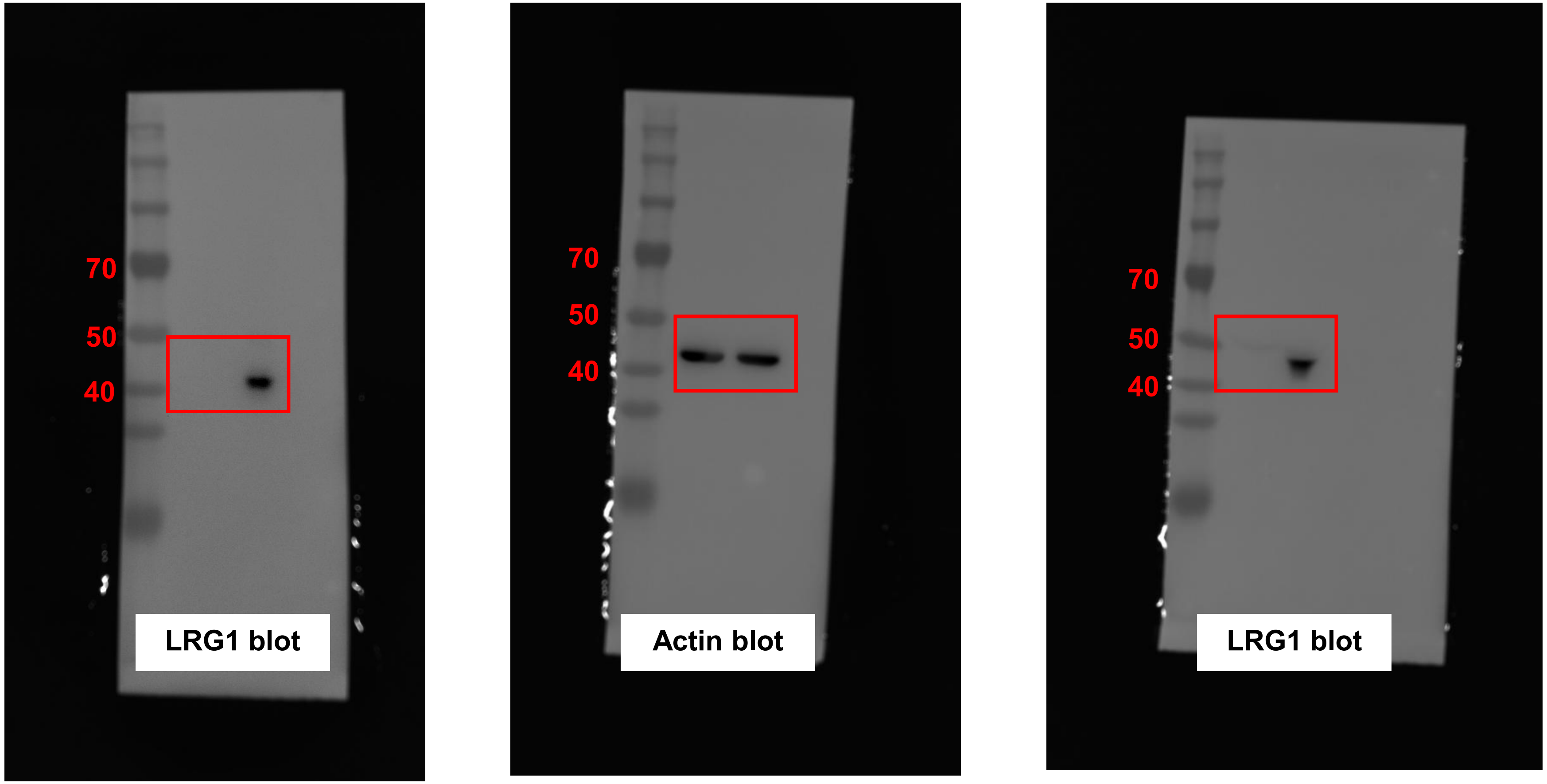

Fig S6J

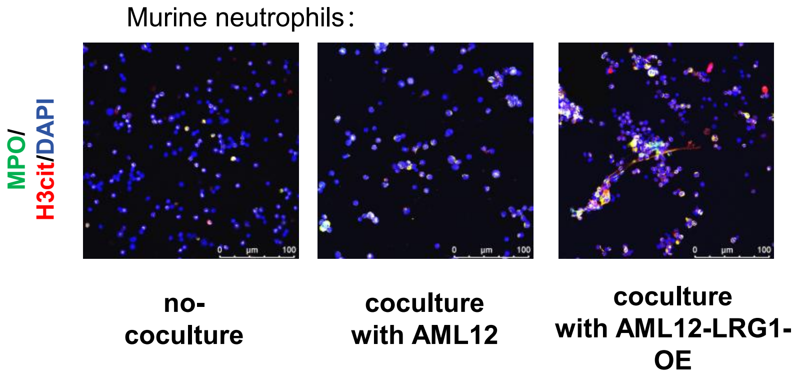
Murine neutrophils:
MPO/H3cit/DAPI
no-coculture
coculture with AML12
coculture with AML12-LRG1-OE

Fig S6K

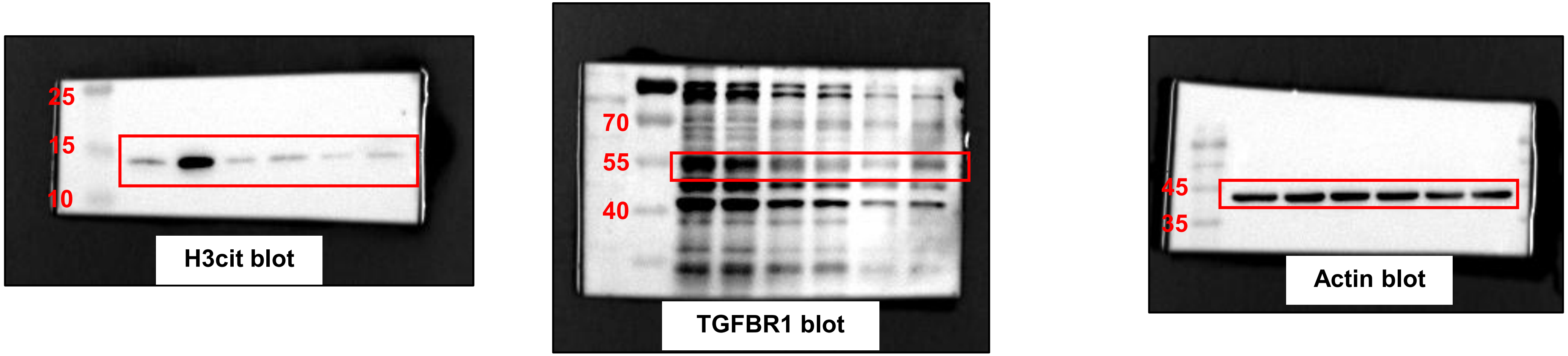

Fig S6L

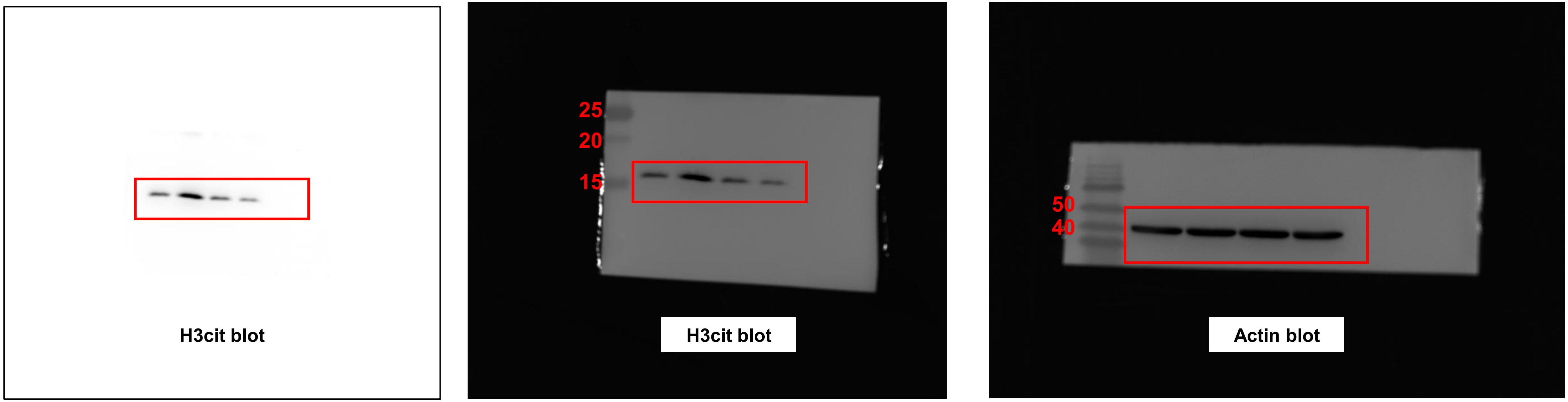

Fig S6M

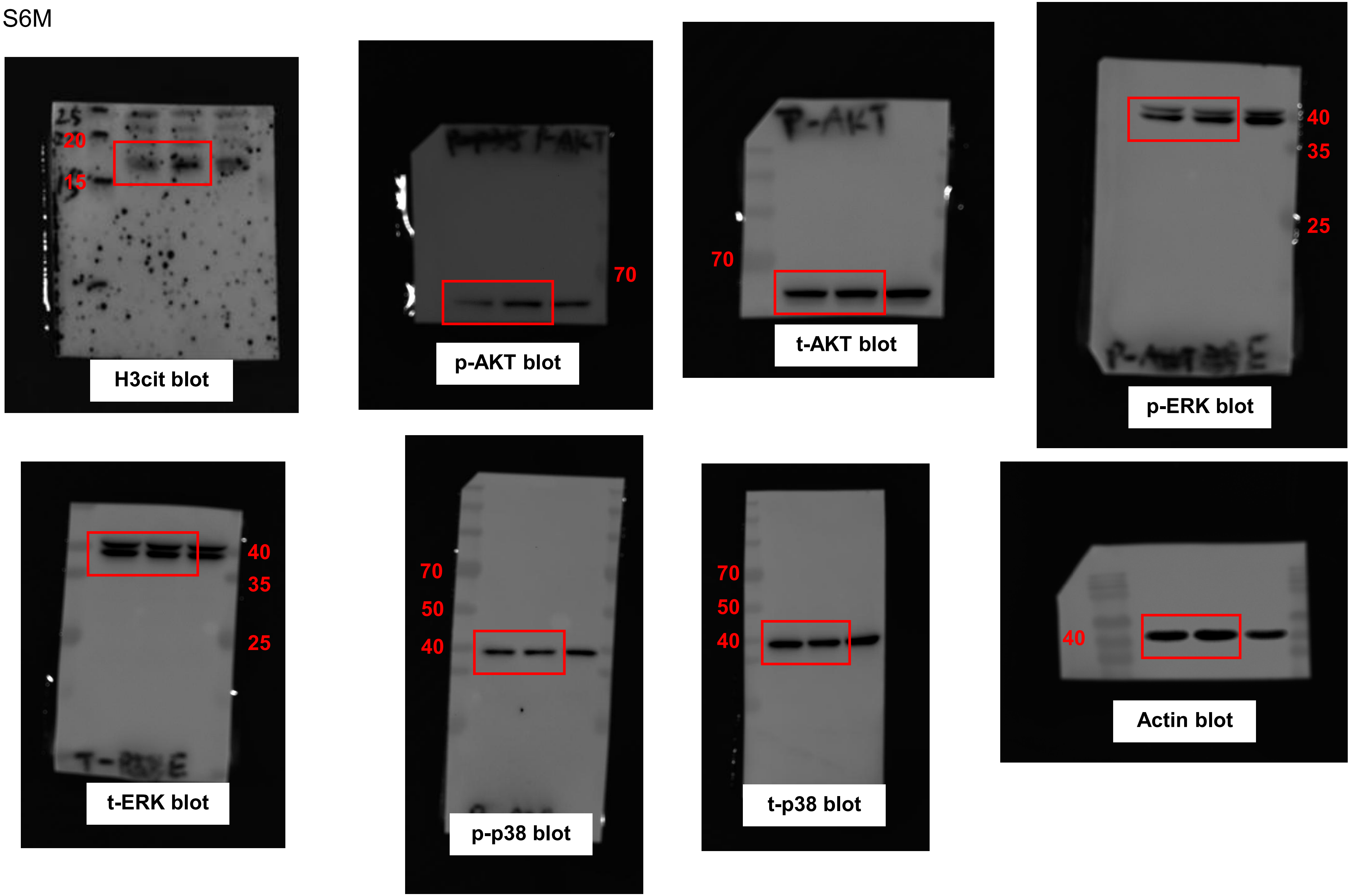
20
15
H3cit blot
70
p-AKT blot
70
t-AKT blot
40
35
25
p-ERK blot
40
35
25
t-ERK blot
70
50
40
p-p38 blot
70
50
40
t-p38 blot
40
Actin blot

Fig S6N

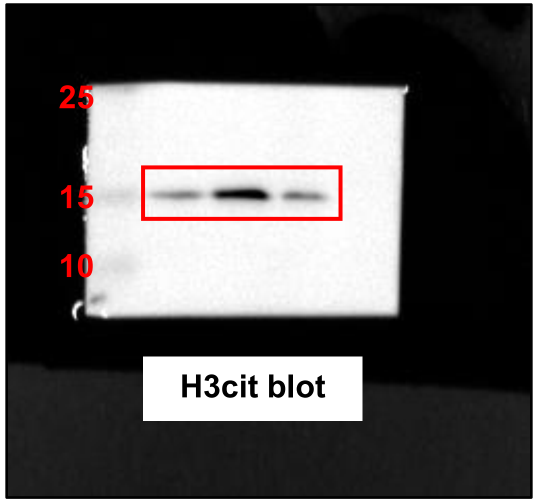
25
15
10
H3cit blot

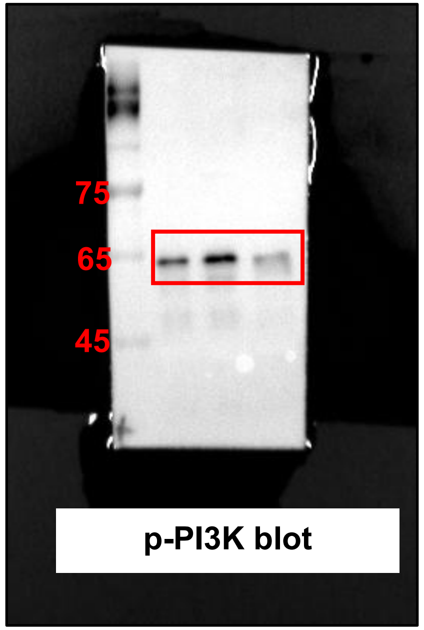
75
65
45
p-PI3K blot

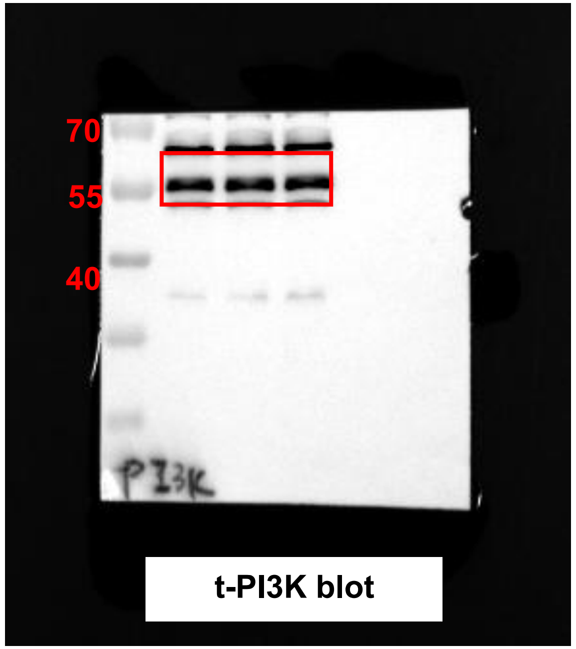
70
55
40
PI3K
t-PI3K blot

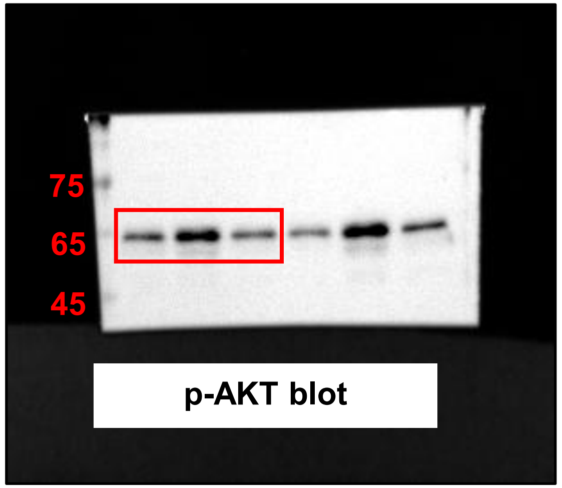
75
65
45
p-AKT blot

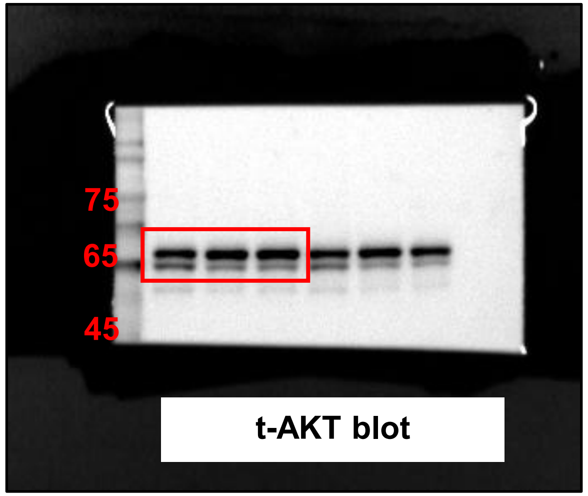
75
65
45
t-AKT blot

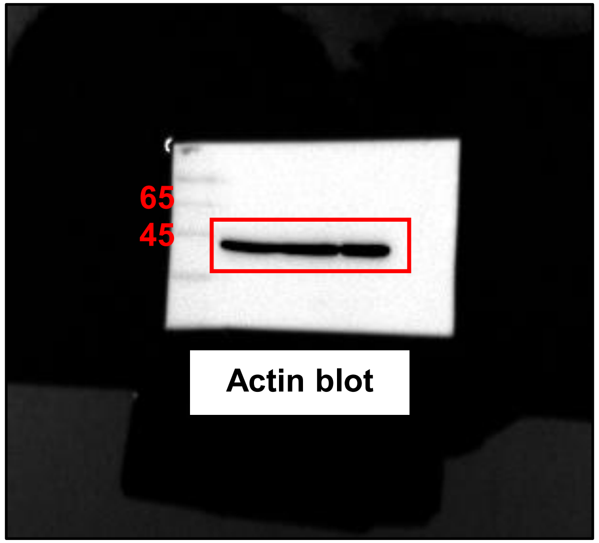
65
45
Actin blot

Fig S6O

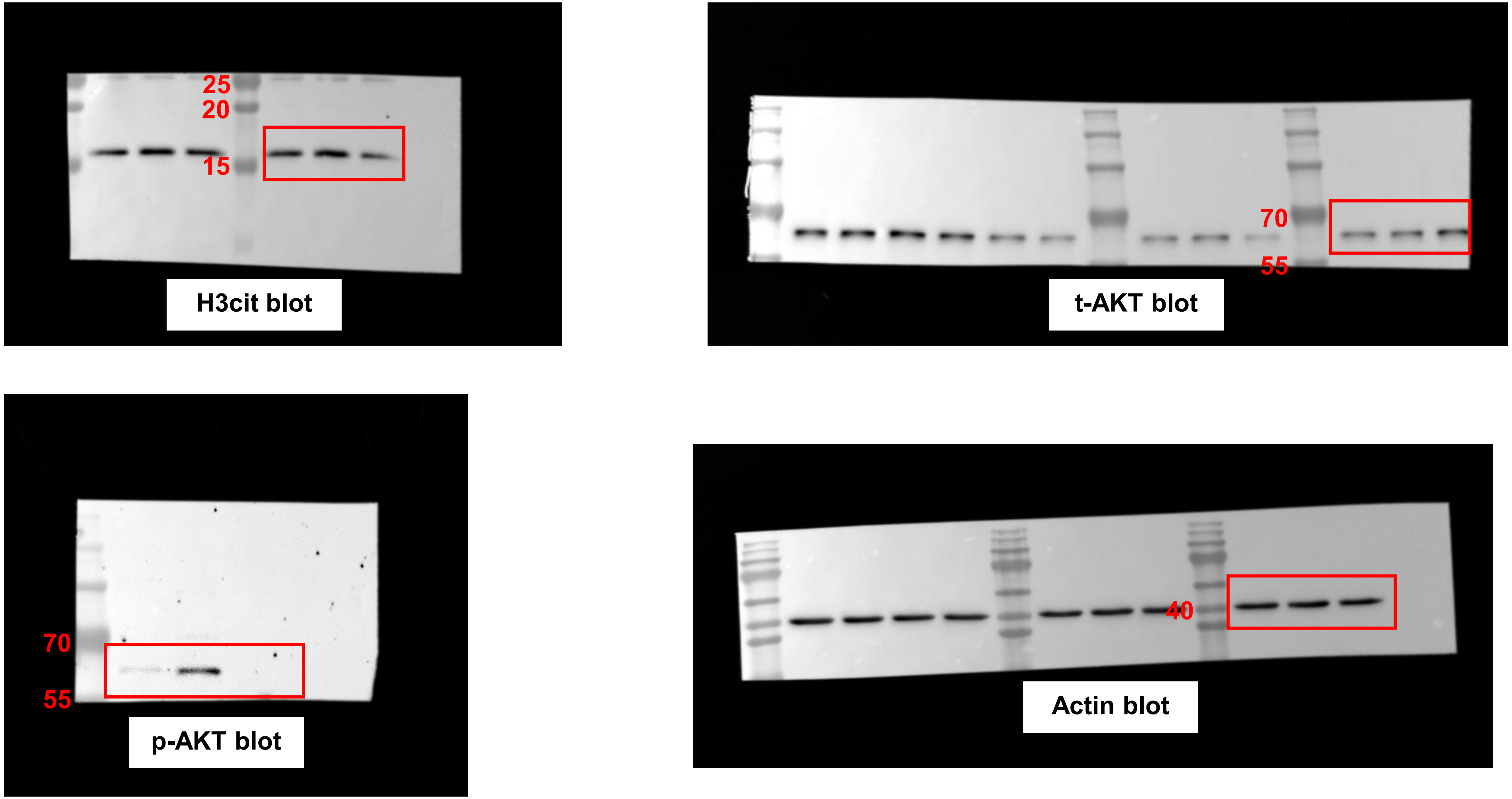

25
20
15
H3cit blot
70
55
t-AKT blot
70
55
p-AKT blot
40
Actin blot

Fig S7B

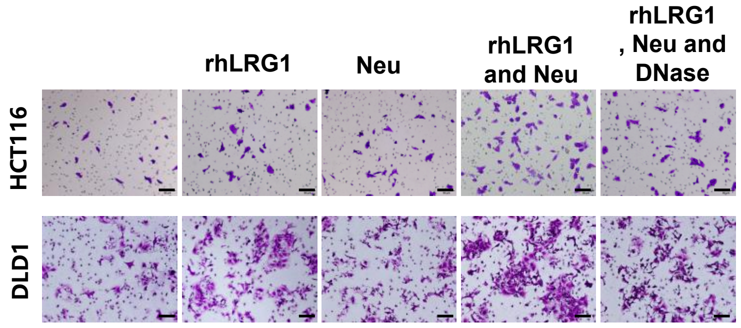

Fig S7D

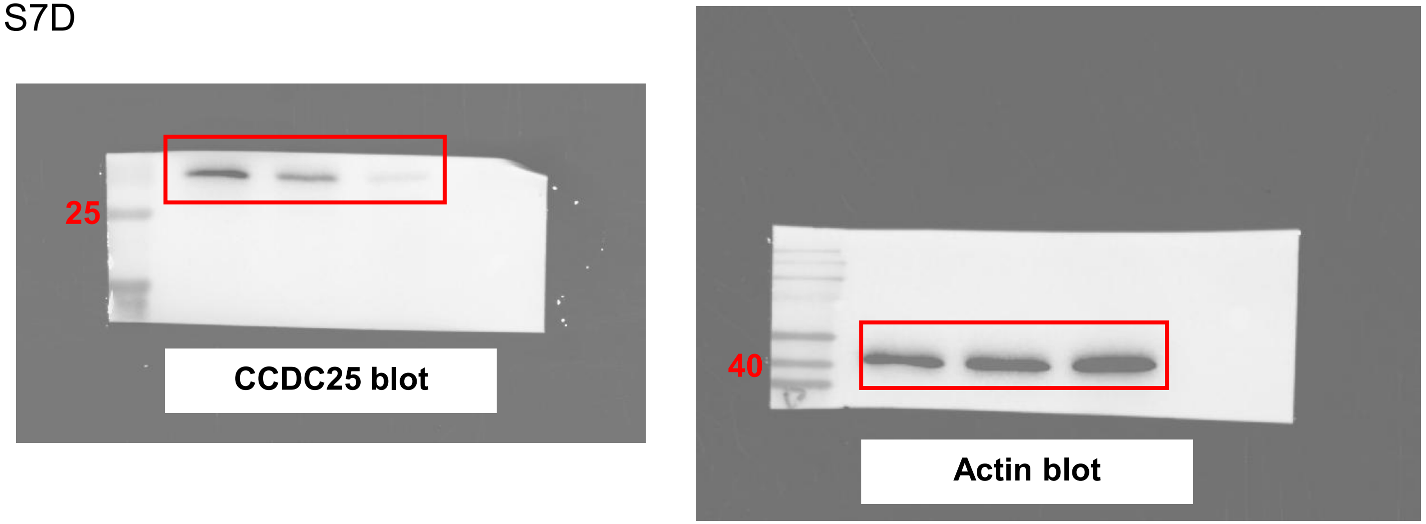

Fig S7E

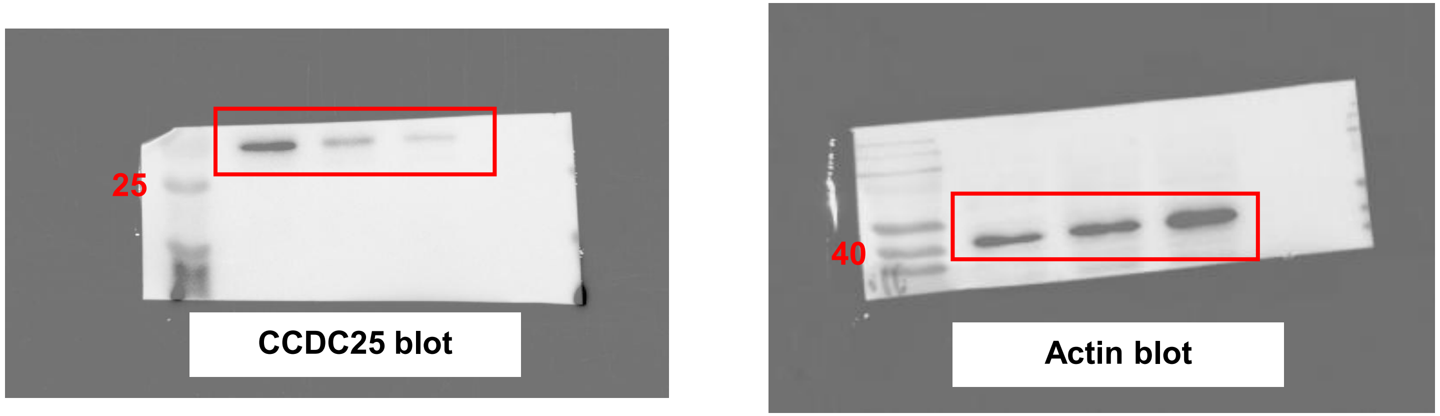

Fig S7F

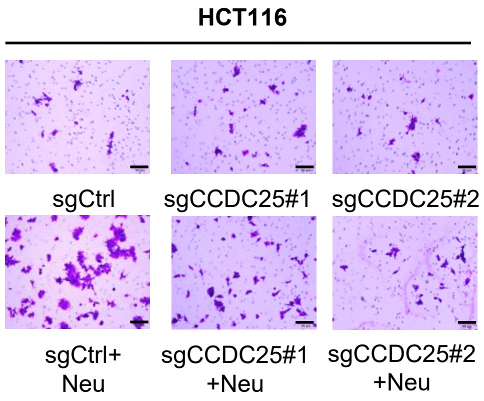

Fig S8D

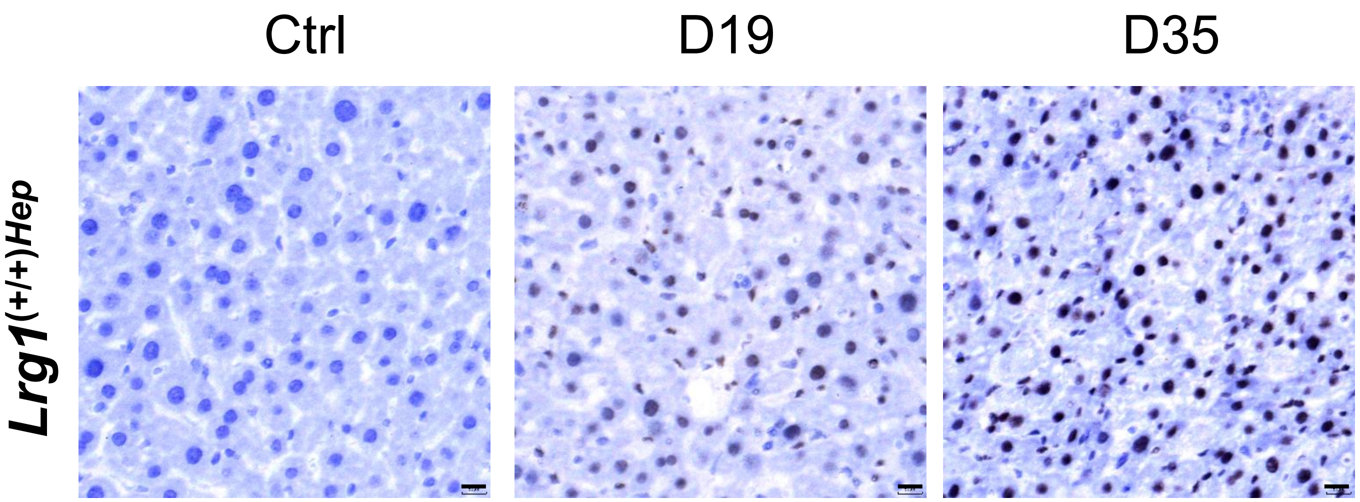

Liver: p-stat3

Fig S8L

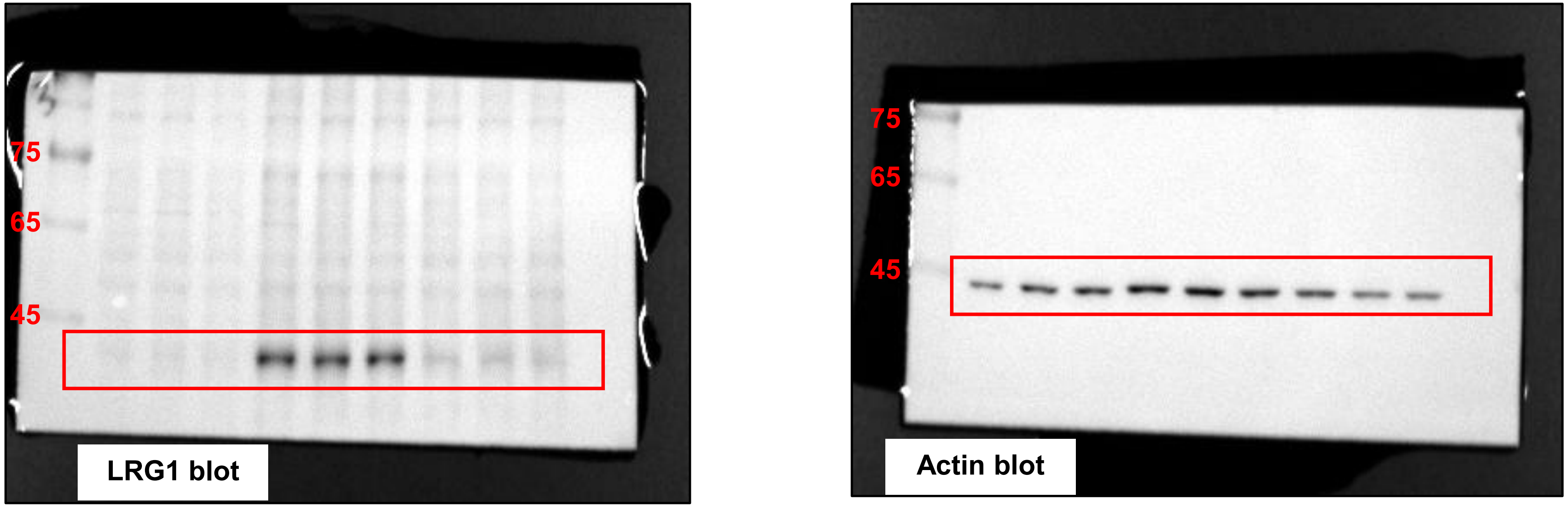

Fig S8M

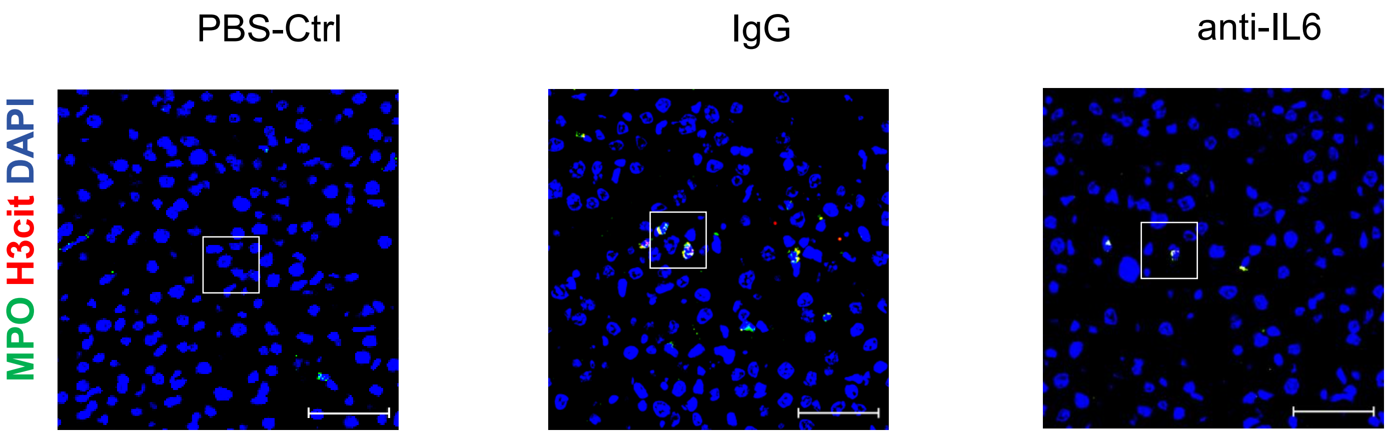

Fig S9D

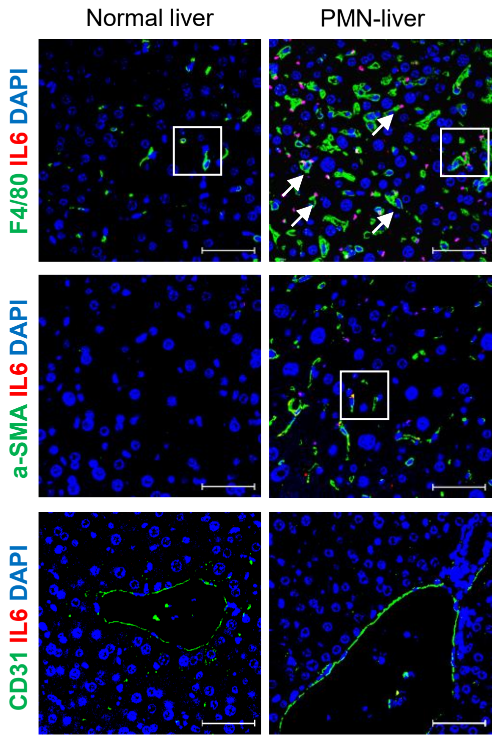

Fig S9E

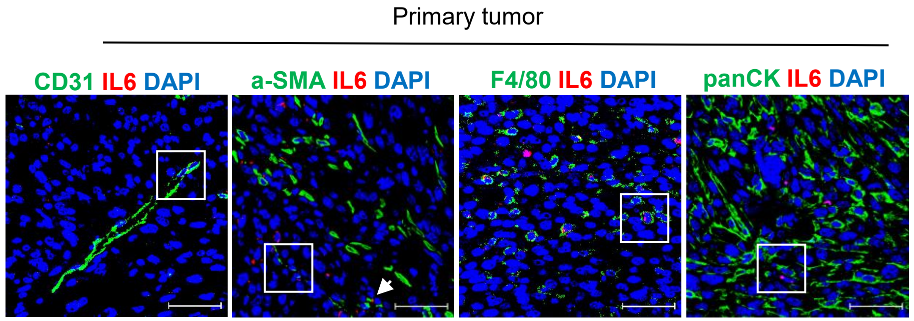

Fig S10B

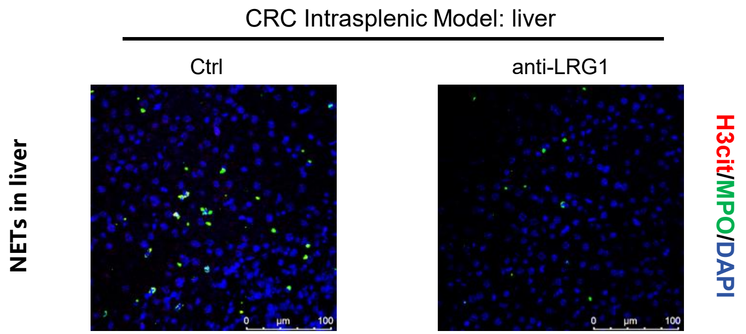

Fig S10D

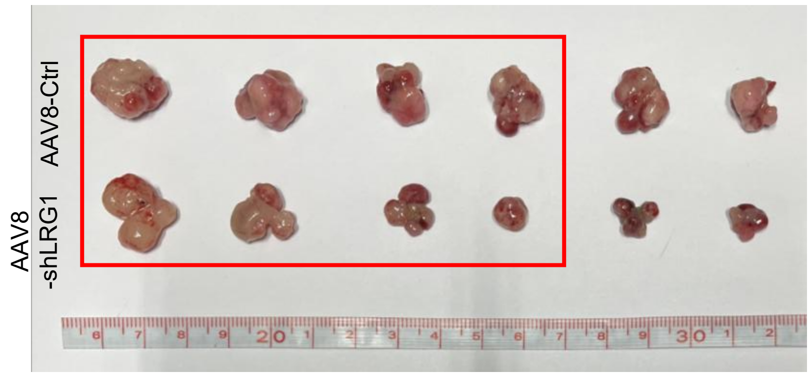

Fig S10E

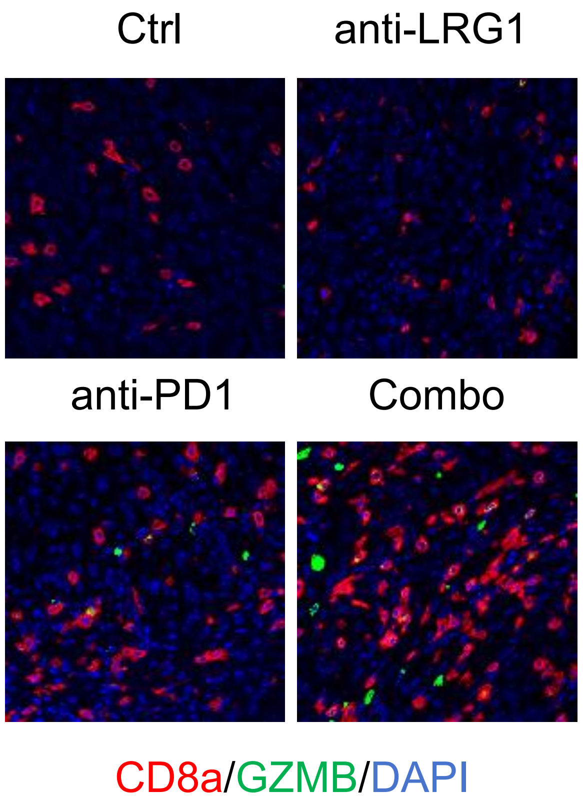

Supplement: Supplementary file 4 — Unprocessed images [file 41423_2026_1408_MOESM4_ESM.pdf]
